# Identification of amino acids involved in histamine potentiation of GABA_**A**_ receptors

**DOI:** 10.3389/fphar.2015.00106

**Published:** 2015-05-26

**Authors:** Ulrike Thiel, Sarah J. Platt, Steffen Wolf, Hanns Hatt, Günter Gisselmann

**Affiliations:** ^1^Department of Cell Physiology, Ruhr University BochumBochum, Germany; ^2^Department of Biophysics, Ruhr University BochumBochum, Germany; ^3^Department of Biophysics, Key Laboratory of Computational Biology, CAS-MPG Partner Institute for Computational Biology, Shanghai Institutes for Biological Sciences, Chinese Academy of SciencesShanghai, China

**Keywords:** GABA_A_R, histamine, modulation, site-directed mutagenesis, potentiation, salt-bridge

## Abstract

Histamine is a neurotransmitter involved in a number of physiological and neuronal functions. In mammals, such as humans, and rodents, the histaminergic neurons found in the tuberomamillary nucleus project widely throughout the central nervous system. Histamine acts as positive modulator of GABA_A_ receptors (GABA_A_Rs) and, in high concentrations (10 mM), as negative modulator of the strychnine-sensitive glycine receptor. However, the exact molecular mechanisms by which histamine acts on GABA_A_Rs are unknown. In our study, we aimed to identify amino acids potentially involved in the modulatory effect of histamine on GABA_A_Rs. We expressed GABA_A_Rs with 12 different point mutations in *Xenopus laevis* oocytes and characterized the effect of histamine on GABA-induced currents using the two-electrode voltage clamp technique. Our data demonstrate that the amino acid residues β2(N265) and β2(M286), which are important for modulation by propofol, are not involved in the action of histamine. However, we found that histamine modulation is dependent on the amino acid residues α1(R120), β2(Y157), β2(D163), β3(V175), and β3(Q185). We showed that the amino acid residues β2(Y157) and β3(Q185) mediate the positive modulatory effect of histamine on GABA-induced currents, whereas α1(R120) and β2(D163) form a potential histamine interaction site in GABA_A_Rs.

## Introduction

The GABA_A_ receptor (GABA_A_R) is the most important inhibitory receptor channel complex in the central nervous system (CNS). It is a pentameric ligand-gated ion channel, which is activated by γ-aminobutyric acid (GABA) and is modulated by various compounds. GABA_A_R modulators include benzodiazepines, barbiturates, propofol, ethanol, neurosteroids, cations (e.g., zinc ions), herbal components and odorants as well as the neurotransmitter histamine (Bureau and Olsen, 1991; McDonald and Olsen, 1994; Thompson et al., 1996; Grobin et al., 1998; Hosie et al., 2003; Kim and McDonald, 2003; Belelli and Lambert, 2005; Li et al., 2006; Saras et al., 2008; Sergeeva et al., 2010; Rudolph and Knoflach, 2011; Kletke et al., 2013; Yip et al., 2013). Since 2008, it has been known that histamine could directly activate homomeric GABA_A_Rs composed of β subunits and modulate the heteromeric GABA_A_Rs α1β2 and α1β2γ2 (Saras et al., 2008). The modulatory effects of histamine on heteromeric GABA_A_Rs depend on the subunit composition of the receptors (Bianchi et al., 2011). Today, 19 subunits are known (Simon et al., 2004). These subunits are α1–6, β1–3, γ1–3, δ, ε, 𝜃, π, and ρ1–3. Bianchi et al. (2011) showed α subunit preference of histamine under low GABA concentrations.

Histamine is a neurotransmitter in the brain and a cytokine in the periphery. It is produced and released by mast cells, basophiles, enterochromaffin-like cells, and neurons (Haas et al., 2008). In the brain histamine is released from the tuberomammillary nucleus which is involved in the wake-sleep regulation. Metabotropic histamine receptors (H_1_–H_4_) mediate the physiological functions of histamine but at least three different types of neuronal ligand-gated ion channels are modulated by histamine (Bekkers, 1993; Saras et al., 2008; Bianchi et al., 2011; Kletke et al., 2013). The possible mechanism of the modulation of GABA_A_Rs in the brain is still speculative (Saras et al., 2008; Bianchi et al., 2011).

Currently, the interaction site for histamine on the GABA_A_R is unknown. There are many known modulation sites on the GABA_A_Rs for various compounds. These sites include the amino acids β2(N265) and β2(M286). These two amino acid residues are involved in the propofol and etomidate interaction with the GABA_A_Rs (Krasowski et al., 2001; Siegwart et al., 2002; Jurd et al., 2003; Reynolds et al., 2003). These amino acid residues represent some of the different modulation mechanisms of the receptor. Propofol and etomidate are positive modulators of the GABA_A_Rs. In addition to these described modulation sites, there are other identified sites of functional relevance. These sites are, e.g., the GABA-binding site, which involves the amino acid residue F64 of the α subunit and the amino acid residues Y157 and Y205 of the β subunits (Sigel et al., 1992; Amin and Weiss, 1993). Recent studies with mutated amino acids V175, Q185, and D163 of the β subunit and R120 of the α subunit, which are components of loop nine or are involved in a salt-bridge interaction, show that these amino acids are involved in stabilizing the closed state or involved in a state-dependent salt-bridge of the GABA_A_Rs (Williams et al., 2010; Laha and Wagner, 2011).

While binding sites on the GABA_A_Rs have already been described for some allosteric modulators, the molecular basis of positive allosteric receptor modulation by histamine is unknown. Therefore, we plan to identify the amino acids that are involved in the modulatory effect of histamine at the GABA_A_Rs. For that purpose, we investigated site-directed mutated GABA_A_Rs expressed in a *Xenopus laevis* expression system using the two-electrode voltage clamp technique, and analyzed the structural impact of the chosen amino acids by creating a homology model of a 2x α1 – 3x β3 GABA_A_Rs heteropentamer.

## Materials and Methods

### GABA_A_R cDNA

Expression plasmids based on the pSGEM vector (courtesy of M. Hollmann, Bochum, Germany) for rat α1, β2, mouse γ2L, and human β3 were described by Saras et al. (2008). Rat α2 cDNA was kindly provided by ImaGenes, Berlin, Germany, and subcloned into pSGEM by using PCR and standard molecular biology methods.

The point mutations were made using overlap-extension PCRs, as described by Heckman and Pease (2007). For overlap-extension PCRs, the plasmids for α1, β2, and β3 subunits were used as templates. Overlap-extension PCR was performed using a mixture of *taq*-DNA- and *pwo*-DNA-polymerase (20:1) (Biotherm) in a volume of 50 μl with 20 pmol of each primer (Supplementary Table S3). The following temperature cycle profile was used: 5 min at 95°C; followed by 30 cycles of 45 s at 95°C, 45 s at 60°C, 60 s at 72°C (120 s for the fusion PCR); and a final extension of 10 min at 72°C. The mutated DNA was subcloned into pSGEM using standard molecular biology methods.

### Expression of Receptor cRNA in *Xenopus laevis* oocytes

The cRNAs of wt as well as point mutated GABA_A_R subunit cRNAs were synthesized by using the AmpliCap T7 high yield message maker kit (Epicentre, Madison, WI, USA) as described by Sergeeva et al. (2010). The oocytes were prepared from *Xenopus laevis* using standard methods. Five to twenty nanograms of cRNA was injected, 24 h after surgery, into one stage V–VI oocyte. The incubation took place at 12–16°C in ND96 [96 mM NaCl, 2 mM KCl, 1.8 mM CaCl_2_, 1 mM MgCl_2_, 5 mM HEPES, pH 7.2; 100 U/ml penicillin, 100 U/ml streptomycin from Antibiotic Antimycotic Solution (100x)] (Sigma–Aldrich, St. Louis, MO, USA). After 2–4 days, the oocytes were measured by two-electrode voltage clamp, as briefly described by Sergeeva et al. (2010). Each of the tested substances was diluted with Frog-Ringer’s solution (115 mM NaCl, 2.5 mM KCl, 1.8 mM CaCl_2_, 10 mM HEPES, pH 7.2). The agonists were dissolved in ND96 and applied in a volume of 200 μl into the entrance tube of the recording chamber, totally exchanging the bath solution within a second. Therefore, due to the relative slow desensitization kinetics in our investigated GABA receptors and the fact that we compare the same receptor ± histamine, desensitization will not interfere with the determination of the histamine potentiation. The pH of GABA- or histamine-containing solutions was monitored to ensure a pH of 7.2 for all histamine concentrations. All measurements were taken with a membrane potential of -40 mV.

### Analysis and Statistics

During the measurements, the currents were recorded using CellWorks software and were analyzed using pCLAMP 10 software. The statistical evaluation and curve fitting (3-parameter Hill equation) was performed using SigmaPlot V8.0 (Systat Software, San Jose, CA, USA). All mean values are ± SEM, and the significant data were marked with ^∗^*p* ≤ 0.05, ^∗∗^*p* ≤ 0.01, and ^∗∗∗^*p* ≤ 0.001.

### GABA_A_R Homology Modeling and Histamine Docking

The 2.97 Å crystal structure of the GABA β3 homopentamer (Miller and Aricescu, 2014) was used as basis for homology modeling. We carried out a sequence alignment of the α1 sequence (Uniprot entry P14867, The UniProt Consortium, 2013) and the β3 subunit sequences from the crystal structure with MUSCLE (Edgar, 2004; default settings). The resulting alignment showed 35.3% sequence identity and 61.9% sequence similarity and is included in the Supplementary Material. As we know from GPCR modeling, an identity >35% is a good basis for homology modeling of transmembrane proteins (Wolf et al., 2008; Kufareva et al., 2011). Based on this alignment, we created and optimized a model of an α1 subunit homopentamer with the SWISS-MODEL server (Biasini et al., 2014), based on the structure of the β3 homopentamer from the crystal structure mentioned above. We then created a 2x α1 – 3x β3 GABA_A_R heteropentamer by substituting two β3 subunits in the crystal structure (chain A and C) by the respective α1 subunits in PyMol (Schrödinger, 2010). We then carried out a WHATIF protein check, showing that the model is reliable. The checkfile can be found in the Supplementary Material. Binding site analysis was carried out with TRIDOCK (te Heesen et al., 2007). In short, TRIDOCK searches for putative binding positions for small organic molecules following Congreve’s rule-of-three (Congreve et al., 2003) and marks these positions with a “bead.” Docking calculations on histamine binding were carried out with Autodock Vina (Trott and Olson, 2010), using a 20 × 20 × 20 Å box (grid spacing 1 Å) centered between Asp163(β) and R120(α), and an exhaustiveness parameter of 80 (Schneider et al., 2011). A histamine topology was obtained from the PRODRG server (Schuttelkopf and van Aalten, 2004). Polar atoms and Kollman charges were added to the heteropentameric protein, while histamine was docked with Gasteiger charges.

## Results

### Screening of Mutated GABA_A_Rs for Altered Histamine Potentiation

Saras et al. (2008) showed that homomeric β2 or β3 receptors were activated by histamine in a recombinant expression system. Our intention was to analyze the effect of histamine on point mutated GABA_A_R β2/3 subunits. First, we generated mutations in β2/3 subunits. In contrast to the wild-type (wt) subunits, functional expression of mutated GABA_A_Rs as homomeric receptors failed in most instances so that a systematic screening of homomeric receptors was not possible. Therefore, we had to determine the impact of mutated amino acids in heteromeric receptors by analyzing the potentiating effect of histamine on the GABA-induced currents. We were mostly interested in the effect of histamine on the two most important synaptic GABA_A_Rs of the CNS composed of α1β2γ2L and α2β3γ2L subunits (Pirker et al., 2000; Nutt, 2006; Olsen and Sieghart, 2008).

To screen the modulating effect of histamine, we investigated the action of 3 mM histamine on currents evoked by GABA (typically EC_10-30_). To determine suitable GABA concentrations for screening experiments, the EC_50_ values of GABA for all of the investigated subunit combinations were determined (Supplementary Table S1). In addition, we tested histamine concentrations up to 10 mM to demonstrate the absence of homomeric GABA_A_Rs composed of β subunits. For all of our analyzed GABA_A_Rs, histamine alone did not induce any currents (data not shown).

**FIGURE 1:**
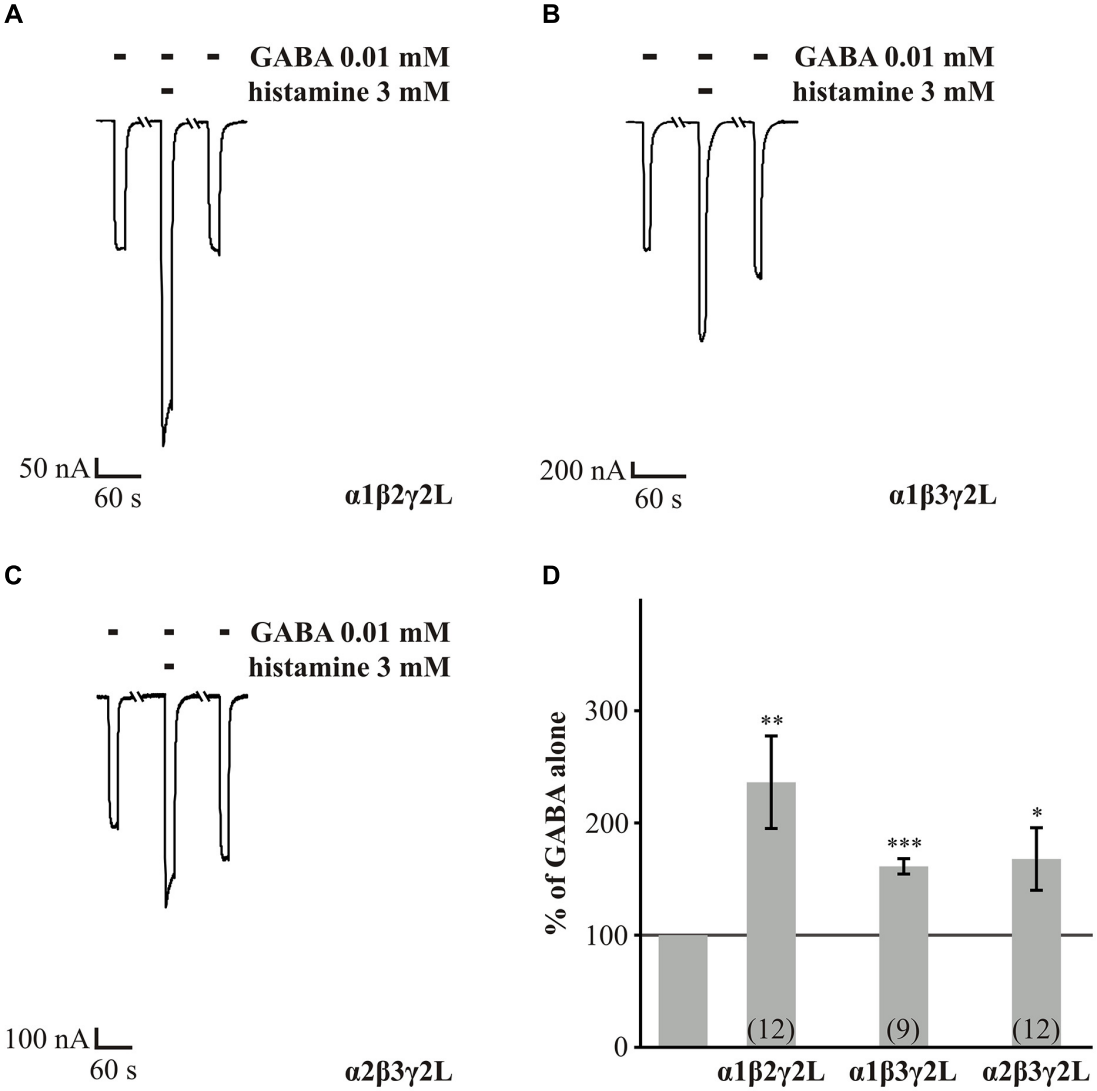
**GABA potentiated by histamine in wt receptors**. GABA-induced (EC_10-30_) currents are potentiated by 3 mM histamine. (A–C) *Xenopus* oocytes expressing the abg isoforms: α1β2γ2L, α1β3γ2L, and α2β3γ2L were voltage clamped, and GABA at a concentration of 10 μM was bath-applied with or without 3 mM histamine. **(D)** Average potentiation of GABA-induced currents calculated from measurements as shown in **(A–C)**. Bars two to four show the mean enhancement ± SEM, as a percentage of the amplitude of GABA alone (gray line), for the αβγ isoforms: α1β2γ2L, α1β3γ2L, and α2β3γ2L, (*n* = 9–12). Significant data were marked with ^∗^*p* ≤ 0.05, ^∗∗^*p* ≤ 0.01, and ^∗∗∗^*p* ≤ 0.001.

First, we analyzed the wt GABA_A_Rs α1β2γ2L, α1β3γ2L, and α2β3γ2L, to demonstrate a robust and reproducible potentiation of GABA-induced currents by histamine under our experimental conditions. This analysis also served as control for our experiments with the point mutated GABA_A_Rs. With our experiments, we could show, as Saras et al. (2008) had previously, that histamine could increase the GABA-induced current of wt receptors in a dose-dependent manner. Three millimolar histamine significantly potentiated the GABA-induced currents of GABA_A_Rs α1β2γ2L by approximately 1.4-fold, α1β3γ2L by approximately 0.6-fold and α2β3γ2L by approximately 0.7-fold (**Figure 1**).

**FIGURE 2:**
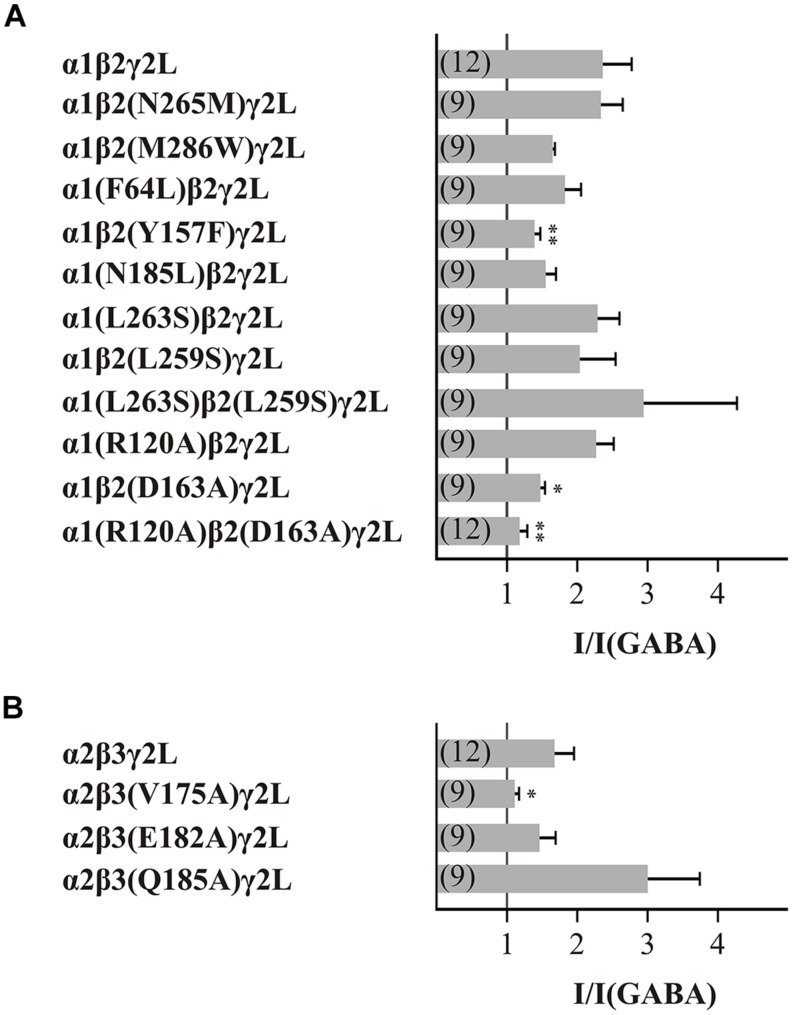
**Effect of 3 mM histamine on the GABA-induced current of wt or mutated GABA_A_Rs**. The bars show the increase of the GABA-induced (typically EC_10-30_) currents by 3 mM histamine of the GABA_A_R subunit combination **(A)** α1β2γ2L and **(B)** α2β3γ2L. Significant data were marked with ^∗^*p* ≤ 0.05 and ^∗∗^*p* ≤ 0.01.

Next, we screened all point mutated GABA_A_Rs using the same conditions: a GABA concentration of typical EC_10-30_ and a histamine concentration of 3 mM. The first amino acids to be analyzed for their action in the histamine modulation of the GABA_A_R were the point mutants β(N265M) and β(M286W). These amino acids are involved in the action of etomidate and propofol on the GABA_A_Rs (Krasowski et al., 2001; Siegwart et al., 2002; Jurd et al., 2003; Reynolds et al., 2003). Our experiments showed that the GABA-induced currents (10 μM GABA) on GABA_A_Rs that contained the point mutated subunits β(N265M) or β(M286W) were increased by histamine (**Figure 2**). However, the histamine modulation of the mutated receptors was not significantly different from the modulation of the wt GABA_A_Rs α1β2γ2L. Therefore, these mutations do not influence histamine modulation.

The next 10 amino acids that we analyzed are important for binding GABA, for forming a state-dependent salt-bridge or for stabilizing the closed state of the GABA_A_Rs (Amin and Weiss, 1993; Williams et al., 2010; Laha and Wagner, 2011). Furthermore, we were interested in the influence of the conserved leucine in the pore-forming TM2 segment that is involved in the gating-mechanism of the GABA_A_Rs (Chang and Weiss, 1999). Because this amino acid, β2(L259), is also present in α subunits, we investigated point mutations of both a and β subunits. Receptors with the mutations β2(Y157F), β2(D163A), β3(V175A), and the combination of α1(R120A) with β2(D163A) showed significantly reduced potentiation of the GABA-induced current by histamine (**Figure 2**).

It can be suggested that the amino acids Y157, D163, and V175 of the β subunit and the amino acid R120 of the α subunit are involved in the action of histamine. The analyzed GABA_A_R α2β3(Q185A)γ2L showed a stronger potentiation than the wt demonstrating that the amino acid Q185 also potentially affects histamine action on GABA_A_Rs.

### Dose-Dependent Action of Histamine on Mutated GABA_A_Rs

To investigate the impact of the identified amino acids on histamine modulation in more detail, we next analyzed the effect of different histamine concentrations (0.1 to 10 mM) on the GABA-induced current (typically EC_10-30_).

**FIGURE 3:**
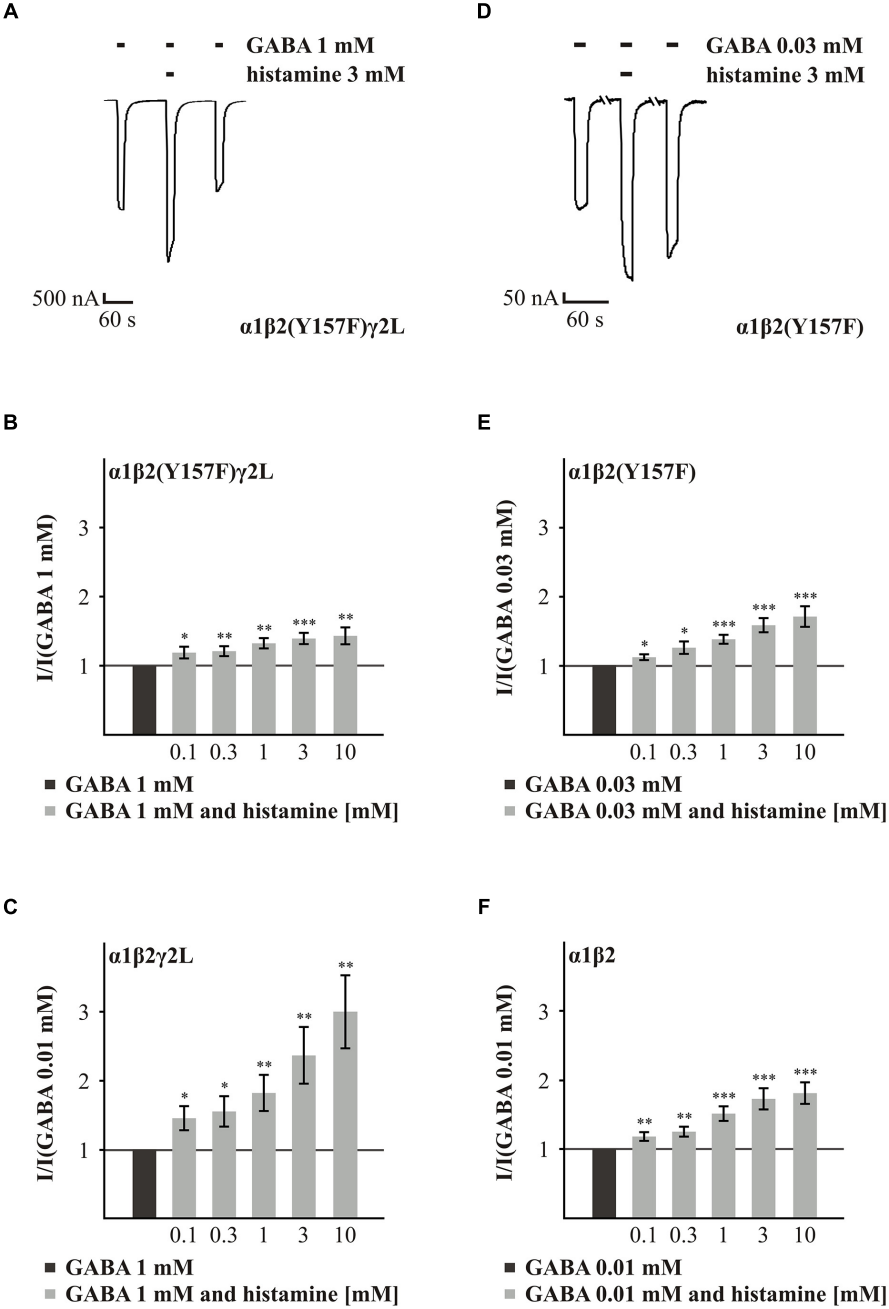
**Histamine action on GABA-induced currents of GABA_A_Rs with the point mutated β2(Y157F) subunit. (A,D)** Original traces of the histamine (3 mM) action on the GABA-induced currents of the GABA_A_Rs α1β2(Y157F)γ2L and α1β2(Y157F). **(B,C,E,F)** Bar diagrams show the action of various histamine concentrations on the GABA-induced currents of the GABA_A_Rs α1β2(Y157F)γ2L, α1β2(Y157F), α1β2γ2L, and α1β2. Significant data were marked with ^∗^*p* ≤ 0.05, ^∗∗^*p* ≤ 0.01, and ^∗∗∗^*p* ≤ 0.001.

The first amino acid residue we analyzed was Y157 of the β subunit. This amino acid residue is involved in the conformational changes in the GABA_A_Rs induced by GABA (Amin and Weiss, 1993). GABA_A_R α1β2(Y157F)γ2L showed reduced potentiation of the GABA-induced current by 3 mM histamine (**Figure 2A**) in comparison to the wt α1β2γ2L receptor. Histamine concentrations of 0.1 to 10 mM potentiated the GABA-induced current in a dose-dependent manner (**Figures 3A,B**). However, for all of the tested concentrations, the potentiating effect was smaller than the effect observed with the wt receptor (**Figures 3B,C**, and Supplementary Table S2). To analyze the influence of the γ2L subunit, we co-expressed the subunits α1 and β2(Y157F). Histamine caused a dose-dependent potentiation of the mutated GABA_A_R (**Figures 3D,E**) that was not significantly different from the wt α1β2 receptor (**Figures 3E,F**, and Supplementary Table S2). Interestingly, the influence of the point mutations is dependent on the presence of the γ2L subunit. These results show that Y157 is not essential for potentiation by histamine.

**FIGURE 4:**
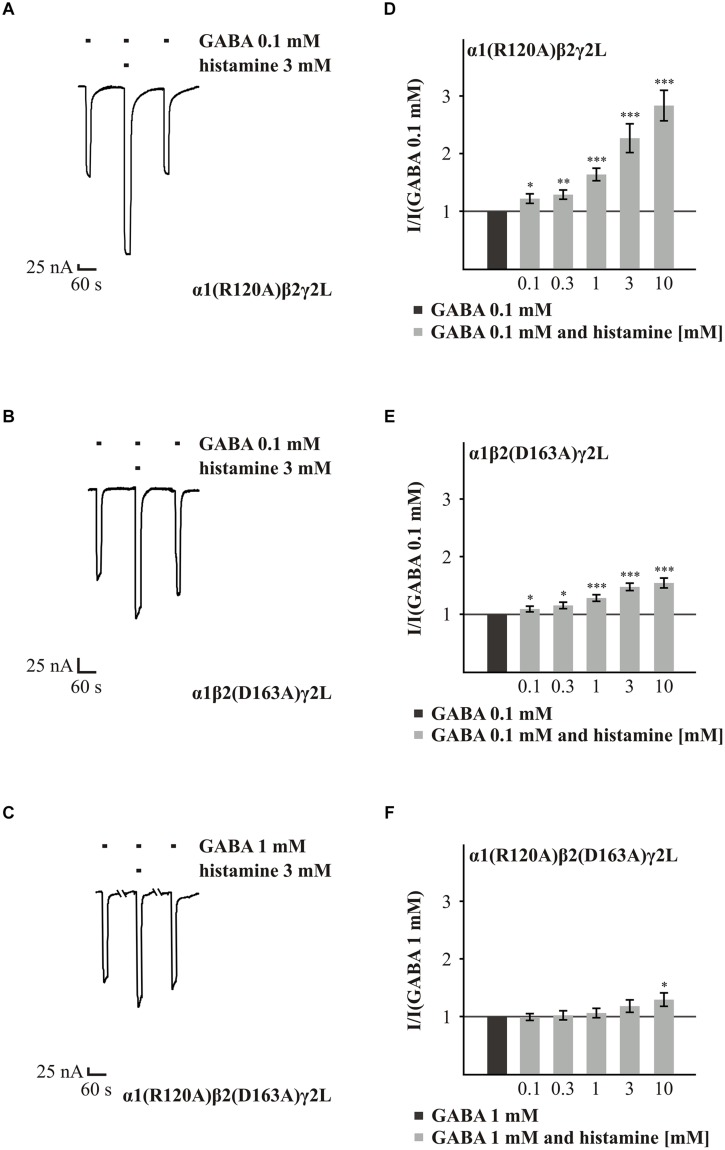
**Histamine action on GABA-induced currents of GABA_A_Rs with point mutated subunits α1(R120A) and β2(D163A). (A–C)** Original traces of the modulatory effect of 3 mM histamine on GABA-induced currents from GABA_A_Rs α1(R120A)β2γ2L, α1β2(D163A)γ2L, and α1(R120A)β2(D163A)γ2L. **(D–F)** Bar diagrams show the effect of 0.1 to 10 mM histamine on the GABA-induced (typically EC_10-30_) current. Significant data were marked with ^∗^*p* ≤ 0.05, ^∗∗^*p* ≤ 0.01, and ^∗∗∗^*p* ≤ 0.001.

Next, we analyzed the effect of histamine on the amino acids α1(R120) and β2(D163), which form a state-dependent salt-bridge of the GABA_A_Rs (Laha and Wagner, 2011). We investigated the ability of different histamine concentrations (0.1 to 10 mM) to potentiate the GABA response (typically EC_10-30_) on the GABA_A_Rs α1(R120A)β2γ2L, α1β2(D163A)γ2L, and α1(R120A)β2(D163A)γ2L (**Figure 4**). Histamine shows a dose-dependent potentiation on the GABA_A_Rs α1(R120A)β2γ2L and α1β2(D163A)γ2L (**Figures 4A,B,D,E**). However, at a receptor composed of both mutated subunits in combination with the γ2L subunit, the increasing effect of histamine is nearly abolished (**Figures 4C,F**). In comparison to wt, the mutation α1(R120A) alone had no significant effect on histamine potentiation (Supplementary Table S2). At GABA_A_Rs α1β2(D163A)γ2L and GABA_A_R α1(R120A)β2(D163A)γ2L (**Figure 3C**), histamine potentiation was significantly less than at the wt for most concentrations tested (Supplementary Table S2). To ensure that this effect is specific to histamine and does not abolish potentiation in general, we tested the potentiation of the GABA-induced currents by 10 μM propofol (Yip et al., 2013), which was able to potentiate the GABA-induced current (data not shown). To verify the influence of the γ2L subunit, we attempted to express the subunit combinations α1(R120A)β2, α1β2(D163A), and α1(R120A)β2(D163A), but none of these combinations produced a functional receptor.

**FIGURE 5:**
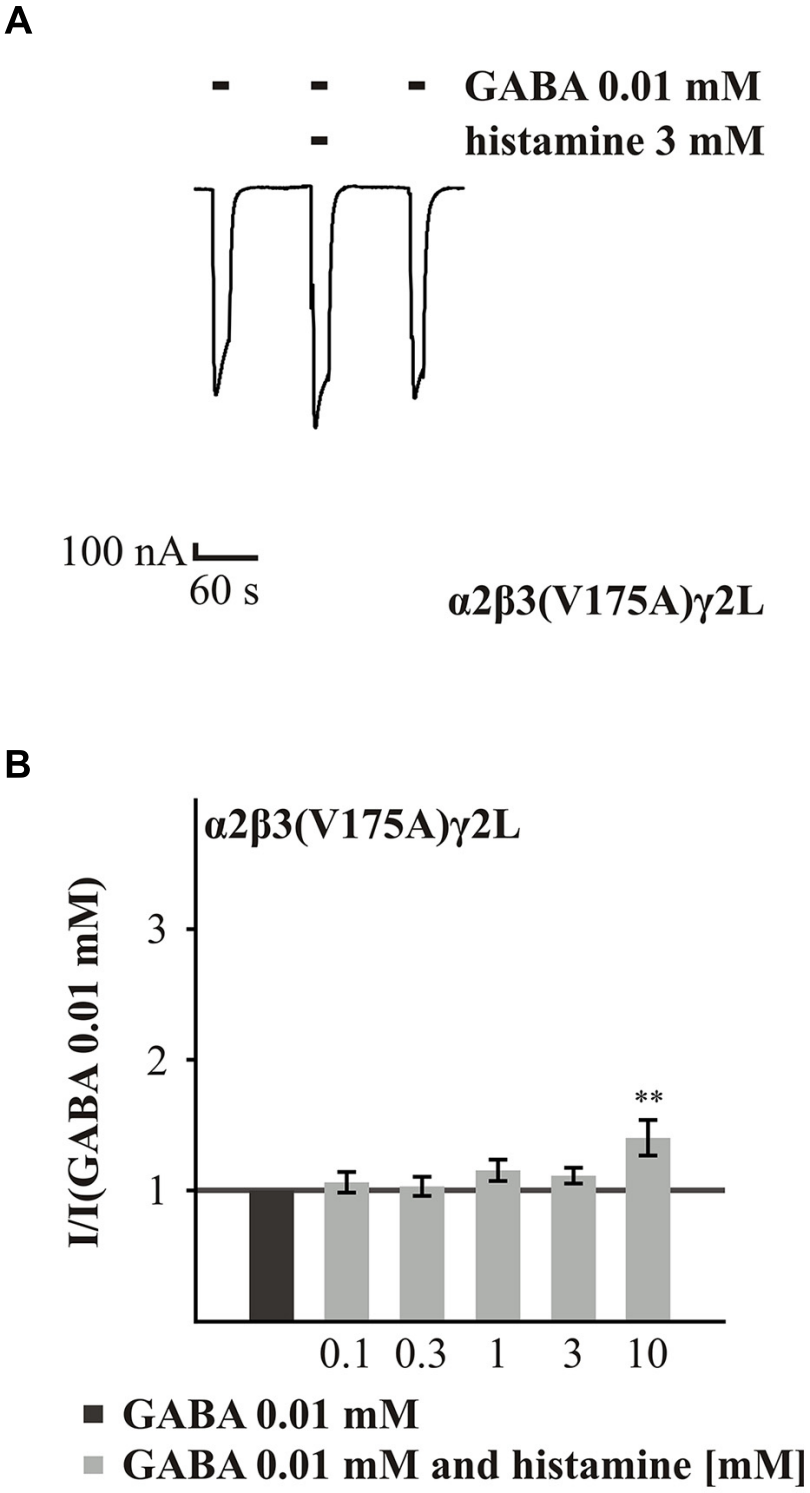
**Histamine action on the GABA-induced currents of GABA_A_Rs with point mutated β3(V175A). (A)** Original trace of the effect of 3 mM histamine on the GABA-induced currents of the GABA_A_R α2β3(V175A)γ2L. **(B)** Bar diagram show the effect of various histamine concentrations up to 10 mM on the GABA-induced currents of the GABA_A_R α2β3(V175A)γ2L. Significant data were marked with ^∗∗^*p* ≤ 0.01.

**FIGURE 6:**
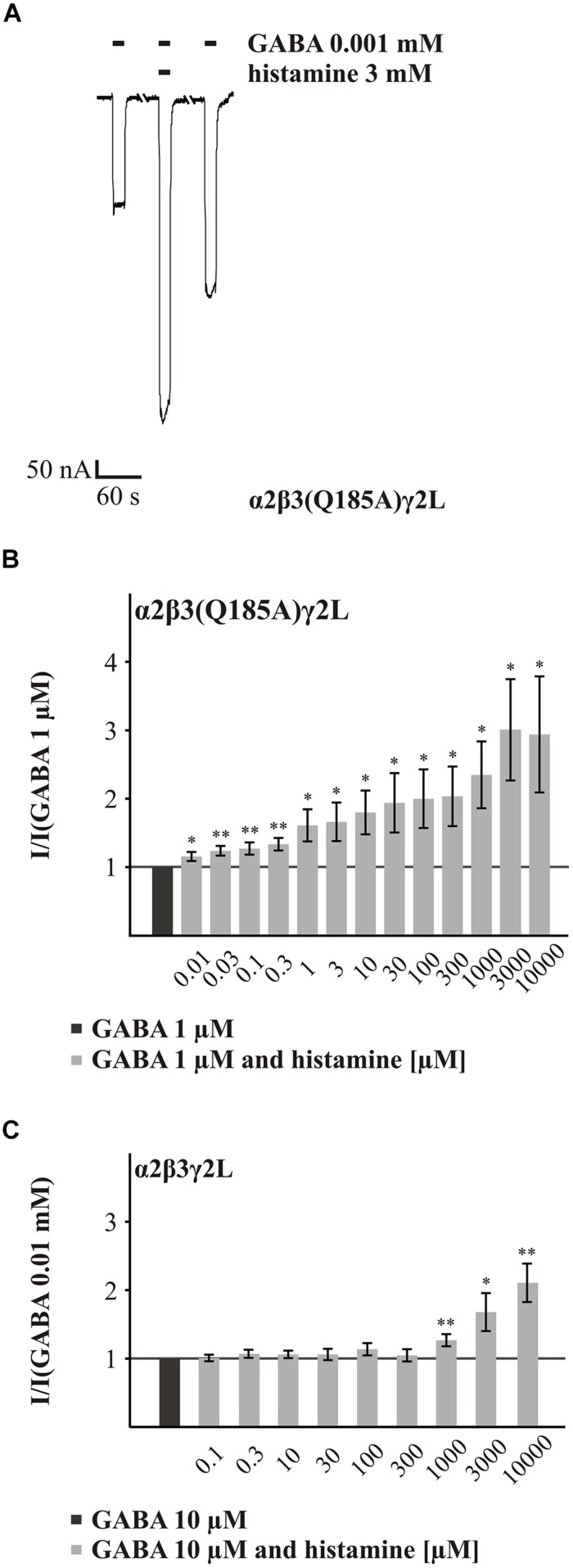
**Histamine action on the GABA-induced currents of GABA_A_Rs with the point mutated β3(Q185A) subunit. (A)** Original trace of the histamine (3 mM) action on the GABA-induced (EC_11_) currents of the GABA_A_R α2β3(Q185A)γ2L. **(B,C)** Bar diagrams that show the action of different histamine concentrations on the GABA-induced currents of the GABA_A_Rs α2β3(Q185A)γ2L and α2β3γ2L. Significant data were marked with ^∗^*p* ≤ 0.05 and ^∗∗^*p* ≤ 0.01.

The last two amino acids that we analyzed were V175 and Q185 on the β subunit. These amino acids are located in loop 9, which is in close proximity to the salt-bridge formed by α1(R120A) and β2(D163A). The experiments with the GABA_A_R α2β3(V175A)γ2L showed that the GABA-induced current was not potentiated by histamine concentrations up to 3 mM (0.1 to 3 mM; **Figure 5**). Only the highest concentration, 10 mM, significantly changed the GABA-induced (EC_16_) current, increasing it 1.4-fold (**Figure 5B**). In comparison to the wt (**Figure 6C**), the potentiation effected by histamine is drastically reduced. This suggests that V175 is involved in the action of histamine on the GABA_A_Rs. To demonstrate that this effect is not dependent on the presence of the α2 subunit, we tested the α1β3(V175A)γ2L combination and also found a significant reduction in potentiation (Supplementary Figure S1). To ensure that the loss of potentiation is specific for histamine, we tested this mutated receptor with 10 μM propofol, which caused a 1.8-fold potentiation (data not show) demonstrating that GABA-induced currents at this receptor can be potentiated by other modulators.

In the initial screening, the potentiating effect of histamine appeared to be increased at GABA_A_R α2β3(Q185A)γ2L. The investigation of various histamine concentrations (0.01 to 10 mM) revealed a dose-dependent potentiation of the GABA (EC_11_) response (**Figures 6A,B**). In comparison to the wt receptor (**Figure 6C**), where the first significant potentiation was observed at 1 μM histamine, concentrations as low as 10 μM histamine produced significant potentiation at the α2β3(Q185A)γ2L receptor. For most concentrations up to 1 mM, histamine is a significantly better potentiator at the mutated receptor (Supplementary Table S2). At higher concentrations, the effect is no longer significant, which is an indication that the potency but not the efficacy of histamine is enhanced by this mutation. To demonstrate that this effect is not dependent on the presence of the α2 subunit, we tested the α1β3(Q185A)γ2L subunit combination and also found a significant increase in potentiation (Supplementary Figure S1).

## Discussion

Our study confirms the recent finding (Saras et al., 2008; Bianchi et al., 2011) that there is a robust modulatory effect of histamine on GABA_A_Rs with different subunit combinations. The screening of point mutated GABA_A_R subunits revealed that most mutations, which encompass several amino acids conserved in β subunits, did not influence histamine potentiation. Our results demonstrate that the point mutations N265M and M286W of amino acids in the β subunit that are important for the modulation by etomidate and propofol (Siegwart et al., 2002; Jurd et al., 2003; Reynolds et al., 2003) had no effect on the modulation of the GABA-induced current by histamine. Our results indicate that histamine does not act through these known modulation mechanisms.

**FIGURE 7:**
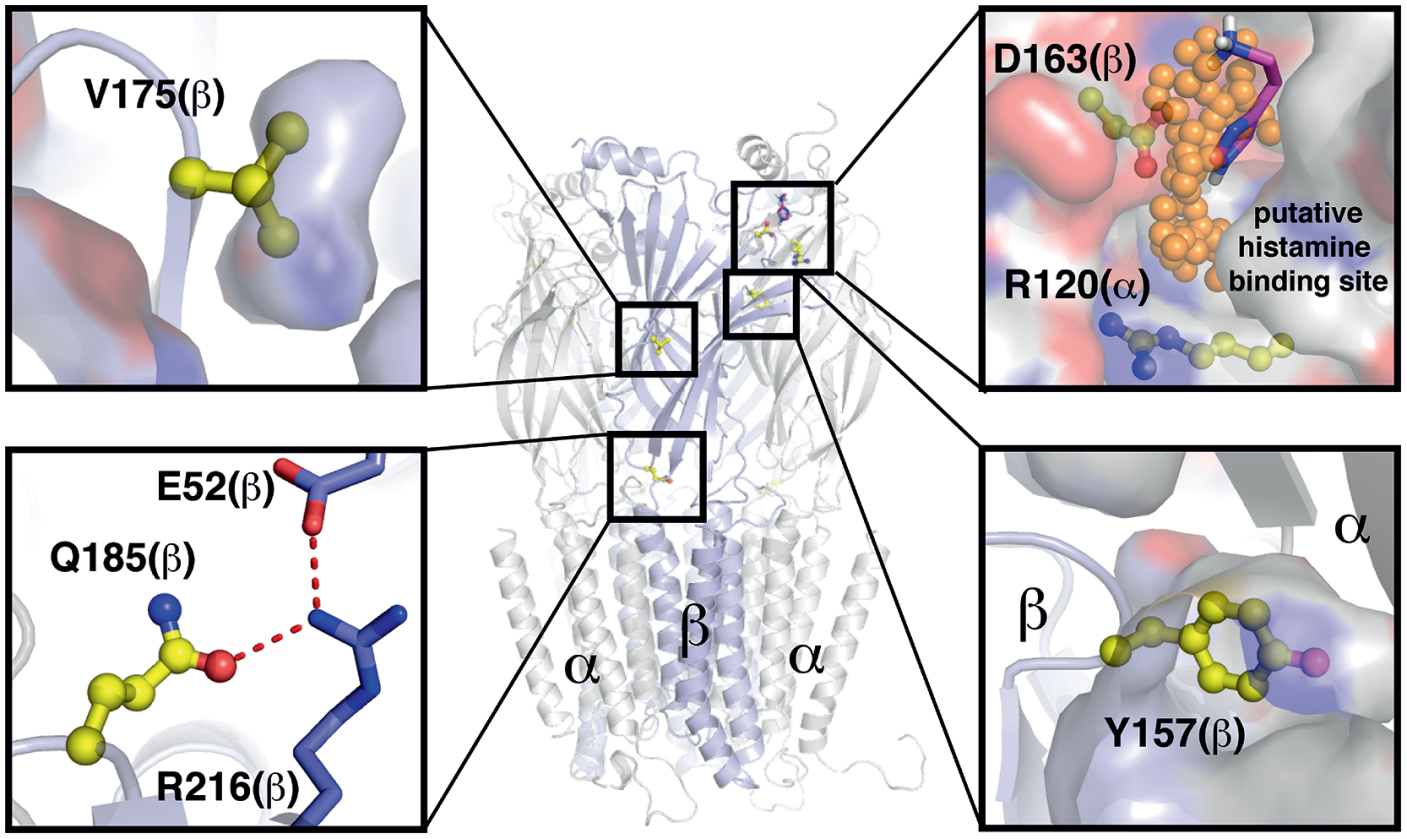
**Overview of positions and functions of mutated residues**. Structural model based on PDB ID 4COF (Miller and Aricescu, 2014). Backbone displayed as cartoon, van der Waals surface as surface, selected residues as sticks, or balls and sticks, respectively. Extracellular domains visible as sheets, transmembrane domains as helical domains. Putative binding positions of small organic molecules according to TRIDOCK analysis (te Heesen et al., 2007) as orange spheres and histamine in magenta. Y157 is actively taking part in formation of the interface between the two subdomains. D163(β) and R120(α) form a putative histamine binding site at the interface between subdomains α and β. Q185 at the interface between transmembrane and extracellular domains is part of a hydrogen bonding network with the highly conserved residues E52 and R216. The V175 side chain is buried within the extracellular subdomains, and thus important for their correct folding. None of the investigated residues except D163(β) and R120(α) show a putative small molecule binding site.

In our work, we identified amino acid residues important for the modulatory action of histamine on GABA_A_Rs. Four amino acid residues are located on the β subunit, consistent with the idea proposed by Saras et al. (2008) that the β subunit is involved in the action of histamine at the GABA_A_Rs. Most recently, the crystal structure of the human ß3 homopentameric receptor was resolved (Miller and Aricescu, 2014). To assess the potential function of the mutated amino acids, we build a homology model of an α1/β3 heteropentamer (**Figure 7**).

The point mutation β2(Y157F) was only effective in receptors containing the γ2L subunit and did not alter histamine’s action on receptors composed solely of a and β subunits. This indicates that histamine does not directly interact with the amino acid Y157. The presence of the γ2 subunit alters the potentiation by histamine (Saras et al., 2008) and propofol (Siegwart et al., 2002). An indirect participation could be caused by an influence of amino acid Y157 on the interaction of γ2 with other subunits in a heteromeric receptor. This is supported by the location of Y157 in the homology model, as it is actively taking part in formation of the interface between the two extracellular subdomains (**Figure 7**).

A further amino acid residue that is involved in the modulatory effect of histamine is β3(Q185). This amino acid has a potentially negative impact on histamine potentiation, and the Q185A mutation enhances the effect of lower concentrations of histamine on the GABA_A_R. Q185 at the interface between transmembrane and extracellular domains is part of a hydrogen bonding network with the highly conserved residues E52 and R216 (**Figure 7**). The amino acid V175 resides inside the extra cellular domain and is of potential structural importance (**Figure 7**). Therefore, the Q185A or V175A mutations influence the histamine potentiation possibly by allosteric effects and are not part of a binding site.

The two amino acid residues that we identified as possibly being directly involved in the action of histamine on the GABA_A_R are R120 on the α subunit and D163 on the β subunit. Amino acid R120 is conserved in all α subunits; therefore, our data could be valid for all α subunits. Laha and Wagner (2011) showed that these amino acids could be part of a state-dependent salt-bridge. This potential salt-bridge between R120 on the α subunit and D163 on the β subunit stabilizes the binding of GABA (Laha and Wagner, 2011). Disruption of the salt-bridge on the α subunit by the R120A mutation greatly increases the EC_50_ of GABA. The D163A mutation on the β subunit clearly reduces histamine potentiation, and histamine potentiation is nearly absent in receptors with the double mutation. Based on our homology model, these two amino acid residues are not close enough to form a salt bridge directly (**Figure 7**). However, the crystal structure is derived from a homopentameric receptor in a ligand bound state with the artificial agonist benzamidine. Though this benzamidine binding site is found in the vicinity of Y157 (Miller and Aricescu, 2014), it might influence the overall structure of the extracellular domain, including the position of R120. However, an analysis with TRIDOCK (te Heesen et al., 2007) revealed that a putative small molecule ligand binding site exists between D163 and R120 (**Figure 7**). Furthermore, our docking analysis revealed that histamine can bind in various ways at this site (see **Figure 7** and Supplementary Figure 2) with a comparably low predicted binding affinity [ΔG(bind) = -3.9 to –3.7 kcal/mol], which is in agreement with our comparably low histamine affinities measured experimentally. At the found docking positions, the histamine ammonium headgroup interacts majorly with D163(β), and thus probably weakens the R120/D163 interaction, which is in agreement with our experimental results. Furthermore, the docking positions found are in good agreement with the binding positions found by TRIDOCK. This position therefore could be a binding site for histamine, as well.

On the basis of our investigations, we suggest that histamine interacts with D163 on the β subunit and weakens the interaction of D163 with R120, which could lead to enhanced or prolonged GABA binding, a left shift of the GABA EC_50_ and thereby to potentiation. Consequently, we propose that the amino acid D163 is essential for the modulation of GABA_A_Rs by histamine and possibly a part of the histamine binding site. We demonstrate with our results a further function of the interaction of the α and β subunit mediated by D163 with R120, which is vital for both histamine potentiation and GABA affinity. The amino acid D163 is conserved in all β subunits of the GABA_A_Rs. In addition, amino acids homologous to D163 could be detected in the α1 subunit of the GlyR and α7 subunit of the nAChRs (Galzi et al., 1996; Newell et al., 2004). The homologous amino acid in the nAChR is involved in the change of the affinity for calcium and acetylcholine (Galzi et al., 1996).

## Conclusion

Our data show that histamine’s potentiation depends on amino acid D163 of the β subunit and R120 of the α subunit. Interaction of these amino acids lowers the EC_50_ for GABA (Laha and Wagner, 2011). We propose that histamine potentiates GABA_A_Rs by influencing this interaction and that these amino acids are part of a potential histamine binding site.

## Conflict of Interest Statement

The authors declare that the research was conducted in the absence of any commercial or financial relationships that could be construed as a potential conflict of interest.
