# Supplementary material for: Identification of amino acids involved in histamine potentiation of GABAA receptors
Source: Front Pharmacol. 2015 May 26;6:106. doi: 10.3389/fphar.2015.00106 (PMC4443022; doi:10.3389/fphar.2015.00106)
Supplement: Supplementary file 1 [file Data_Sheet_1.DOCX]

**Supplementary Material**


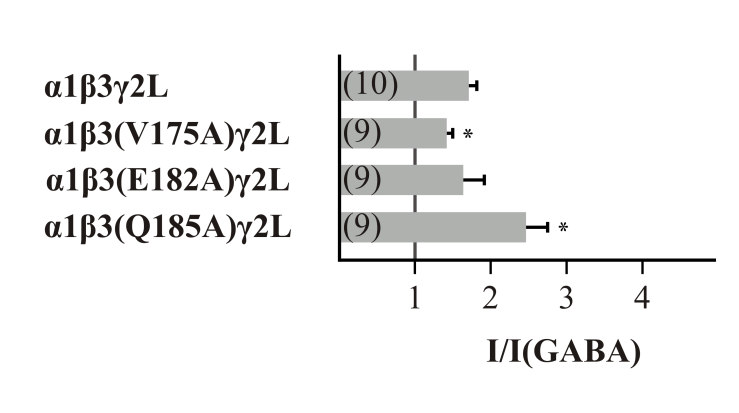


**Figure 1 Effect of 3 mM histamine on the GABA-induced currents of wt or mutated GABA_A_Rs.** The bars show the increase of the GABA-induced (typically EC_10-30_) currents by 3 mM histamine for several point mutations in comparison to wt α1β3γ2L.


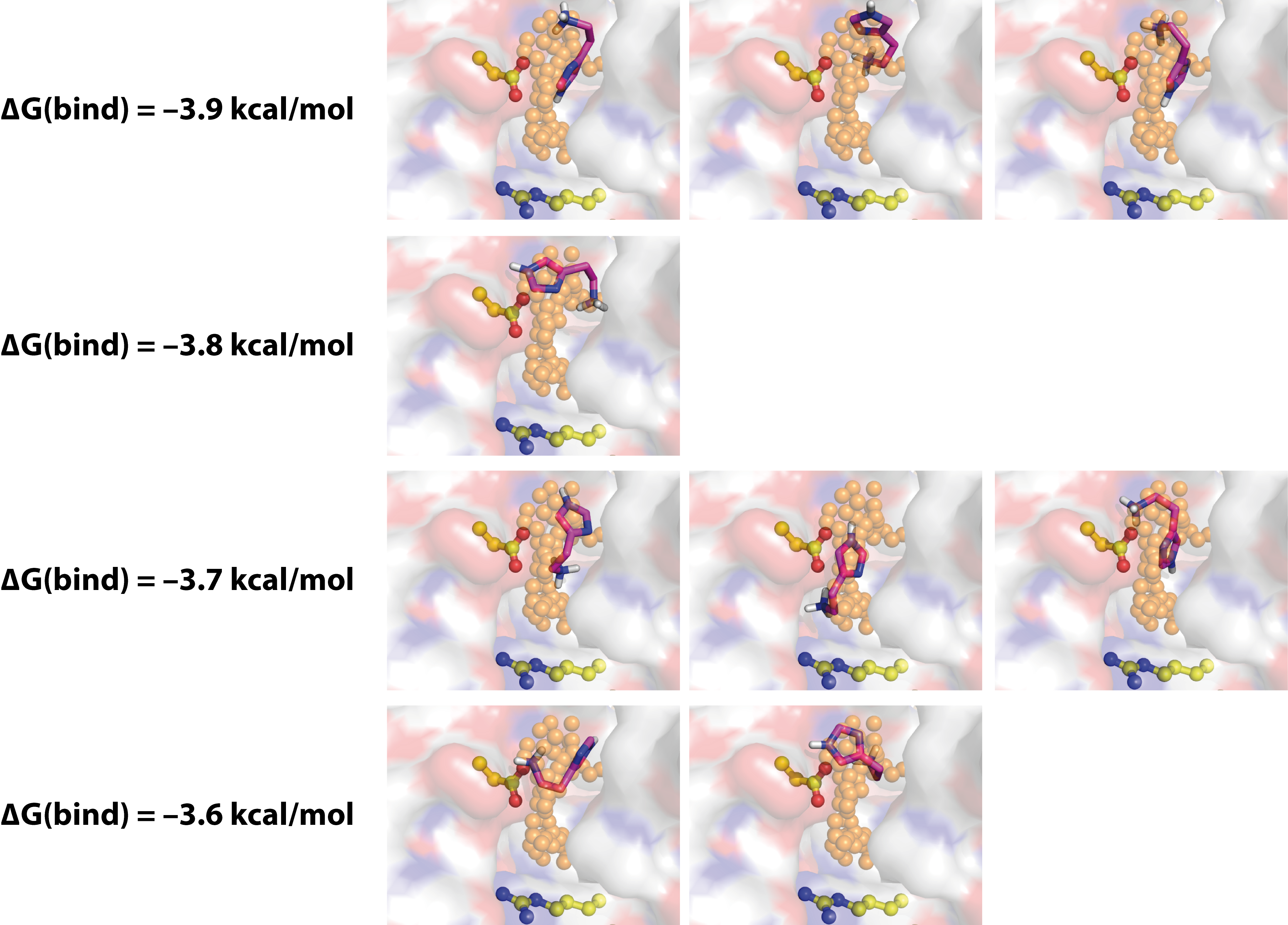


**Figure 2 Binding positions of histamine close to R120(α) and D163(β).** Van der Waals surface displayed as surface, selected residues as sticks (histamine) or balls and sticks (R120, D163), respectively. Putative binding positions of small organic molecules according to TRIDOCK analysis (te Heesen, 2007) as orange spheres. Histamine in magenta. Histamine can bind to the binding site predicted by TRIDOCK in various ways, which are energetically very similar.

**Table 1** Measured EC_50_-values of GABA for the GABA_A_Rs without or with point mutation(s). Some EC_50_-values are estimated because the high EC_50_ for these mutations precluded attaining saturating concentrations.

|  | **GABA** | | **oocytes** |
| --- | --- | --- | --- |
| **GABA_A_R** | **EC_50_ [µM]** | **nH** | **n** |
| **α1β2γ2L** | **090.22 ± 4.98** | **0.91 ± 0.04** | **12** |
| α1β2(N265M)γ2L | 048.60 ± 6.03 | 0.93 ± 0.09 | 9 |
| α1β2(M286W)γ2L | 013.26 ± 1.61 | 0.83 ± 0.07 | 9 |
| α1(F64L)β2γ2L | ≈ 1543 ± 70 | 1.16 ± 0.04 | 9 |
| α1β2(Y157A)γ2L | ≈ 1236 ± 103 | 1.17 ± 0.08 | 9 |
| α1(N185L)β2γ2L | 008.14 ± 0.47 | 1.05 ± 0.05 | 9 |
| α1(L263S)β2γ2L | 000.82 ± 0.10 | 0.57 ± 0.03 | 9 |
| α1β2(L259S)γ2L | 002.13 ± 1.10 | 0.48 ± 0.08 | 9 |
| α1(L263S)β2(L259S)γ2L | 000.41 ± 0.12 | 0.46 ± 0.05 | 9 |
| α1(R120A)β2γ2L | ≈ 1667 ± 45 | 1.04 ± 0.02 | 9 |
| α1β2(D163A)γ2L | 426.78 ± 42.69 | 1.13 ± 42.69 | 9 |
| α1(R120A)β2(D163A)γ2L | ≈ 1896 ± 13 | 1.55 ± 0.01 | 12 |
|  |  |  |  |
| **α1β3γ2L** | **030.57 ± 2.30** | **0.98 ± 0.06** | **10** |
| α1β3(H267A)γ2L | 017.88± 1.03 | 1.43 ± 0.10 | 9 |
| α1β3(E182A)γ2L | 020.52 ± 2.35 | 0.87 ± 0.07 | 9 |
| α1β3(V175A)γ2L | 042.39 ± 1.84 | 1.33 ± 0.06 | 9 |
| α1β3(Q185A)γ2L | 005.44 ± 0.25 | 1.15 ± 0.05 | 9 |
|  |  |  |  |
| **α2β3γ2L** | **035.67 ± 2.24** | **1.39 ± 0.10** | **12** |
| α2β3(E182A)γ2L | 022.90 ± 3.48 | 0.79 ± 0.08 | 9 |
| α2β3(V175A)γ2L | 040.47 ± 6.53 | 0.96 ± 0.12 | 9 |
| α2β3(Q185A)γ2L | 005.03 ± 0.19 | 1.23 ± 0.05 | 9 |
|  |  |  |  |
| **α1β2** | **013.26 ± 1.77** | **0.88 ± 0.09** | **11** |
| α1β2(Y157A) | 253.04 ± 17.75 | 1.12 ± 0.07 | 9 |

**Table 2** Comparison of the histamine potentiation of wt with point mutated receptors. Significance for the potentiation was calculated for various concentrations of histamine.

**GABA_A_R α1β2(Y157A)γ2L**

| **histamine concentration [µM]** | | **p-value** |  |
| --- | --- | --- | --- |
| 100 |  | 0.22657 | - |
| 300 |  | 0.18988 | n.s. |
| 1000 |  | 0.03161 | * |
| 3000 |  | 0.00879 | ** |
| 10000 |  | 0.00183 | ** |

**GABA_A_R α1β2(Y157A)**

| **histamine concentration [µM]** | | **p-value** |  |
| --- | --- | --- | --- |
| 100 |  | 0.1664 | n.s. |
| 300 |  | 0.4552 | n.s. |
| 1000 |  | 0.0868 | n.s. |
| 3000 |  | 0.1733 | n.s. |
| 10000 |  | 0.2749 | n.s. |

**GABA_A_R α1(R120A)β2γ2L**

| **histamine concentration [µM]** | | **p-value** |  |
| --- | --- | --- | --- |
| 100 |  | 0.2982 | n.s. |
| 300 |  | 0.4058 | n.s. |
| 1000 |  | 0.3365 | n.s. |
| 3000 |  | 0.2344 | n.s. |
| 10000 |  | 0.2834 | n.s. |

**GABA_A_R α1β2(D163A)γ2L**

| **histamine concentration [µM]** | | **p-value** |  |
| --- | --- | --- | --- |
| 100 |  | 0.0560 | n.s. |
| 300 |  | 0.1062 | n.s. |
| 1000 |  | 0.0235 | * |
| 3000 |  | 0.0246 | * |
| 10000 |  | 0.0040 | ** |

**GABA_A_R α1(R120A)β2(D163A)γ2L**

| **histamine concentration [µM]** | | **p-value** |  |
| --- | --- | --- | --- |
| 100 |  | 0.0123 | * |
| 300 |  | 0.0230 | * |
| 1000 |  | 0.0094 | ** |
| 3000 |  | 0.0103 | * |
| 10000 |  | 0.0052 | ** |

**GABA_A_R α2β3(V175A)γ2L**

| **histamine concentration [µM]** | | **p-value** |  |
| --- | --- | --- | --- |
| 100 |  | 0.4551 | n.s. |
| 300 |  | 0.3238 | n.s. |
| 1000 |  | 0.4356 | n.s. |
| 3000 |  | 0.0380 | * |
| 10000 |  | 0.0919 | n.s. |

**GABA_A_R α2β3(Q185A)γ2L**

| **histamine concentration [µM]** | | **p-value** |  |
| --- | --- | --- | --- |
| 1 |  | 0.0180 | * |
| 3 |  | 0.0470 | * |
| 10 |  | 0.0282 | * |
| 30 |  | 0.0568 | n.s. |
| 100 |  | 0.0478 | * |
| 300 |  | 0.0343 | * |
| 1000 |  | 0.0282 | * |
| 3000 |  | 0.0870 | n.s. |
| 10000 |  | 0.1549 | n.s. |

**Supplementary Table 3**

The primers used for site-directed mutagenesis were 20-46 nucleotides long. All of the primers were synthesized by Eurofins:

1. 5´-TAATACGACTCACTATAGGG-3´, T7-fwd; fwd flanking primer;

2. 5´-AGTGTAAGTTGGTATTATGTAGC-3´, pSGEM-rv; rv flanking primer;

3. 5´-GGAATATACAATAGATGTG**TTG**TTCCGCCAAAGCTGG-3´, fwd primer for point mutation F64L of the GABA_A_R α1 subunit;

4. 5´-CCAGCTTTGGCGGAA**CAA**CACATCTATTGTATATTCC-3´, rv primer for point mutation F64L of the GABA_A_R α1 subunit;

5. 5´-CAGAAGATGGGTCACGTTTA**CTT**CAGTATGACCTTCTTGGGCAAAC-3´, fwd primer for point mutation N185L of the GABA_A_R α1 subunit;

6. 5´-GTTTGCCCAAGAAGGTCATACTG**AAG**TAAACGTGACCCATCTTCTG-3´, rv primer for point mutation N185L of the GABA_A_R α1 subunit;

7. 5´-GGAGTGACGACCGTT**AGC**ACCATGACAACC-3´, fwd primer for point mutation L263S of the GABA_A_R α1 subunit;

8. 5´-GGTTGTCATGGT**GCT**AACGGTCGTCACTCC-3´, rv primer for point mutation L263S of the GABA_A_R α1 subunit;

9. 5´-GGAATTACAACTGTC**AGC**ACGATGACCAC-3´, fwd primer for point mutation L259S of the GABA_A_R β2 subunit;

10. 5´-GTGGTCATCGT**GCT**GACAGTTGTAATTCC-3´, rv primer for point mutation L259S of the GABA_A_R β2 subunit;

11. 5´-GCCCAATAAACTCCTG**GCG**ATCACAGAGGATGGC-3´, fwd primer for point mutation R120A of the GABA_A_R α1 subunit;

12. 5´-GCCATCCTCTGTGAT**CGC**CAGGAGTTTATTGGGC-3´, rv primer for point mutation R120A of the GABA_A_R α1 subunit;

13. 5´-GGCTATACAACTGAT**GCG**ATTGAGTTTTACTGGC-3´, fwd primer for point mutation D163A of the GABA_A_R β2 subunit;

14. 5´-GCCAGTAAAACTCAAT**CGC**ATCAGTTGTATAGCC-3´, rv primer for point mutation D163A of the GABA_A_R β2 subunit;

15. 5´-GTGGAAAGGATT**GCG**CTCCCGCAGTTCTCCATCGTGG-3´, fwd primer for point mutation E182A of the GABA_A_R β3 subunit;

16. 5´-GAGAACTGCGGGAG**CGC**AATCCTTTCCACTCCGGTAAC-3´, rv primer for point mutation E182A of the GABA_A_R β3 subunit;

17. 5´-CGGGGACAAGGCT**GCG**ACCGGAGTGGAAAGG-3´, fwd primer for point mutation V175A of the GABA_A_R β3 subunit;

18. 5´-CCTTTCCACTCCGGT**CGC**AGCCTTGTCCCCG-3´, rv primer for point mutation V175A of the GABA_A_R β3 subunit;

19. 5´-GGATTGAGCTCCCG**GCG**TTCTCCATCGTGGAGC-3´, fwd primer for point mutation Q185A of the GABA_A_R β3 subunit;

20. 5´-GCTCCACGATGGAGAA**CGC**CGGGAGCTCAATCC-3´, rv primer for point mutation Q185A of the GABA_A_R β3 subunit;

21. 5´-CGTTGGAGATCGAAAGC**TTC**GGCTATACAACTG-3´, fwd primer for point mutation Y157F of the GABA_A_R β2 subunit and

22. 5´-CAGTTGTATAGCC**GAA**GCTTTCGATCTCCAACG-3´, rv primer for point mutation Y157F of the GABA_A_R β2 subunit.

**Dataset 1** alignment of α1 and β3 subunit sequences by MUSCLE.

Score = 5960

Length of alignment = 365

Sequence a1 : 256 - 684 (Sequence length = 429)

Sequence b3 : 247 - 601 (Sequence length = 355)

a1 QPSLQDELKDNTTVFTRILDRLLDGYDNRLRPGLGERVTEVKTDIFVTSFGPVSDHDMEYTIDVFFRQS

|..| .| . .|.|| ||| ||||..| | .| ..|.. ||. .|.||. ..|.|

b3 GQSVND--PGNMSFVKETVDKLLKGYDIRLRPDFGGPPVCVGMNIDIASIDMVSEVNMDYTLTMYFQQY

a1 WKDERLKFKGPMTVLRLNNLMASKIWTPDTFFHNGKKSVAHNMTMPNKLLRITEDGTLLYTMRLTVRAE

|.| || . | | |.| .| ..| |||.| |.||| | .|. |...|. |||.|| .|.| |

b3 WRDKRLAYSGIPLNLTLDNRVADQLWVPDTYFLNDKKSFVHGVTVKNRMIRLHPDGTVLYGLRITTTAA

a1 CPMHLEDFPMDAHACPLKFGSYAYTRAEVVYEWTREPARSVVVAEDGSRLNQYDLLGQTVDSGIVQSST

| |.| .|.| . | | . ||.|| .. . | . | . . | |. .. . . | | .|

b3 CMMDLRRYPLDEQNCTLEIESYGYTTDDIEFYW--RGGDKAVTGVERIELPQFSIVEHRLVSRNVVFAT

a1 GEYVVMTTHFHLKRKIGYFVIQTYLPCIMTVILSQVSFWLNRESVPARTVFGVTTVLTMTTLSISARNS

| | .. |.|||.||||..|||.| |. ||| ||||.| .. .|| .|.||||||||.. |..

b3 GAYPRLSLSFRLKRNIGYFILQTYMPSILITILSWVSFWINYDASAARVALGITTVLTMTTINTHLRET

a1 LPKVAYATAMDWFIAVCYAFVFSALIEFATVNYFTKRGYAWDGKSVVPEKPKKVKDPLIKKNNTYAPTA

|||..| |.| .. |. ||| ||.|.| |||. .. ... .. .. |. |..

b3 LPKIPYVKAIDMYLMGCFVFVFLALLEYAFVNYIF---FSQPARAAAIDRWSRIVFPF-----TFSLFN

a1 TSYTPNLARGDPGLATIAKS

| | . .| .

b3 LVYWLYYVNGATETSQVAPA

Percentage ID = 35.34

Percentage similarity = 61.92

**Dataset 2** WHATIF check of the α1 / β3 heteropentamer homology model structure. Subunits A and C are α1 subunits, B, D and E are β3 subunits.

************************************************************************

********** REPORT OF PROTEIN ANALYSIS by the WHAT IF program **********

************************************************************************

Date : 2015-04-21

This report was created by WHAT IF version 20150310-1640

This document is a WHAT_CHECK-report that holds the findings of the WHAT IF

program during the analysis of a PDB-file. Each reported fact has an assigned

severity, one of:

error : Items marked as errors are considered severe problems requiring

immediate attention.

warning: Either less severe problems or uncommon structural features. These

still need special attention.

note : Statistical values, plots, or other verbose results of tests and

analyses that have been performed.

If alternate conformations are present, only the first is evaluated. Hydrogen

atoms are only included if explicitly requested, and even then they are not

used in all checks. The software functions less well for non-canonical amino

acids and exotic ligands than for the 20 canonical residues and canonical

nucleic acids.

Some remarks regarding the output:

Residues/atoms in tables are normally given in a few parts:

A number. This is the internal sequence number of the residue used by WHAT IF.

The first residues in the file get number 1, 2, etc.

The residue type. Normally this is a three letter amino acid type.

The sequence number, between brackets. This is the residue number as it was

given in the input file. It can be followed by the insertion code.

The chain identifier. A single character. If no chain identifier was given in

the input file, this will be a minus sign or a blank.

A model number. If no model number exists, like in most X-ray files, this will

be a blank or occasionally a minus sign.

In case an atom is part of the output, the atom will be listed using the PDB

nomenclature for type and identifier.

To indicate the normality of a score, the score may be expressed as a Z-value

or Z-score. This is just the number of standard deviations that the score

deviates from the expected value. A property of Z-values is that the

root-mean-square of a group of Z-values (the RMS Z-value) is expected to be

1.0. Z-values above 4.0 and below -4.0 are very uncommon. If a Z-score is

used in WHAT IF, the accompanying text will explain how the expected value

and standard deviation were obtained.

The names of nucleic acids are DGUA, DTHY, OCYT, OADE, etc. The first character

is a D or O for DNA or RNA respectively. This circumvents ambiguities in the

many old PDB files in which DNA and RNA were both called A, C, G, and T.

# 1 # Note: Introduction

WHAT CHECK needs to read in a PDB file before it can check it. It does a

series of checks upon reading the file. The results of these checks are

reported in this section (section 2.1). The rest of the report will be more

systematic in that section 2.2 reports on administrative problems. Section

2.3 gives descriptive output that is not directly validating things but

more telling you how WHAT CHECK interpreted the input file. Section 2.4

looks at B-factors, occupancies, and the presence/absence of (spurious)

atoms. Section 2.5 deals with nomenclature problems. Section 2.6 deals with

geometric problems like bond lengths and bond angles. Section 2.7 deals with

torsion angle issues. Section 2.8 looks at atomic clashes. Section 2.9 deals

with packing, accessibility, etc, issues. Section 2.10 deals with hydrogen

bonds, ion packing, and other things that can be summarized under the common

name charge-charge interactions. Section 2.11 gives a summary of whole report

and tells you (if applicable) which syyetry matrices were used. Section 2.12

tells the crystallographer which are the things most in need of manual

correction. And the last section, section 2.13, lists all residues sorted

by their need for visual inspection.

Entering CANONI for: /hom

# 2 # Error: Missing unit cell information

No SCALE matrix is given in the PDB file.

# 3 # Error: Missing symmetry information

Problem: No CRYST1 card is given in the PDB file.

SYMMETRY will be unavailable for this molecule.

# 4 # Note: No strange inter-chain connections detected

No covalent bonds have been detected between molecules with non-identical

chain identifiers.

# 5 # Note: No duplicate atom names in ligands

All atom names in ligands (if any) seem adequately unique.

# 6 # Note: In all cases the primary alternate atom was used

WHAT CHECK saw no need to make any alternate atom corrections (which means

they either are all correct, or there are none).

# 7 # Note: No residues detected inside ligands

Either this structure does not contain ligands with amino acid groups inside

it, or their naming is proper (enough).

# 8 # Note: No attached groups interfere with hydrogen bond calculations

It seems there are no sugars, lipids, etc., bound (or very close) to atoms

that otherwise could form hydrogen bonds.

# 9 # Note: No probable side chain atoms with zero occupancy detected.

Either there are no side chain atoms with zero occupancy, or the side chain

atoms with zero occupancy were not present in the input PDB file (in which

case they are listed as missing atoms), or their positions are sufficiently

improbable to warrant a zero occupancy.

# 10 # Note: No probable backbone atoms with zero occupancy detected.

Either there are no backbone atoms with zero occupancy, or the backbone

atoms with zero occupancy were left out of the input PDB file (in

which case they are listed as missing atoms), or their positions are

sufficiently improbable to warrant a zero occupancy.

# 11 # Note: All residues have a complete backbone.

No residues have missing backbone atoms.

# 12 # Note: No C-alpha only residues

There are no residues that consist of only an alpha carbon atom.

# 13 # Note: Non-canonical residues

WHAT CHECK has not detected any non-canonical residue(s).

# 14 # Note: Content of the PDB file as interpreted by WHAT CHECK

Content of the PDB file as interpreted by WHAT CHECK.

WHAT CHECK has read your PDB file, and stored it internally in what is called

'the soup'. The content of this soup is listed here. An extensive explanation

of all frequently used WHAT CHECK output formats can be found at

swift.cmbi.ru.nl. Look under output formats. A course on reading this

'Molecules' table is part of the WHAT CHECK website.

1 1 ( 4) 413 ( 416) A Protein checkset

2 414 ( 13) 817 ( 416) C Protein checkset

\begin{note}

\showsect{Some numbers...}

Number of NMR MODELs skipped upon reading PDB file: 0

This is an X-ray structure. No MODELs found

The total number of amino acids found is 817.

No nucleic acids observed in input file

No sugars recognized in input file

No water observed in input file

Residue numbers increase monotonously OK

\end{note}

Content of the SOUP. See the writeup for an explanation.

Molecule Range Type Set name

1 1 ( 4) 413 ( 416)A Protein checkset

2 414 ( 13) 817 ( 416)C Protein checkset

# 15 # Note: Ramachandran plot

In this Ramachandran plot x-signs represent glycines, squares represent

prolines, and plus-signs represent the other residues. If too many

plus-signs fall outside the contoured areas then the molecule is poorly

refined (or worse). Proline can only occur in the narrow region around

phi=-60 that also falls within the other contour islands.

In a colour picture, the residues that are part of a helix are shown in blue,

strand residues in red. Preferred regions for helical residues are drawn in

blue, for strand residues in red, and for all other residues in green. A full

explanation of the Ramachandran plot together with a series of examples can

be found at the WHAT CHECK website [REF].

In the TeX file, a plot has been inserted here

Chain identifier: A

# 16 # Note: Ramachandran plot

In the TeX file, a plot has been inserted here

Chain identifier: C

# 17 # Note: Secondary structure

This is the secondary structure according to DSSP. Only helix (H), overwound

or 3/10-helix (3), strand (S), turn (T) and coil (blank) are shown [REF].

All DSSP related information can be found at swift.cmbi.ru.nl/gv/dssp/

This is not really a structure validation option, but a very scattered

secondary structure (i.e. many strands of only a few residues length, many

Ts inside helices, etc) tends to indicate a poor structure. A full

explanation of the DSSP secondary structure determination program together

with a series of examples can be found at the WHAT CHECK website [REF].

Secondary structure assignment

10 20 30 40 50 60

| | | | | |

1 - 60 LQDELKDNTTVFTRILDRLLDGYDNRLRPGLGERVTEVKTDIFVTSFGPVSDHDMEYTID

( 4)-( 63) TT HHHHHHHHHHHHTT TT TTTT SSSSSSSSSSSSSSSSTTTTSSSSS

70 80 90 100 110 120

| | | | | |

61 - 120 VFFRQSWKDERLKFKGPMTVLRLNNLMASKIWTPDTFFHNGKKSVAHNMTMPNKLLRITE

( 64)-( 123)SSSSSSSS HHH SSS HHHHHH SSSTTSSSSSS SSSSS T

130 140 150 160 170 180

| | | | | |

121 - 180 DGTLLYTMRLTVRAECPMHLEDFPMDAHACPLKFGSYAYTRAEVVYEWTREPARSVVVAE

( 124)-( 183)T SSSSSSSSSSSSS TTTT SSSSSSSSSS TTTSSSSS HHHHSS T

190 200 210 220 230 240

| | | | | |

181 - 240 DGSRLNQYDLLGQTVDSGIVQSSTGEYVVMTTHFHLKRKIGYFVIQTYLPCIMTVILSQV

( 184)-( 243)TT SSSSSSSSSSSSSS SSSSSSSSSSSSS THHHHHHTHHHHHHHHHHHHH

250 260 270 280 290 300

| | | | | |

241 - 300 SFWLNRESVPARTVFGVTTVLTMTTLSISARNSLPKVAYATAMDWFIAVCYAFVFSALIE

( 244)-( 303)HHH TT HHHHHHHHHHHHHHHHHHHHHHHHH HHHHHHHHHHHHHHHHHHH

310 320 330 340 350 360

| | | | | |

301 - 360 FATVNYFTKRGYAWDGKSVVPEKPKKVKDPLIKKNNTYAPTATSYTPNLARGDPGLATIA

( 304)-( 363)HHHHTT HHHH TT

370 380 390 400 410

| | | | |

361 - 413 KSATIEPKEVKPETKPPEPKKTFNSVSKIDRLSRIAFPLLFGIFNLVYWATYL

( 364)-( 416) HHHHHHHHHHHHHHHHHHHHHHHHHH

420 430 440 450 460 470

| | | | | |

414 - 473 TVFTRILDRLLDGYDNRLRPGLGERVTEVKTDIFVTSFGPVSDHDMEYTIDVFFRQSWKD

( 13)-( 72) HHHHHHHHHHTT TT TTTT SSSSSSSSSSSSSSSSTTTTSSSSSSSSSSSSS

480 490 500 510 520 530

| | | | | |

474 - 533 ERLKFKGPMTVLRLNNLMASKIWTPDTFFHNGKKSVAHNMTMPNKLLRITEDGTLLYTMR

( 73)-( 132)HHH SSS HHHHHH SSSTTSSSSSS SSSSS TT SSSSSSS

540 550 560 570 580 590

| | | | | |

534 - 593 LTVRAECPMHLEDFPMDAHACPLKFGSYAYTRAEVVYEWTREPARSVVVAEDGSRLNQYD

( 133)-( 192)SSSSSS TTTT SSSSSSSSSS TTTSSSS HHHSS TTT SS

600 610 620 630 640 650

| | | | | |

594 - 653 LLGQTVDSGIVQSSTGEYVVMTTHFHLKRKIGYFVIQTYLPCIMTVILSQVSFWLNRESV

( 193)-( 252)SSSSSSSSSSSS SSSSSSSSSSSSS THHHHHHTHHHHHHHHHHHHHHHH TT H

660 670 680 690 700 710

| | | | | |

654 - 713 PARTVFGVTTVLTMTTLSISARNSLPKVAYATAMDWFIAVCYAFVFSALIEFATVNYFTK

( 253)-( 312)HHHHHHHHHHHHHHHHHHHHHHHT HHHHHHHHHHHHHHHHHHHHHHHHT

720 730 740 750 760 770

| | | | | |

714 - 773 RGYAWDGKSVVPEKPKKVKDPLIKKNNTYAPTATSYTPNLARGDPGLATIAKSATIEPKE

( 313)-( 372) TT HHHH TTT

780 790 800 810

| | | |

774 - 817 VKPETKPPEPKKTFNSVSKIDRLSRIAFPLLFGIFNLVYWATYL

( 373)-( 416) HHHHHHHHHHHHHHHHHHHHHHH

# 18 # Note: No rounded coordinates detected

No significant rounding of atom coordinates has been detected.

# 19 # Warning: Artificial side chains detected

At least two residues (listed in the table below) were detected with chi-1

equal to 0.00 or 180.00. Since this is highly unlikely to occur accidentally,

the listed residues have probably not been refined.

23 TYR ( 26-) A -

66 SER ( 69-) A -

193 GLN ( 196-) A -

# 20 # Note: No missing atoms detected in residues

All expected atoms are present in residues. This validation option has not

looked at 'things' that can or should be attached to the elemantary building

blocks (amino acids, nucleotides). Even the C-terminal oxygens are treated

separately.

# 21 # Note: All B-factors fall in the range 0.0 - 100.0

All B-factors are larger than zero, and none are observed above 100.0.

# 22 # Note: No C-terminal nitrogen detected

The PDB indicates that a residue is not the true C-terminus by including

only the backbone N of the next residue. This has not been observed in this

PDB file.

# 23 # Note: C-terminus capping

The residues listed in the table below either are pseudo C-terminal residues,

or have two groups attached of which neither is the normal C-terminal O.

In this table REAL means that the C-terminal residue is likely to be the real

C-terminus of its chain; OX means that an incorrect second oxygen (OXT) was

detected that should not be there; -O indicates that the 'normal' oxygen

(i.e. not the OXT) is missing; OT indicates the detection of any other

capping group. C-terminal nitrogen atoms, if any, have already been dealt

with in a previous check and are indicated here by -N. PSEUDO means that

this is the last visible residue in the chain, but not the real C-terminus,

i.e. all residues after this one are missing in this chain. BREAK means that

this is the last residue before a chain-break, i.e. the chain continues

but after this residue a number of residues is missing. In case a break is

observed the number of residues that seems to be missing is shown in

brackets. OK means that given the status (REAL, PSEUDO, BREAK), no problems

were found.

Be aware that we cannot easily see the difference between these errors and

errors in the chain and residue numbering schemes. So do not blindly trust

the table below.

413 LEU ( 416-) A - : Unknown problem

817 LEU ( 416-) C - : Unknown problem

# 24 # Note: No OXT found in the middle of chains

No OXT groups were found in the middle of protein chains.

# 25 # Note: Introduction to the nomenclature section.

Nomenclature problems seem, at first, rather unimportant. After all who

cares if we call the delta atoms in leucine delta2 and delta1 rather than

the other way around. Chemically speaking that is correct. But structures

have not been solved and deposited just for chemists to look at them. Most

times a structure is used, it is by software in a bioinformatics lab. And

if they compare structures in which the one used C delta1 and delta2 and the

other uses C delta2 and delta1, then that comparison will fail. Also, we

recalculate all structures every so many years to make sure that everybody

always can get access to the best coordinates that can be obtained from

the (your?) experimental data. These recalculations will be troublesome if

there are nomenclature problems.

Several nomenclature problems actually are worse than that. At the

WHAT CHECK website [REF] you can get an overview of the importance of all

nomenclature problems that we list.

# 26 # Note: Valine nomenclature OK

No errors were detected in valine nomenclature.

# 27 # Note: Threonine nomenclature OK

No errors were detected in threonine nomenclature.

# 28 # Note: Isoleucine nomenclature OK

No errors were detected in isoleucine nomenclature.

# 29 # Note: Leucine nomenclature OK

No errors were detected in leucine nomenclature.

# 30 # Note: Arginine nomenclature OK

No errors were detected in arginine nomenclature.

# 31 # Note: Tyrosine torsion conventions OK

No errors were detected in tyrosine torsion angle conventions.

# 32 # Note: Phenylalanine torsion conventions OK

No errors were detected in phenylalanine torsion angle conventions.

# 33 # Note: Aspartic acid torsion conventions OK

No errors were detected in aspartic acid torsion angle conventions.

# 34 # Note: Glutamic acid torsion conventions OK

No errors were detected in glutamic acid torsion angle conventions.

# 35 # Note: Phosphate group names OK in DNA/RNA

No errors were detected in nucleic acid phosphate group naming conventions.

# 36 # Note: Heavy atom naming OK

No errors were detected in the atom names for non-hydrogen atoms. Please

be aware that the PDB wants us to deliberately make some nomenclature errors;

especially in non-canonical amino acids.

# 37 # Warning: Unusual bond lengths

The bond lengths listed in the table below were found to deviate more than 4

sigma from standard bond lengths (both standard values and sigmas for amino

acid residues have been taken from Engh and Huber [REF], for DNA they were

taken from Parkinson et al [REF]). In the table below for each unusual bond

the bond length and the number of standard deviations it differs from the

normal value is given.

Atom names starting with "-" belong to the previous residue in the chain. If

the second atom name is "-SG*", the disulphide bridge has a deviating length.

169 THR ( 172-) A - N -C 1.42 4.5

572 TRP ( 171-) C - CA C 1.42 -4.9

794 ASP ( 393-) C - N CA 1.38 -4.3

# 38 # Note: Normal bond length variability

Bond lengths were found to deviate normally from the standard bond lengths

(values for Protein residues were taken from Engh and Huber [REF], for

DNA/RNA from Parkinson et al [REF]).

RMS Z-score for bond lengths: 0.949

RMS-deviation in bond distances: 0.019

# 39 # Warning: Unusual bond angles

The bond angles listed in the table below were found to deviate more than 4

sigma from standard bond angles (both standard values and sigma for protein

residues have been taken from Engh and Huber [REF], for DNA/RNA from

Parkinson et al [REF]). In the table below for each strange angle the bond

angle and the number of standard deviations it differs from the standard

values is given. Please note that disulphide bridges are neglected. Atoms

starting with "-" belong to the previous residue in the sequence.

3 ASP ( 6-) A - CA CB CG 108.36 -4.2

8 ASN ( 11-) A - CA CB CG 108.45 -4.2

12 PHE ( 15-) A - CA CB CG 101.54 -12.3

16 LEU ( 19-) A - N CA CB 117.60 4.2

17 ASP ( 20-) A - CA CB CG 106.23 -6.4

41 ASP ( 44-) A - CA CB CG 108.60 -4.0

43 PHE ( 46-) A - CA CB CG 108.48 -5.3

53 HIS ( 56-) A - NE2 CD2 CG 110.56 4.1

55 MET ( 58-) A - N CA CB 117.82 4.3

58 THR ( 61-) A - N CA CB 117.70 4.2

72 LEU ( 75-) A - -C N CA 129.28 4.2

74 PHE ( 77-) A - CA CB CG 102.94 -10.9

78 MET ( 81-) A - -C N CA 129.21 4.2

90 LYS ( 93-) A - C CA CB 101.14 -4.7

95 ASP ( 98-) A - -C N CA 135.01 7.4

97 PHE ( 100-) A - CA CB CG 109.57 -4.2

98 PHE ( 101-) A - CA CB CG 105.51 -8.3

99 HIS ( 102-) A - NE2 CD2 CG 110.55 4.0

100 ASN ( 103-) A - CA CB CG 108.22 -4.4

108 ASN ( 111-) A - -C N CA 129.11 4.1

109 MET ( 112-) A - CG SD CE 91.41 -4.3

139 HIS ( 142-) A - NE2 CD2 CG 110.56 4.1

140 LEU ( 143-) A - -C N CA 132.07 5.8

142 ASP ( 145-) A - CA CB CG 108.11 -4.5

145 MET ( 148-) A - -C N CA 131.53 5.5

146 ASP ( 149-) A - C CA CB 97.18 -6.8

148 HIS ( 151-) A - NE2 CD2 CG 110.64 4.1

154 PHE ( 157-) A - C CA CB 102.39 -4.1

163 GLU ( 166-) A - CB CG CD 104.69 -4.7

168 TRP ( 171-) A - CA C O 112.36 -5.0

169 THR ( 172-) A - -C N CA 136.63 8.3

169 THR ( 172-) A - N CA CB 126.69 9.5

169 THR ( 172-) A - C CA CB 95.42 -7.7

170 ARG ( 173-) A - -CA -C N 105.94 -5.1

170 ARG ( 173-) A - -C N CA 133.76 6.7

170 ARG ( 173-) A - N CA C 97.17 -5.0

171 GLU ( 174-) A - -C N CA 144.58 12.7

176 VAL ( 179-) A - CA CB CG1 99.88 -6.2

181 ASP ( 184-) A - CA CB CG 108.60 -4.0

186 ASN ( 189-) A - CA CB CG 106.64 -6.0

189 ASP ( 192-) A - CA CB CG 108.00 -4.6

210 MET ( 213-) A - C CA CB 101.11 -4.7

210 MET ( 213-) A - CG SD CE 89.75 -5.1

215 HIS ( 218-) A - NE2 CD2 CG 110.53 4.0

221 GLY ( 224-) A - -C N CA 128.31 4.5

223 PHE ( 226-) A - CA CB CG 105.91 -7.9

226 GLN ( 229-) A - -C N CA 112.51 -5.1

226 GLN ( 229-) A - CB CG CD 100.10 -7.4

242 PHE ( 245-) A - C CA CB 100.52 -5.0

250 PRO ( 253-) A - -CA -C N 123.68 4.5

255 PHE ( 258-) A - CA CB CG 101.93 -11.9

272 ASN ( 275-) A - CA CB CG 107.89 -4.7

286 PHE ( 289-) A - CA CB CG 107.22 -6.6

293 PHE ( 296-) A - CA CB CG 109.44 -4.4

295 PHE ( 298-) A - CA CB CG 107.85 -5.9

300 GLU ( 303-) A - -C N CA 113.23 -4.7

300 GLU ( 303-) A - N CA CB 122.10 6.8

301 PHE ( 304-) A - CA CB CG 108.03 -5.8

304 VAL ( 307-) A - C CA CB 95.45 -7.7

319 VAL ( 322-) A - -C N CA 111.58 -5.6

323 LYS ( 326-) A - -C N CA 112.80 -4.9

326 LYS ( 329-) A - -C N CA 131.70 5.6

330 PRO ( 333-) A - -CA -C N 123.04 4.1

335 ASN ( 338-) A - CA CB CG 108.02 -4.6

336 ASN ( 339-) A - CA CB CG 107.98 -4.6

341 THR ( 344-) A - N CA CB 117.86 4.3

348 ASN ( 351-) A - CA CB CG 107.74 -4.9

353 ASP ( 356-) A - CA CB CG 107.70 -4.9

381 LYS ( 384-) A - -C N CA 113.62 -4.5

386 VAL ( 389-) A - C CA CB 122.75 6.7

390 ASP ( 393-) A - N CA CB 103.49 -4.1

397 PHE ( 400-) A - C CA CB 101.20 -4.7

401 PHE ( 404-) A - CA CB CG 109.79 -4.0

404 PHE ( 407-) A - CA CB CG 105.24 -8.6

412 TYR ( 415-) A - CA CB CG 104.89 -4.6

416 PHE ( 15-) C - CA CB CG 107.44 -6.4

421 ASP ( 20-) C - CA CB CG 108.37 -4.2

425 ASP ( 24-) C - CA CB CG 107.98 -4.6

428 ASP ( 27-) C - CA CB CG 107.01 -5.6

429 ASN ( 28-) C - CA CB CG 108.51 -4.1

439 VAL ( 38-) C - C CA CB 101.93 -4.3

447 PHE ( 46-) C - CA CB CG 107.66 -6.1

456 ASP ( 55-) C - CA CB CG 107.97 -4.6

457 HIS ( 56-) C - NE2 CD2 CG 110.61 4.1

458 ASP ( 57-) C - CA CB CG 107.99 -4.6

478 PHE ( 77-) C - CA CB CG 103.81 -10.0

482 MET ( 81-) C - -C N CA 128.99 4.1

499 ASP ( 98-) C - -C N CA 132.10 5.8

502 PHE ( 101-) C - CA CB CG 107.18 -6.6

503 HIS ( 102-) C - NE2 CD2 CG 110.55 4.0

504 ASN ( 103-) C - CA CB CG 108.19 -4.4

511 HIS ( 110-) C - NE2 CD2 CG 110.58 4.1

512 ASN ( 111-) C - CA CB CG 108.01 -4.6

517 ASN ( 116-) C - CA CB CG 107.51 -5.1

533 ARG ( 132-) C - N CA CB 118.47 4.7

544 LEU ( 143-) C - -C N CA 132.04 5.7

547 PHE ( 146-) C - N CA CB 118.40 4.6

549 MET ( 148-) C - -C N CA 130.26 4.8

552 HIS ( 151-) C - -C N CA 114.47 -4.0

552 HIS ( 151-) C - CA CB CG 107.90 -5.9

552 HIS ( 151-) C - NE2 CD2 CG 110.64 4.1

572 TRP ( 171-) C - C CA CB 99.74 -5.5

573 THR ( 172-) C - -C N CA 129.07 4.1

573 THR ( 172-) C - N CA C 92.30 -6.8

574 ARG ( 173-) C - -C N CA 113.33 -4.7

574 ARG ( 173-) C - N CA C 98.71 -4.5

576 PRO ( 175-) C - -CA -C N 125.97 6.0

576 PRO ( 175-) C - N CA C 127.38 6.2

580 VAL ( 179-) C - C CA CB 100.68 -5.0

590 ASN ( 189-) C - CA CB CG 107.03 -5.6

592 TYR ( 191-) C - -C N CA 114.41 -4.1

593 ASP ( 192-) C - CA CB CG 107.96 -4.6

600 ASP ( 199-) C - CA CB CG 108.32 -4.3

617 HIS ( 216-) C - NE2 CD2 CG 110.51 4.0

618 PHE ( 217-) C - CA CB CG 109.30 -4.5

624 ILE ( 223-) C - -C N CA 128.92 4.0

627 PHE ( 226-) C - CA CB CG 105.59 -8.2

646 PHE ( 245-) C - N CA CB 118.31 4.6

646 PHE ( 245-) C - C CA CB 96.03 -7.4

654 PRO ( 253-) C - -CA -C N 123.48 4.4

659 PHE ( 258-) C - CA CB CG 108.13 -5.7

690 PHE ( 289-) C - CA CB CG 106.50 -7.3

691 ILE ( 290-) C - N CA CB 120.08 5.6

697 PHE ( 296-) C - CA CB CG 108.76 -5.0

699 PHE ( 298-) C - CA CB CG 108.28 -5.5

702 LEU ( 301-) C - C CA CB 101.03 -4.8

704 GLU ( 303-) C - N CA CB 117.48 4.1

705 PHE ( 304-) C - CA CB CG 108.60 -5.2

708 VAL ( 307-) C - N CA CB 117.66 4.2

708 VAL ( 307-) C - C CA CB 101.78 -4.4

709 ASN ( 308-) C - CA CB CG 107.97 -4.6

711 PHE ( 310-) C - CA CB CG 108.57 -5.2

719 ASP ( 318-) C - CA CB CG 107.75 -4.8

723 VAL ( 322-) C - -C N CA 111.81 -5.5

732 LYS ( 331-) C - N CA CB 119.68 5.4

732 LYS ( 331-) C - C CA CB 94.56 -8.2

732 LYS ( 331-) C - CA CB CG 128.36 7.1

733 ASP ( 332-) C - CA CB CG 107.06 -5.5

734 PRO ( 333-) C - -O -C N 114.10 -5.6

734 PRO ( 333-) C - -CA -C N 130.08 8.8

735 LEU ( 334-) C - N CA CB 117.63 4.2

739 ASN ( 338-) C - CA CB CG 108.25 -4.4

740 ASN ( 339-) C - CA CB CG 108.19 -4.4

752 ASN ( 351-) C - CA CB CG 108.29 -4.3

787 PHE ( 386-) C - CA CB CG 106.48 -7.3

788 ASN ( 387-) C - CA CB CG 107.41 -5.2

789 SER ( 388-) C - -C N CA 107.80 -7.7

790 VAL ( 389-) C - C CA CB 117.81 4.1

791 SER ( 390-) C - CA C O 110.26 -6.2

792 LYS ( 391-) C - N CA C 99.51 -4.2

793 ILE ( 392-) C - -O -C N 112.60 -6.5

793 ILE ( 392-) C - -CA -C N 128.99 6.4

793 ILE ( 392-) C - -C N CA 92.17 -16.4

793 ILE ( 392-) C - N CA C 92.94 -6.5

793 ILE ( 392-) C - CA C O 128.15 4.3

794 ASP ( 393-) C - -CA -C N 101.47 -7.4

794 ASP ( 393-) C - -C N CA 133.03 6.3

794 ASP ( 393-) C - N CA C 97.19 -5.0

801 PHE ( 400-) C - C CA CB 99.87 -5.4

805 PHE ( 404-) C - CA CB CG 109.30 -4.5

808 PHE ( 407-) C - CA CB CG 105.21 -8.6

809 ASN ( 408-) C - CA CB CG 108.49 -4.1

816 TYR ( 415-) C - CA CB CG 105.34 -4.3

# 40 # Note: Normal bond angle variability

Bond angles were found to deviate normally from the mean standard bond angles

(normal values for protein residues were taken from Engh and Huber [REF], for

DNA/RNA from Parkinson et al [REF]). The RMS Z-score given below is expected

to be near 1.0 for a normally restrained data set, and this is indeed

observed for very high resolution X-ray structures.

RMS Z-score for bond angles: 1.350

RMS-deviation in bond angles: 2.471

# 41 # Note: Residue hand check OK

No atoms are observed that have the wrong handedness. Be aware, though, that

WHAT CHECK might have corrected the handedness of some atoms already. The

handedness has not been corrected for any case where the problem is worse

than just an administrative discomfort.

# 42 # Warning: Chirality deviations detected

The atoms listed in the table below have an improper dihedral value

that is deviating from expected values. As the improper dihedral values

are all getting very close to ideal values in recent X-ray structures,

and as we actually do not know how big the spread around these values

should be, this check only warns for 6 sigma deviations.

Improper dihedrals are a measure of the chirality/planarity of the structure

at a specific atom. Values around -35 or +35 are expected for chiral atoms,

and values around 0 for planar atoms. Planar side chains are left out of the

calculations, these are better handled by the planarity checks.

Three numbers are given for each atom in the table. The first is the Z-score

for the improper dihedral. The second number is the measured improper

dihedral. The third number is the expected value for this atom type. A final

column contains an extra warning if the chirality for an atom is opposite

to the expected value.

49 PRO ( 52-) A - N -8.6 -33.72 -2.68

151 PRO ( 154-) A - N -6.1 -24.68 -2.68

171 GLU ( 174-) A - C 6.0 9.22 0.04

172 PRO ( 175-) A - N 8.9 29.22 -2.68

304 VAL ( 307-) A - CA 6.9 43.18 33.25

321 PRO ( 324-) A - N 6.5 20.57 -2.68

330 PRO ( 333-) A - N -6.2 -25.04 -2.68

453 PRO ( 52-) C - N -8.3 -32.36 -2.68

555 PRO ( 154-) C - N -6.1 -24.51 -2.68

576 PRO ( 175-) C - N -7.2 -28.56 -2.68

576 PRO ( 175-) C - CA -6.9 28.77 38.19

725 PRO ( 324-) C - N 6.7 21.30 -2.68

734 PRO ( 333-) C - N -6.8 -27.00 -2.68

793 ILE ( 392-) C - C 18.4 23.22 0.03

The average deviation= 1.450

# 43 # Note: Improper dihedral angle distribution OK

The RMS Z-score for all improper dihedrals in the structure is within normal

ranges.

Improper dihedral RMS Z-score : 1.290

# 44 # Error: Tau angle problems

The side chains of the residues listed in the table below contain a tau

angle (N-Calpha-C) that was found to deviate from te expected value by

more than 4.0 times the expected standard deviation. The number in the

table is the number of standard deviations this RMS value deviates from

the expected value.

794 ASP ( 393-) C - 9.02

573 THR ( 172-) C - 6.35

793 ILE ( 392-) C - 5.89

576 PRO ( 175-) C - 5.69

387 SER ( 390-) A - 5.43

170 ARG ( 173-) A - 5.30

578 ARG ( 177-) C - 4.75

574 ARG ( 173-) C - 4.72

792 LYS ( 391-) C - 4.54

226 GLN ( 229-) A - 4.03

# 45 # Note: Normal tau angle deviations

The RMS Z-score for the tau angles (N-Calpha-C) in the structure falls

within the normal range that we guess to be 0.5 - 1.5. Be aware, we

determined the tau normal distributions from 500 high-resolution X-ray

structures, rather than from CSD data, so we cannot be 100 percent certain

about these numbers.

Tau angle RMS Z-score : 1.499

# 46 # Note: Side chain planarity OK

All of the side chains of residues that have an intact planar group are

planar within expected RMS deviations.

# 47 # Note: Atoms connected to aromatic rings OK

All of the atoms that are connected to planar aromatic rings in side chains

of amino-acid residues are in the plane within expected RMS deviations.

Since there is no DNA and no protein with hydrogens, no uncalibrated

planarity check was performed.

Ramachandran Z-score : -0.184

# 48 # Note: Ramachandran Z-score OK

The score expressing how well the backbone conformations of all residues

correspond to the known allowed areas in the Ramachandran plot is within

expected ranges for well-refined structures.

Ramachandran Z-score : -0.184

# 49 # Warning: Torsion angle evaluation shows unusual residues

The residues listed in the table below contain bad or abnormal

torsion angles.

These scores give an impression of how `normal' the torsion angles in

protein residues are. All torsion angles except omega are used for

calculating a `normality' score. Average values and standard deviations were

obtained from the residues in the WHAT CHECK database. These are used to

calculate Z-scores. A residue with a Z-score of below -2.0 is poor, and a

score of less than -3.0 is worrying. For such residues more than one torsion

angle is in a highly unlikely position.

573 THR ( 172-) C - -3.1

433 PRO ( 32-) C - -2.7

49 PRO ( 52-) A - -2.7

453 PRO ( 52-) C - -2.7

308 THR ( 311-) A - -2.6

555 PRO ( 154-) C - -2.6

151 PRO ( 154-) A - -2.6

221 GLY ( 224-) A - -2.5

172 PRO ( 175-) A - -2.4

334 LYS ( 337-) A - -2.4

750 THR ( 349-) C - -2.3

623 LYS ( 222-) C - -2.3

465 VAL ( 64-) C - -2.2

61 VAL ( 64-) A - -2.2

533 ARG ( 132-) C - -2.2

564 THR ( 163-) C - -2.2

519 LEU ( 118-) C - -2.2

714 ARG ( 313-) C - -2.2

310 ARG ( 313-) A - -2.2

91 ILE ( 94-) A - -2.2

432 ARG ( 31-) C - -2.1

678 LEU ( 277-) C - -2.0

# 50 # Warning: Backbone evaluation reveals unusual conformations

The residues listed in the table below have abnormal backbone torsion

angles.

Residues with `forbidden' phi-psi combinations are listed, as well as

residues with unusual omega angles (deviating by more than 3 sigma from the

normal value). Please note that it is normal if about 5 percent of the

residues is listed here as having unusual phi-psi combinations.

22 GLY ( 25-) A - Poor phi/psi

28 ARG ( 31-) A - Omega to (next) Pro poor

30 GLY ( 33-) A - Poor phi/psi

33 GLU ( 36-) A - Poor phi/psi

48 GLY ( 51-) A - Omega to (next) Pro poor

76 GLY ( 79-) A - Omega to (next) Pro poor

93 THR ( 96-) A - Omega to (next) Pro poor

109 MET ( 112-) A - omega poor

111 MET ( 114-) A - Omega to (next) Pro poor

122 GLY ( 125-) A - Poor phi/psi

136 CYS ( 139-) A - Omega to (next) Pro poor

143 PHE ( 146-) A - Omega to (next) Pro poor

150 CYS ( 153-) A - Omega to (next) Pro poor

169 THR ( 172-) A - omega poor

170 ARG ( 173-) A - Poor phi/psi, omega poor

171 GLU ( 174-) A - Poor phi/psi, omega to (next)

172 PRO ( 175-) A - Poor phi/psi

173 ALA ( 176-) A - Poor phi/psi

179 ALA ( 182-) A - Poor phi/psi

205 GLY ( 208-) A - Poor phi/psi

221 GLY ( 224-) A - Poor phi/psi

229 LEU ( 232-) A - Omega to (next) Pro poor

249 VAL ( 252-) A - Omega to (next) Pro poor

274 LEU ( 277-) A - Omega to (next) Pro poor

315 ASP ( 318-) A - Poor phi/psi

316 GLY ( 319-) A - Poor phi/psi

320 VAL ( 323-) A - Omega to (next) Pro poor

323 LYS ( 326-) A - Omega to (next) Pro poor

328 LYS ( 331-) A - Poor phi/psi

329 ASP ( 332-) A - Poor phi/psi, omega to (next)

333 LYS ( 336-) A - Poor phi/psi

334 LYS ( 337-) A - Poor phi/psi

339 ALA ( 342-) A - Omega to (next) Pro poor

341 THR ( 344-) A - Poor phi/psi

346 THR ( 349-) A - Omega to (next) Pro poor

353 ASP ( 356-) A - Omega to (next) Pro poor

361 LYS ( 364-) A - Poor phi/psi

366 GLU ( 369-) A - Omega to (next) Pro poor

368 LYS ( 371-) A - Poor phi/psi

371 LYS ( 374-) A - Omega to (next) Pro poor

375 LYS ( 378-) A - Omega to (next) Pro poor

376 PRO ( 379-) A - Omega to (next) Pro poor

378 GLU ( 381-) A - Omega to (next) Pro poor

380 LYS ( 383-) A - Poor phi/psi

382 THR ( 385-) A - Poor phi/psi

385 SER ( 388-) A - Poor phi/psi

397 PHE ( 400-) A - Omega to (next) Pro poor

426 GLY ( 25-) C - Poor phi/psi

432 ARG ( 31-) C - Omega to (next) Pro poor

433 PRO ( 32-) C - Poor phi/psi

434 GLY ( 33-) C - Poor phi/psi, omega poor

437 GLU ( 36-) C - Poor phi/psi

452 GLY ( 51-) C - Omega to (next) Pro poor

480 GLY ( 79-) C - Omega to (next) Pro poor

497 THR ( 96-) C - Omega to (next) Pro poor

513 MET ( 112-) C - omega poor

515 MET ( 114-) C - Omega to (next) Pro poor

526 GLY ( 125-) C - Poor phi/psi

540 CYS ( 139-) C - Omega to (next) Pro poor

547 PHE ( 146-) C - Omega to (next) Pro poor

554 CYS ( 153-) C - Omega to (next) Pro poor

573 THR ( 172-) C - Poor phi/psi

574 ARG ( 173-) C - omega poor

575 GLU ( 174-) C - Poor phi/psi, omega to (next)

577 ALA ( 176-) C - Poor phi/psi

583 ALA ( 182-) C - Poor phi/psi

609 GLY ( 208-) C - Poor phi/psi

633 LEU ( 232-) C - Omega to (next) Pro poor

653 VAL ( 252-) C - Omega to (next) Pro poor

678 LEU ( 277-) C - Omega to (next) Pro poor

719 ASP ( 318-) C - Poor phi/psi

724 VAL ( 323-) C - Omega to (next) Pro poor

727 LYS ( 326-) C - Omega to (next) Pro poor

731 VAL ( 330-) C - Poor phi/psi

733 ASP ( 332-) C - Poor phi/psi, omega to (next)

743 ALA ( 342-) C - Omega to (next) Pro poor

745 THR ( 344-) C - Poor phi/psi

748 SER ( 347-) C - Poor phi/psi

750 THR ( 349-) C - Omega to (next) Pro poor

757 ASP ( 356-) C - Omega to (next) Pro poor

770 GLU ( 369-) C - Omega to (next) Pro poor

775 LYS ( 374-) C - Omega to (next) Pro poor

779 LYS ( 378-) C - Omega to (next) Pro poor

780 PRO ( 379-) C - Omega to (next) Pro poor

782 GLU ( 381-) C - Omega to (next) Pro poor

788 ASN ( 387-) C - Poor phi/psi

790 VAL ( 389-) C - omega poor

791 SER ( 390-) C - Poor phi/psi

792 LYS ( 391-) C - Poor phi/psi, omega poor

793 ILE ( 392-) C - Poor phi/psi, omega poor

801 PHE ( 400-) C - Omega to (next) Pro poor

chi-1/chi-2 correlation Z-score : -2.241

# 51 # Note: chi-1/chi-2 angle correlation Z-score OK

The score expressing how well the chi-1/chi-2 angles of all residues

correspond to the populated areas in the database is

within expected ranges for well-refined structures.

chi-1/chi-2 correlation Z-score : -2.241

# 52 # Warning: Unusual rotamers

The residues listed in the table below have a rotamer that is not seen very

often in the database of solved protein structures. This option determines

for every residue the position specific chi-1 rotamer distribution.

Thereafter it verified whether the actual residue in the molecule has the

most preferred rotamer or not. If the actual rotamer is the preferred one,

the score is 1.0. If the actual rotamer is unique, the score is 0.0. If

there are two preferred rotamers, with a population distribution of 3:2 and

your rotamer sits in the lesser populated rotamer, the score will be 0.667.

No value will be given if insufficient hits are found in the database.

It is not necessarily an error if a few residues have rotamer values below

0.3, but careful inspection of all residues with these low values could be

worth it.

677 SER ( 276-) C - 0.35

51 SER ( 54-) A - 0.37

100 ASN ( 103-) A - 0.38

203 SER ( 206-) A - 0.38

579 SER ( 178-) C - 0.38

121 ASP ( 124-) A - 0.39

455 SER ( 54-) C - 0.39

645 SER ( 244-) C - 0.39

607 SER ( 206-) C - 0.40

# 53 # Warning: Unusual backbone conformations

For the residues listed in the table below, the backbone formed by itself and

two neighbouring residues on either side is in a conformation that is not

seen very often in the database of solved protein structures. The number

given in the table is the number of similar backbone conformations in the

database with the same amino acid in the centre.

For this check, backbone conformations are compared with database structures

using C-alpha superpositions with some restraints on the backbone oxygen

positions.

A residue mentioned in the table can be part of a strange loop, or there

might be something wrong with it or its directly surrounding residues. There

are a few of these in every protein, but in any case it is worth looking at,

especially if a regular DSSP secondary structure (H or S for helix or strand)

is indicated!

109 MET ( 112-) A - 0

110 THR ( 113-) A - 0

111 MET ( 114-) A - 0

169 THR ( 172-) A - 0

170 ARG ( 173-) A - 0

171 GLU ( 174-) A - 0

172 PRO ( 175-) A - 0

220 ILE ( 223-) A - 0

306 TYR ( 309-) A - 0

307 PHE ( 310-) A - 0

314 TRP ( 317-) A - 0

315 ASP ( 318-) A - 0

328 LYS ( 331-) A - 0

348 ASN ( 351-) A - 0

360 ALA ( 363-) A - 0

367 PRO ( 370-) A - 0

379 PRO ( 382-) A - 0

380 LYS ( 383-) A - 0

382 THR ( 385-) A - 0

385 SER ( 388-) A - 0

412 TYR ( 415-) A - H 0

413 LEU ( 416-) A - 0

414 THR ( 13-) C - 0

415 VAL ( 14-) C - H 0

513 MET ( 112-) C - 0

514 THR ( 113-) C - 0

515 MET ( 114-) C - 0

547 PHE ( 146-) C - 0

548 PRO ( 147-) C - 0

572 TRP ( 171-) C - 0

573 THR ( 172-) C - 0

574 ARG ( 173-) C - 0

575 GLU ( 174-) C - 0

576 PRO ( 175-) C - 0

577 ALA ( 176-) C - 0

711 PHE ( 310-) C - 0

730 LYS ( 329-) C - 0

731 VAL ( 330-) C - 0

732 LYS ( 331-) C - 0

745 THR ( 344-) C - 0

752 ASN ( 351-) C - 0

785 LYS ( 384-) C - 0

788 ASN ( 387-) C - 0

789 SER ( 388-) C - 0

790 VAL ( 389-) C - 0

791 SER ( 390-) C - 0

792 LYS ( 391-) C - 0

793 ILE ( 392-) C - 0

794 ASP ( 393-) C - H 0

95 ASP ( 98-) A - 1

142 ASP ( 145-) A - 1

143 PHE ( 146-) A - 1

144 PRO ( 147-) A - 1

173 ALA ( 176-) A - H 1

327 VAL ( 330-) A - 1

333 LYS ( 336-) A - 1

334 LYS ( 337-) A - 1

366 GLU ( 369-) A - 1

437 GLU ( 36-) C - 1

549 MET ( 148-) C - 1

710 TYR ( 309-) C - 1

718 TRP ( 317-) C - 1

733 ASP ( 332-) C - 1

734 PRO ( 333-) C - 1

769 ILE ( 368-) C - 1

787 PHE ( 386-) C - 1

795 ARG ( 394-) C - H 1

78 MET ( 81-) A - 2

204 THR ( 207-) A - 2

312 TYR ( 315-) A - 2

340 PRO ( 343-) A - 2

368 LYS ( 371-) A - 2

369 GLU ( 372-) A - 2

499 ASP ( 98-) C - 2

546 ASP ( 145-) C - 2

# 54 # Note: Backbone conformation Z-score OK

The backbone conformation analysis gives a score that is normal for well

refined protein structures.

Backbone conformation Z-score : 0.054

# 55 # Note: Omega angle restraint OK

The omega angles for trans-peptide bonds in a structure is expected to give a

gaussian distribution with the average around +178 degrees, and a standard

deviation around 5.5. In the current structure the standard deviation agrees

with this expectation.

Omega average and std. deviation= 181.180 4.835

# 56 # Note: PRO puckering amplitude OK

Puckering amplitudes for all PRO residues are within normal ranges.

# 57 # Warning: Unusual PRO puckering phases

The proline residues listed in the table below have a puckering phase that is

not expected to occur in protein structures. Puckering parameters were

calculated by the method of Cremer and Pople [REF]. Normal PRO rings

approximately show a so-called envelope conformation with the C-gamma atom

above the plane of the ring (phi=+72 degrees), or a half-chair conformation

with C-gamma below and C-beta above the plane of the ring (phi=-90 degrees).

If phi deviates strongly from these values, this is indicative of a very

strange conformation for a PRO residue, and definitely requires a manual

check of the data. Be aware that this is a warning with a low confidence

level. See: Who checks the checkers? Four validation tools applied to eight

atomic resolution structures [REF].

29 PRO ( 32-) A - -128.0 half-chair C-delta/C-gamma (-126 degrees)

77 PRO ( 80-) A - -116.3 envelop C-gamma (-108 degrees)

94 PRO ( 97-) A - -125.3 half-chair C-delta/C-gamma (-126 degrees)

112 PRO ( 115-) A - -139.1 envelop C-delta (-144 degrees)

137 PRO ( 140-) A - -116.1 envelop C-gamma (-108 degrees)

172 PRO ( 175-) A - -59.7 half-chair C-beta/C-alpha (-54 degrees)

230 PRO ( 233-) A - 109.0 envelop C-beta (108 degrees)

275 PRO ( 278-) A - -112.6 envelop C-gamma (-108 degrees)

321 PRO ( 324-) A - -64.6 envelop C-beta (-72 degrees)

324 PRO ( 327-) A - -115.9 envelop C-gamma (-108 degrees)

330 PRO ( 333-) A - 108.1 envelop C-beta (108 degrees)

347 PRO ( 350-) A - -112.2 envelop C-gamma (-108 degrees)

354 PRO ( 357-) A - 100.2 envelop C-beta (108 degrees)

367 PRO ( 370-) A - 107.4 envelop C-beta (108 degrees)

372 PRO ( 375-) A - 102.7 envelop C-beta (108 degrees)

376 PRO ( 379-) A - -123.3 half-chair C-delta/C-gamma (-126 degrees)

377 PRO ( 380-) A - -115.8 envelop C-gamma (-108 degrees)

379 PRO ( 382-) A - -125.6 half-chair C-delta/C-gamma (-126 degrees)

453 PRO ( 52-) C - 46.4 half-chair C-delta/C-gamma (54 degrees)

481 PRO ( 80-) C - -122.8 half-chair C-delta/C-gamma (-126 degrees)

498 PRO ( 97-) C - -127.9 half-chair C-delta/C-gamma (-126 degrees)

516 PRO ( 115-) C - 106.8 envelop C-beta (108 degrees)

541 PRO ( 140-) C - 103.4 envelop C-beta (108 degrees)

576 PRO ( 175-) C - 124.6 half-chair C-beta/C-alpha (126 degrees)

634 PRO ( 233-) C - 106.0 envelop C-beta (108 degrees)

679 PRO ( 278-) C - -125.5 half-chair C-delta/C-gamma (-126 degrees)

725 PRO ( 324-) C - -61.3 half-chair C-beta/C-alpha (-54 degrees)

728 PRO ( 327-) C - -125.6 half-chair C-delta/C-gamma (-126 degrees)

751 PRO ( 350-) C - 105.1 envelop C-beta (108 degrees)

758 PRO ( 357-) C - -116.8 envelop C-gamma (-108 degrees)

780 PRO ( 379-) C - 107.4 envelop C-beta (108 degrees)

# 58 # Note: Backbone oxygen evaluation OK

All residues for which similar local backbone conformations could be found

in the WHAT CHECK database have a backbone oxygen position that has been

observed at least a few times in that database.

# 59 # Note: Peptide bond conformations

There was no need to complain about a single amino acid

# 60 # Error: Abnormally short interatomic distances

The pairs of atoms listed in the table below have an unusually short

interactomic distance; each bump is listed in only one direction.

The contact distances of all atom pairs have been checked. Two atoms are

said to `bump' if they are closer than the sum of their Van der Waals radii

minus 0.40 Angstrom. For hydrogen bonded pairs a tolerance of 0.55 Angstrom

is used. The first number in the table tells you how much shorter that

specific contact is than the acceptable limit. The second distance is the

distance between the centres of the two atoms. Although we believe that two

water atoms at 2.4 A distance are too close, we only report water pairs that

are closer than this rather short distance.

The last text-item on each line represents the status of the atom pair. If

the final column contains the text 'HB', the bump criterion was relaxed

because there could be a hydrogen bond. Similarly relaxed criteria are used

for 1--3 and 1--4 interactions (listed as 'B2' and 'B3', respectively).

If the last column is 'BF', the sum of the B-factors of the atoms is higher

than 80, which makes the appearance of the bump somewhat less severe because

the atoms probably are not there anyway. BL, on the other hand, indicates

that the bumping atoms both have a low B-factor, and that makes the bumps

more worrisome. INTRA and INTER indicate whether the clashes are between

atoms in the same asymmetric unit, or atoms in symmetry related asymmetric

units, respectively.

Bumps between atoms for which the sum of their occupancies is lower than one

are not reported. If the MODEL number does not exist (as is the case in most

X-ray files), a minus sign is printed instead.

170 ARG ( 173-) A - N <--> 171 GLU ( 174-) A - N 0.45 2.15 INTRA BL

793 ILE ( 392-) C - CB <--> 794 ASP ( 393-) C - N 0.42 2.28 INTRA BL

148 HIS ( 151-) A - NE2 <--> 150 CYS ( 153-) A - SG 0.40 2.90 INTRA BL

39 LYS ( 42-) A - C <--> 169 THR ( 172-) A - CB 0.39 2.81 INTRA BL

732 LYS ( 331-) C - CG <--> 791 SER ( 390-) C - CB 0.39 2.81 INTRA BL

57 TYR ( 60-) A - CD1 <--> 136 CYS ( 139-) A - SG 0.37 3.03 INTRA BL

540 CYS ( 139-) C - SG <--> 542 MET ( 141-) C - CE 0.37 3.03 INTRA BL

708 VAL ( 307-) C - CB <--> 732 LYS ( 331-) C - CB 0.35 2.85 INTRA BL

168 TRP ( 171-) A - NE1 <--> 195 VAL ( 198-) A - CG1 0.35 2.75 INTRA BL

168 TRP ( 171-) A - NE1 <--> 210 MET ( 213-) A - CE 0.35 2.75 INTRA BL

169 THR ( 172-) A - C <--> 171 GLU ( 174-) A - N 0.35 2.55 INTRA BL

281 THR ( 284-) A - CG2 <--> 283 MET ( 286-) A - SD 0.34 3.06 INTRA BL

792 LYS ( 391-) C - C <--> 793 ILE ( 392-) C - CA 0.34 1.96 INTRA BL

148 HIS ( 151-) A - CD2 <--> 150 CYS ( 153-) A - SG 0.34 3.06 INTRA BL

143 PHE ( 146-) A - CD1 <--> 283 MET ( 286-) A - SD 0.34 3.06 INTRA BL

168 TRP ( 171-) A - CZ2 <--> 210 MET ( 213-) A - SD 0.34 3.06 INTRA BL

558 PHE ( 157-) C - CB <--> 614 MET ( 213-) C - SD 0.33 3.07 INTRA BL

794 ASP ( 393-) C - N <--> 795 ARG ( 394-) C - N 0.33 2.27 INTRA BL

638 THR ( 237-) C - CG2 <--> 667 MET ( 266-) C - SD 0.33 3.07 INTRA BL

635 CYS ( 234-) C - SG <--> 690 PHE ( 289-) C - CE2 0.33 3.07 INTRA BL

231 CYS ( 234-) A - SG <--> 286 PHE ( 289-) A - CE2 0.33 3.07 INTRA BL

687 MET ( 286-) C - SD <--> 691 ILE ( 290-) C - CD1 0.33 3.07 INTRA BL

145 MET ( 148-) A - SD <--> 412 TYR ( 415-) A - CB 0.33 3.07 INTRA BL

300 GLU ( 303-) A - CB <--> 331 LEU ( 334-) A - N 0.33 2.77 INTRA BL

63 PHE ( 66-) A - CB <--> 128 MET ( 131-) A - SD 0.33 3.07 INTRA BL

326 LYS ( 329-) A - C <--> 387 SER ( 390-) A - N 0.32 2.78 INTRA BL

573 THR ( 172-) C - C <--> 580 VAL ( 179-) C - CB 0.32 2.88 INTRA BL

704 GLU ( 303-) C - CB <--> 735 LEU ( 334-) C - N 0.31 2.79 INTRA BL

85 ASN ( 88-) A - ND2 <--> 111 MET ( 114-) A - SD 0.31 2.99 INTRA BL

57 TYR ( 60-) A - CE1 <--> 136 CYS ( 139-) A - SG 0.31 3.09 INTRA BL

138 MET ( 141-) A - SD <--> 148 HIS ( 151-) A - ND1 0.31 2.99 INTRA BL

326 LYS ( 329-) A - CA <--> 386 VAL ( 389-) A - N 0.31 2.79 INTRA BL

573 THR ( 172-) C - CB <--> 577 ALA ( 176-) C - N 0.31 2.79 INTRA BL

573 THR ( 172-) C - CA <--> 577 ALA ( 176-) C - N 0.30 2.80 INTRA BL

730 LYS ( 329-) C - CB <--> 790 VAL ( 389-) C - N 0.30 2.80 INTRA BL

145 MET ( 148-) A - SD <--> 412 TYR ( 415-) A - C 0.30 3.10 INTRA BL

574 ARG ( 173-) C - N <--> 580 VAL ( 179-) C - CA 0.30 2.80 INTRA BL

735 LEU ( 334-) C - N <--> 801 PHE ( 400-) C - CZ 0.30 2.80 INTRA BL

646 PHE ( 245-) C - CZ <--> 801 PHE ( 400-) C - CD1 0.30 2.90 INTRA BL

328 LYS ( 331-) A - CB <--> 386 VAL ( 389-) A - CG1 0.30 2.90 INTRA BL

735 LEU ( 334-) C - C <--> 801 PHE ( 400-) C - CE1 0.29 2.91 INTRA BL

791 SER ( 390-) C - CB <--> 793 ILE ( 392-) C - C 0.29 2.91 INTRA BL

300 GLU ( 303-) A - CB <--> 330 PRO ( 333-) A - C 0.29 2.91 INTRA BL

41 ASP ( 44-) A - N <--> 169 THR ( 172-) A - CG2 0.29 2.81 INTRA BL

168 TRP ( 171-) A - CB <--> 176 VAL ( 179-) A - CG1 0.29 2.91 INTRA BL

166 TYR ( 169-) A - CE1 <--> 210 MET ( 213-) A - CB 0.29 2.91 INTRA BL

731 VAL ( 330-) C - N <--> 790 VAL ( 389-) C - CB 0.29 2.81 INTRA BL

331 LEU ( 334-) A - CA <--> 397 PHE ( 400-) A - CE1 0.29 2.91 INTRA BL

332 ILE ( 335-) A - N <--> 397 PHE ( 400-) A - CE1 0.29 2.81 INTRA BL

306 TYR ( 309-) A - CB <--> 319 VAL ( 322-) A - N 0.29 2.81 INTRA BL

710 TYR ( 309-) C - CB <--> 723 VAL ( 322-) C - N 0.29 2.81 INTRA BL

459 MET ( 58-) C - SD <--> 542 MET ( 141-) C - N 0.29 3.01 INTRA BL

704 GLU ( 303-) C - CB <--> 735 LEU ( 334-) C - CB 0.28 2.92 INTRA BL

168 TRP ( 171-) A - C <--> 171 GLU ( 174-) A - CA 0.28 2.92 INTRA BL

572 TRP ( 171-) C - CE2 <--> 614 MET ( 213-) C - CG 0.28 2.92 INTRA BL

242 PHE ( 245-) A - CA <--> 329 ASP ( 332-) A - CB 0.28 2.92 INTRA BL

553 ALA ( 152-) C - CB <--> 617 HIS ( 216-) C - NE2 0.28 2.82 INTRA BL

252 ARG ( 255-) A - NH1 <--> 329 ASP ( 332-) A - CA 0.28 2.82 INTRA BL

704 GLU ( 303-) C - CG <--> 732 LYS ( 331-) C - CG 0.28 2.92 INTRA BL

688 ASP ( 287-) C - CA <--> 691 ILE ( 290-) C - CD1 0.28 2.92 INTRA BL

623 LYS ( 222-) C - CE <--> 625 GLY ( 224-) C - N 0.28 2.82 INTRA BL

704 GLU ( 303-) C - CG <--> 732 LYS ( 331-) C - CB 0.28 2.92 INTRA BL

119 THR ( 122-) A - CG2 <--> 123 THR ( 126-) A - N 0.28 2.82 INTRA BL

708 VAL ( 307-) C - N <--> 732 LYS ( 331-) C - CD 0.28 2.82 INTRA BL

154 PHE ( 157-) A - CE1 <--> 166 TYR ( 169-) A - CE2 0.28 2.92 INTRA BL

708 VAL ( 307-) C - CG2 <--> 732 LYS ( 331-) C - N 0.28 2.82 INTRA BL

242 PHE ( 245-) A - CZ <--> 397 PHE ( 400-) A - CD1 0.28 2.92 INTRA BL

304 VAL ( 307-) A - CG2 <--> 328 LYS ( 331-) A - CG 0.28 2.92 INTRA BL

149 ALA ( 152-) A - CB <--> 213 HIS ( 216-) A - NE2 0.28 2.82 INTRA BL

551 ALA ( 150-) C - CB <--> 619 HIS ( 218-) C - NE2 0.28 2.82 INTRA BL

185 LEU ( 188-) A - CD1 <--> 188 TYR ( 191-) A - N 0.28 2.82 INTRA BL

242 PHE ( 245-) A - CE1 <--> 330 PRO ( 333-) A - C 0.28 2.92 INTRA BL

39 LYS ( 42-) A - CB <--> 169 THR ( 172-) A - CA 0.28 2.92 INTRA BL

707 THR ( 306-) C - CG2 <--> 732 LYS ( 331-) C - CD 0.28 2.92 INTRA BL

304 VAL ( 307-) A - CG2 <--> 329 ASP ( 332-) A - N 0.27 2.83 INTRA BL

439 VAL ( 38-) C - CG1 <--> 568 VAL ( 167-) C - C 0.27 2.93 INTRA BL

543 HIS ( 142-) C - NE2 <--> 545 GLU ( 144-) C - CD 0.27 2.83 INTRA BL

74 PHE ( 77-) A - CE2 <--> 76 GLY ( 79-) A - N 0.27 2.83 INTRA BL

731 VAL ( 330-) C - CG2 <--> 793 ILE ( 392-) C - CG2 0.27 2.93 INTRA BL

41 ASP ( 44-) A - CB <--> 169 THR ( 172-) A - CG2 0.27 2.93 INTRA BL

12 PHE ( 15-) A - CZ <--> 81 LEU ( 84-) A - CD1 0.27 2.93 INTRA BL

574 ARG ( 173-) C - CB <--> 580 VAL ( 179-) C - CA 0.27 2.93 INTRA BL

139 HIS ( 142-) A - NE2 <--> 141 GLU ( 144-) A - CD 0.27 2.83 INTRA BL

105 VAL ( 108-) A - N <--> 130 LEU ( 133-) A - CD1 0.27 2.83 INTRA BL

35 VAL ( 38-) A - CG2 <--> 164 VAL ( 167-) A - C 0.27 2.93 INTRA BL

74 PHE ( 77-) A - CD2 <--> 76 GLY ( 79-) A - N 0.27 2.83 INTRA BL

40 THR ( 43-) A - C <--> 169 THR ( 172-) A - N 0.27 2.83 INTRA BL

543 HIS ( 142-) C - NE2 <--> 545 GLU ( 144-) C - CG 0.27 2.83 INTRA BL

15 ILE ( 18-) A - CD1 <--> 74 PHE ( 77-) A - CG 0.27 2.93 INTRA BL

300 GLU ( 303-) A - CB <--> 331 LEU ( 334-) A - CB 0.27 2.93 INTRA BL

328 LYS ( 331-) A - CA <--> 390 ASP ( 393-) A - CB 0.27 2.93 INTRA BL

570 TYR ( 169-) C - CE1 <--> 614 MET ( 213-) C - CB 0.27 2.93 INTRA BL

96 THR ( 99-) A - CG2 <--> 154 PHE ( 157-) A - CE2 0.27 2.93 INTRA BL

707 THR ( 306-) C - CA <--> 710 TYR ( 309-) C - CD2 0.27 2.93 INTRA BL

570 TYR ( 169-) C - CB <--> 612 VAL ( 211-) C - CG1 0.27 2.93 INTRA BL

13 THR ( 16-) A - CA <--> 16 LEU ( 19-) A - CD1 0.27 2.93 INTRA BL

45 THR ( 48-) A - CG2 <--> 60 ASP ( 63-) A - CB 0.27 2.93 INTRA BL

12 PHE ( 15-) A - CA <--> 15 ILE ( 18-) A - CG2 0.27 2.93 INTRA BL

237 LEU ( 240-) A - C <--> 255 PHE ( 258-) A - CE1 0.27 2.93 INTRA BL

500 THR ( 99-) C - CG2 <--> 558 PHE ( 157-) C - CE2 0.27 2.93 INTRA BL

519 LEU ( 118-) C - CG <--> 531 THR ( 130-) C - CG2 0.27 2.93 INTRA BL

645 SER ( 244-) C - CB <--> 733 ASP ( 332-) C - CG 0.27 2.93 INTRA BL

660 GLY ( 259-) C - CA <--> 702 LEU ( 301-) C - CD1 0.27 2.93 INTRA BL

708 VAL ( 307-) C - CG2 <--> 790 VAL ( 389-) C - CG1 0.27 2.93 INTRA BL

731 VAL ( 330-) C - CA <--> 790 VAL ( 389-) C - CG1 0.27 2.93 INTRA BL

558 PHE ( 157-) C - CE1 <--> 570 TYR ( 169-) C - CE2 0.27 2.93 INTRA BL

328 LYS ( 331-) A - CE <--> 389 ILE ( 392-) A - CD1 0.26 2.94 INTRA BL

326 LYS ( 329-) A - CA <--> 386 VAL ( 389-) A - CB 0.26 2.94 INTRA BL

81 LEU ( 84-) A - CB <--> 118 ILE ( 121-) A - CG2 0.26 2.94 INTRA BL

42 ILE ( 45-) A - CG2 <--> 61 VAL ( 64-) A - CG2 0.26 2.94 INTRA BL

558 PHE ( 157-) C - CB <--> 614 MET ( 213-) C - CB 0.26 2.94 INTRA BL

40 THR ( 43-) A - CG2 <--> 166 TYR ( 169-) A - CE1 0.26 2.94 INTRA BL

63 PHE ( 66-) A - CZ <--> 154 PHE ( 157-) A - CE2 0.26 2.94 INTRA BL

331 LEU ( 334-) A - C <--> 397 PHE ( 400-) A - CE1 0.26 2.94 INTRA BL

467 PHE ( 66-) C - CZ <--> 558 PHE ( 157-) C - CE2 0.26 2.94 INTRA BL

168 TRP ( 171-) A - CD2 <--> 176 VAL ( 179-) A - CG2 0.26 2.94 INTRA BL

577 ALA ( 176-) C - CB <--> 578 ARG ( 177-) C - N 0.26 2.44 INTRA BL

166 TYR ( 169-) A - CB <--> 208 VAL ( 211-) A - CG1 0.26 2.94 INTRA BL

592 TYR ( 191-) C - CE1 <--> 620 LEU ( 219-) C - CD2 0.26 2.94 INTRA BL

199 ILE ( 202-) A - CG1 <--> 206 GLU ( 209-) A - CG 0.26 2.94 INTRA BL

277 VAL ( 280-) A - CG1 <--> 279 TYR ( 282-) A - CE2 0.26 2.94 INTRA BL

323 LYS ( 326-) A - CG <--> 325 LYS ( 328-) A - CB 0.26 2.94 INTRA BL

681 VAL ( 280-) C - CG1 <--> 683 TYR ( 282-) C - CE2 0.26 2.94 INTRA BL

572 TRP ( 171-) C - CE3 <--> 580 VAL ( 179-) C - CG2 0.26 2.94 INTRA BL

23 TYR ( 26-) A - CE2 <--> 71 ARG ( 74-) A - CD 0.26 2.94 INTRA BL

646 PHE ( 245-) C - CE1 <--> 797 SER ( 396-) C - CB 0.26 2.94 INTRA BL

445 ASP ( 44-) C - CG <--> 574 ARG ( 173-) C - CB 0.26 2.94 INTRA BL

306 TYR ( 309-) A - CB <--> 319 VAL ( 322-) A - CG2 0.26 2.94 INTRA BL

656 ARG ( 255-) C - NH2 <--> 733 ASP ( 332-) C - CA 0.26 2.84 INTRA BL

723 VAL ( 322-) C - CG1 <--> 790 VAL ( 389-) C - CA 0.26 2.94 INTRA BL

328 LYS ( 331-) A - CE <--> 389 ILE ( 392-) A - CG2 0.26 2.94 INTRA BL

570 TYR ( 169-) C - CZ <--> 614 MET ( 213-) C - CB 0.26 2.94 INTRA BL

304 VAL ( 307-) A - CG2 <--> 328 LYS ( 331-) A - CB 0.26 2.94 INTRA BL

572 TRP ( 171-) C - CD2 <--> 599 VAL ( 198-) C - CG1 0.26 2.94 INTRA BL

154 PHE ( 157-) A - CZ <--> 166 TYR ( 169-) A - CE2 0.26 2.94 INTRA BL

517 ASN ( 116-) C - CB <--> 533 ARG ( 132-) C - CG 0.26 2.94 INTRA BL

707 THR ( 306-) C - CG2 <--> 732 LYS ( 331-) C - CE 0.26 2.94 INTRA BL

711 PHE ( 310-) C - CA <--> 722 SER ( 321-) C - CB 0.26 2.94 INTRA BL

115 LEU ( 118-) A - CB <--> 127 THR ( 130-) A - CG2 0.26 2.94 INTRA BL

563 TYR ( 162-) C - CB <--> 567 GLU ( 166-) C - CG 0.26 2.94 INTRA BL

573 THR ( 172-) C - CG2 <--> 577 ALA ( 176-) C - CA 0.26 2.94 INTRA BL

300 GLU ( 303-) A - CG <--> 328 LYS ( 331-) A - CD 0.26 2.94 INTRA BL

256 GLY ( 259-) A - CA <--> 298 LEU ( 301-) A - CD2 0.26 2.94 INTRA BL

446 ILE ( 45-) C - CG2 <--> 465 VAL ( 64-) C - CG2 0.26 2.94 INTRA BL

35 VAL ( 38-) A - CG2 <--> 164 VAL ( 167-) A - CA 0.26 2.94 INTRA BL

451 PHE ( 50-) C - CZ <--> 461 TYR ( 60-) C - CB 0.26 2.94 INTRA BL

166 TYR ( 169-) A - CE1 <--> 210 MET ( 213-) A - CG 0.26 2.94 INTRA BL

196 ASP ( 199-) A - CB <--> 211 THR ( 214-) A - CG2 0.26 2.94 INTRA BL

573 THR ( 172-) C - CB <--> 575 GLU ( 174-) C - C 0.26 2.94 INTRA BL

27 LEU ( 30-) A - CG <--> 31 LEU ( 34-) A - CD1 0.26 2.94 INTRA BL

710 TYR ( 309-) C - CB <--> 723 VAL ( 322-) C - CG2 0.26 2.94 INTRA BL

12 PHE ( 15-) A - CE2 <--> 16 LEU ( 19-) A - CD1 0.26 2.94 INTRA BL

693 VAL ( 292-) C - CG2 <--> 808 PHE ( 407-) C - CE2 0.26 2.94 INTRA BL

20 LEU ( 23-) A - CD2 <--> 90 LYS ( 93-) A - CD 0.26 2.94 INTRA BL

532 MET ( 131-) C - CG <--> 534 LEU ( 133-) C - CD1 0.26 2.94 INTRA BL

237 LEU ( 240-) A - CB <--> 255 PHE ( 258-) A - CZ 0.26 2.94 INTRA BL

793 ILE ( 392-) C - CG1 <--> 795 ARG ( 394-) C - CG 0.26 2.94 INTRA BL

646 PHE ( 245-) C - CA <--> 794 ASP ( 393-) C - CG 0.26 2.94 INTRA BL

573 THR ( 172-) C - CB <--> 576 PRO ( 175-) C - C 0.26 2.94 INTRA BL

289 VAL ( 292-) A - CG2 <--> 404 PHE ( 407-) A - CE2 0.26 2.94 INTRA BL

185 LEU ( 188-) A - CG <--> 188 TYR ( 191-) A - CE2 0.26 2.94 INTRA BL

485 LEU ( 84-) C - CB <--> 522 ILE ( 121-) C - CG2 0.26 2.94 INTRA BL

589 LEU ( 188-) C - CG <--> 592 TYR ( 191-) C - CZ 0.26 2.94 INTRA BL

558 PHE ( 157-) C - CZ <--> 570 TYR ( 169-) C - CE2 0.26 2.94 INTRA BL

304 VAL ( 307-) A - CG2 <--> 328 LYS ( 331-) A - C 0.26 2.94 INTRA BL

220 ILE ( 223-) A - CG2 <--> 224 VAL ( 227-) A - CG2 0.26 2.94 INTRA BL

328 LYS ( 331-) A - CE <--> 386 VAL ( 389-) A - CG1 0.26 2.94 INTRA BL

556 LEU ( 155-) C - CB <--> 616 THR ( 215-) C - CG2 0.26 2.94 INTRA BL

544 LEU ( 143-) C - CD1 <--> 678 LEU ( 277-) C - CD1 0.26 2.94 INTRA BL

416 PHE ( 15-) C - CE1 <--> 485 LEU ( 84-) C - CD1 0.26 2.94 INTRA BL

130 LEU ( 133-) A - CD2 <--> 132 VAL ( 135-) A - CG1 0.26 2.94 INTRA BL

300 GLU ( 303-) A - CD <--> 328 LYS ( 331-) A - CG 0.26 2.94 INTRA BL

286 PHE ( 289-) A - CE2 <--> 290 CYS ( 293-) A - SG 0.26 3.14 INTRA BL

224 VAL ( 227-) A - CG1 <--> 229 LEU ( 232-) A - CD1 0.26 2.94 INTRA BL

736 ILE ( 335-) C - CD1 <--> 800 ALA ( 399-) C - CB 0.26 2.94 INTRA BL

312 TYR ( 315-) A - CE2 <--> 314 TRP ( 317-) A - CB 0.26 2.94 INTRA BL

648 LEU ( 247-) C - CD1 <--> 652 SER ( 251-) C - CB 0.26 2.94 INTRA BL

693 VAL ( 292-) C - CG1 <--> 808 PHE ( 407-) C - CZ 0.26 2.94 INTRA BL

188 TYR ( 191-) A - CE1 <--> 216 LEU ( 219-) A - CD1 0.26 2.94 INTRA BL

443 LYS ( 42-) C - CG <--> 575 GLU ( 174-) C - CA 0.26 2.94 INTRA BL

500 THR ( 99-) C - CG2 <--> 558 PHE ( 157-) C - CZ 0.26 2.94 INTRA BL

710 TYR ( 309-) C - C <--> 723 VAL ( 322-) C - N 0.26 2.84 INTRA BL

732 LYS ( 331-) C - CA <--> 791 SER ( 390-) C - CB 0.26 2.94 INTRA BL

589 LEU ( 188-) C - CG <--> 592 TYR ( 191-) C - CE2 0.26 2.94 INTRA BL

58 THR ( 61-) A - CG2 <--> 133 ARG ( 136-) A - CG 0.25 2.95 INTRA BL

252 ARG ( 255-) A - CG <--> 301 PHE ( 304-) A - CD1 0.25 2.95 INTRA BL

44 VAL ( 47-) A - CG2 <--> 59 ILE ( 62-) A - CD1 0.25 2.95 INTRA BL

172 PRO ( 175-) A - C <--> 176 VAL ( 179-) A - CG1 0.25 2.95 INTRA BL

667 MET ( 266-) C - SD <--> 694 CYS ( 293-) C - SG 0.25 3.20 INTRA BL

565 ARG ( 164-) C - CD <--> 603 ILE ( 202-) C - CG2 0.25 2.95 INTRA BL

244 LEU ( 247-) A - CD1 <--> 248 SER ( 251-) A - CB 0.25 2.95 INTRA BL

43 PHE ( 46-) A - CD1 <--> 178 VAL ( 181-) A - CG2 0.25 2.95 INTRA BL

154 PHE ( 157-) A - CD1 <--> 166 TYR ( 169-) A - CE2 0.25 2.95 INTRA BL

660 GLY ( 259-) C - CA <--> 702 LEU ( 301-) C - CD2 0.25 2.95 INTRA BL

327 VAL ( 330-) A - CB <--> 387 SER ( 390-) A - CB 0.25 2.95 INTRA BL

20 LEU ( 23-) A - CG <--> 90 LYS ( 93-) A - CE 0.25 2.95 INTRA BL

12 PHE ( 15-) A - CE1 <--> 81 LEU ( 84-) A - CD1 0.25 2.95 INTRA BL

451 PHE ( 50-) C - CE2 <--> 454 VAL ( 53-) C - CG2 0.25 2.95 INTRA BL

460 GLU ( 59-) C - CB <--> 506 LYS ( 105-) C - CE 0.25 2.95 INTRA BL

711 PHE ( 310-) C - CB <--> 724 VAL ( 323-) C - CB 0.25 2.95 INTRA BL

735 LEU ( 334-) C - CA <--> 801 PHE ( 400-) C - CE1 0.25 2.95 INTRA BL

159 TYR ( 162-) A - CD1 <--> 163 GLU ( 166-) A - CD 0.25 2.95 INTRA BL

319 VAL ( 322-) A - CG1 <--> 386 VAL ( 389-) A - CG1 0.25 2.95 INTRA BL

15 ILE ( 18-) A - CD1 <--> 74 PHE ( 77-) A - CD2 0.25 2.95 INTRA BL

704 GLU ( 303-) C - CB <--> 734 PRO ( 333-) C - C 0.25 2.95 INTRA BL

572 TRP ( 171-) C - CZ2 <--> 614 MET ( 213-) C - CE 0.25 2.95 INTRA BL

289 VAL ( 292-) A - CG1 <--> 404 PHE ( 407-) A - CE1 0.25 2.95 INTRA BL

451 PHE ( 50-) C - CZ <--> 461 TYR ( 60-) C - CD2 0.25 2.95 INTRA BL

518 LYS ( 117-) C - CD <--> 530 TYR ( 129-) C - CE1 0.25 2.95 INTRA BL

114 LYS ( 117-) A - CD <--> 126 TYR ( 129-) A - CE1 0.25 2.95 INTRA BL

234 THR ( 237-) A - CG2 <--> 263 MET ( 266-) A - CE 0.25 2.95 INTRA BL

557 LYS ( 156-) C - CE <--> 613 VAL ( 212-) C - CG2 0.25 2.95 INTRA BL

519 LEU ( 118-) C - CB <--> 531 THR ( 130-) C - CG2 0.25 2.95 INTRA BL

63 PHE ( 66-) A - CB <--> 128 MET ( 131-) A - CE 0.25 2.95 INTRA BL

96 THR ( 99-) A - CG2 <--> 154 PHE ( 157-) A - CZ 0.25 2.95 INTRA BL

697 PHE ( 296-) C - CZ <--> 804 LEU ( 403-) C - CD2 0.25 2.95 INTRA BL

40 THR ( 43-) A - CG2 <--> 166 TYR ( 169-) A - CZ 0.25 2.95 INTRA BL

506 LYS ( 105-) C - CE <--> 539 GLU ( 138-) C - CG 0.25 2.95 INTRA BL

246 ARG ( 249-) A - CD <--> 327 VAL ( 330-) A - CG1 0.25 2.95 INTRA BL

812 TYR ( 411-) C - CE1 <--> 816 TYR ( 415-) C - CE2 0.25 2.95 INTRA BL

564 THR ( 163-) C - CG2 <--> 567 GLU ( 166-) C - CD 0.25 2.95 INTRA BL

638 THR ( 237-) C - CG2 <--> 667 MET ( 266-) C - CE 0.25 2.95 INTRA BL

289 VAL ( 292-) A - CG1 <--> 404 PHE ( 407-) A - CZ 0.25 2.95 INTRA BL

532 MET ( 131-) C - CE <--> 534 LEU ( 133-) C - CD2 0.25 2.95 INTRA BL

572 TRP ( 171-) C - CG <--> 599 VAL ( 198-) C - CG1 0.25 2.95 INTRA BL

646 PHE ( 245-) C - CD1 <--> 797 SER ( 396-) C - CB 0.25 2.95 INTRA BL

58 THR ( 61-) A - CG2 <--> 133 ARG ( 136-) A - CA 0.25 2.95 INTRA BL

580 VAL ( 179-) C - CG1 <--> 599 VAL ( 198-) C - CG2 0.25 2.95 INTRA BL

454 VAL ( 53-) C - CG2 <--> 592 TYR ( 191-) C - CE2 0.25 2.95 INTRA BL

647 TRP ( 246-) C - CZ2 <--> 798 ARG ( 397-) C - CD 0.25 2.95 INTRA BL

416 PHE ( 15-) C - CE1 <--> 485 LEU ( 84-) C - CD2 0.25 2.95 INTRA BL

210 MET ( 213-) A - SD <--> 211 THR ( 214-) A - N 0.25 2.95 INTRA BL

572 TRP ( 171-) C - CZ2 <--> 614 MET ( 213-) C - CG 0.25 2.95 INTRA BL

47 PHE ( 50-) A - CZ <--> 57 TYR ( 60-) A - CB 0.25 2.95 INTRA BL

403 ILE ( 406-) A - CD1 <--> 407 VAL ( 410-) A - CG2 0.25 2.95 INTRA BL

39 LYS ( 42-) A - CB <--> 169 THR ( 172-) A - CB 0.25 2.95 INTRA BL

293 PHE ( 296-) A - CE1 <--> 400 LEU ( 403-) A - CD2 0.25 2.95 INTRA BL

47 PHE ( 50-) A - CE1 <--> 57 TYR ( 60-) A - CD2 0.25 2.95 INTRA BL

419 ILE ( 18-) C - CG2 <--> 478 PHE ( 77-) C - CZ 0.25 2.95 INTRA BL

656 ARG ( 255-) C - CZ <--> 733 ASP ( 332-) C - CG 0.25 2.95 INTRA BL

168 TRP ( 171-) A - CZ2 <--> 210 MET ( 213-) A - CE 0.25 2.95 INTRA BL

81 LEU ( 84-) A - CD1 <--> 83 LEU ( 86-) A - CD1 0.25 2.95 INTRA BL

554 CYS ( 153-) C - CB <--> 618 PHE ( 217-) C - CE1 0.25 2.95 INTRA BL

255 PHE ( 258-) A - CE1 <--> 259 THR ( 262-) A - CG2 0.25 2.95 INTRA BL

473 ASP ( 72-) C - CG <--> 476 LEU ( 75-) C - CD1 0.25 2.95 INTRA BL

185 LEU ( 188-) A - CG <--> 188 TYR ( 191-) A - CD2 0.25 2.95 INTRA BL

608 THR ( 207-) C - CG2 <--> 611 TYR ( 210-) C - CE1 0.25 2.95 INTRA BL

326 LYS ( 329-) A - CG <--> 386 VAL ( 389-) A - CG2 0.25 2.95 INTRA BL

547 PHE ( 146-) C - CD1 <--> 685 THR ( 284-) C - CG2 0.25 2.95 INTRA BL

408 TYR ( 411-) A - CE1 <--> 412 TYR ( 415-) A - CE2 0.25 2.95 INTRA BL

194 THR ( 197-) A - CG2 <--> 213 HIS ( 216-) A - CB 0.25 2.95 INTRA BL

168 TRP ( 171-) A - CE3 <--> 176 VAL ( 179-) A - CB 0.25 2.95 INTRA BL

145 MET ( 148-) A - CE <--> 413 LEU ( 416-) A - CD2 0.25 2.95 INTRA BL

293 PHE ( 296-) A - CD1 <--> 332 ILE ( 335-) A - CD1 0.25 2.95 INTRA BL

37 GLU ( 40-) A - CA <--> 165 VAL ( 168-) A - CG2 0.25 2.95 INTRA BL

734 PRO ( 333-) C - C <--> 801 PHE ( 400-) C - CE1 0.25 2.95 INTRA BL

791 SER ( 390-) C - C <--> 793 ILE ( 392-) C - C 0.25 2.95 INTRA BL

693 VAL ( 292-) C - CG1 <--> 808 PHE ( 407-) C - CE1 0.25 2.95 INTRA BL

506 LYS ( 105-) C - CE <--> 539 GLU ( 138-) C - CA 0.25 2.95 INTRA BL

222 TYR ( 225-) A - CE2 <--> 226 GLN ( 229-) A - CD 0.25 2.95 INTRA BL

790 VAL ( 389-) C - N <--> 791 SER ( 390-) C - N 0.25 2.35 INTRA BL

88 ALA ( 91-) A - CB <--> 114 LYS ( 117-) A - CE 0.25 2.95 INTRA BL

231 CYS ( 234-) A - SG <--> 232 ILE ( 235-) A - N 0.25 2.95 INTRA BL

235 VAL ( 238-) A - CG1 <--> 263 MET ( 266-) A - CE 0.25 2.95 INTRA BL

119 THR ( 122-) A - CG2 <--> 123 THR ( 126-) A - CB 0.25 2.95 INTRA BL

492 ALA ( 91-) C - CB <--> 518 LYS ( 117-) C - CE 0.25 2.95 INTRA BL

439 VAL ( 38-) C - CG1 <--> 569 VAL ( 168-) C - CG2 0.25 2.95 INTRA BL

716 TYR ( 315-) C - CE2 <--> 718 TRP ( 317-) C - CD2 0.25 2.95 INTRA BL

161 ARG ( 164-) A - CD <--> 199 ILE ( 202-) A - CD1 0.25 2.95 INTRA BL

98 PHE ( 101-) A - CE2 <--> 130 LEU ( 133-) A - CD2 0.25 2.95 INTRA BL

81 LEU ( 84-) A - CD2 <--> 83 LEU ( 86-) A - CD1 0.25 2.95 INTRA BL

697 PHE ( 296-) C - CE1 <--> 804 LEU ( 403-) C - CD2 0.25 2.95 INTRA BL

656 ARG ( 255-) C - NE <--> 705 PHE ( 304-) C - CD1 0.25 2.85 INTRA BL

323 LYS ( 326-) A - CD <--> 325 LYS ( 328-) A - CD 0.25 2.95 INTRA BL

328 LYS ( 331-) A - N <--> 387 SER ( 390-) A - CA 0.25 2.85 INTRA BL

252 ARG ( 255-) A - NH1 <--> 301 PHE ( 304-) A - CD1 0.25 2.85 INTRA BL

704 GLU ( 303-) C - CD <--> 732 LYS ( 331-) C - CG 0.25 2.95 INTRA BL

488 ASN ( 87-) C - ND2 <--> 490 LEU ( 89-) C - CD1 0.24 2.86 INTRA BL

138 MET ( 141-) A - SD <--> 148 HIS ( 151-) A - CE1 0.24 3.16 INTRA BL

295 PHE ( 298-) A - CE1 <--> 299 ILE ( 302-) A - CD1 0.24 2.96 INTRA BL

650 ARG ( 249-) C - CD <--> 731 VAL ( 330-) C - CG1 0.24 2.96 INTRA BL

5 LEU ( 8-) A - CD2 <--> 8 ASN ( 11-) A - CB 0.24 2.96 INTRA BL

793 ILE ( 392-) C - CG1 <--> 795 ARG ( 394-) C - NE 0.24 2.86 INTRA BL

323 LYS ( 326-) A - CD <--> 325 LYS ( 328-) A - CB 0.24 2.96 INTRA BL

656 ARG ( 255-) C - NH2 <--> 708 VAL ( 307-) C - CG1 0.24 2.86 INTRA BL

468 ARG ( 67-) C - NE <--> 529 LEU ( 128-) C - CD1 0.24 2.86 INTRA BL

656 ARG ( 255-) C - NH1 <--> 733 ASP ( 332-) C - CB 0.24 2.86 INTRA BL

793 ILE ( 392-) C - CG2 <--> 794 ASP ( 393-) C - N 0.24 2.76 INTRA BL

143 PHE ( 146-) A - CZ <--> 218 ARG ( 221-) A - NH2 0.24 2.86 INTRA BL

246 ARG ( 249-) A - NH1 <--> 327 VAL ( 330-) A - CG2 0.24 2.86 INTRA BL

47 PHE ( 50-) A - CE1 <--> 57 TYR ( 60-) A - CB 0.24 2.96 INTRA BL

735 LEU ( 334-) C - N <--> 801 PHE ( 400-) C - CE1 0.24 2.86 INTRA BL

252 ARG ( 255-) A - CZ <--> 301 PHE ( 304-) A - CD1 0.24 2.96 INTRA BL

143 PHE ( 146-) A - CD2 <--> 218 ARG ( 221-) A - NH1 0.24 2.86 INTRA BL

486 ARG ( 85-) C - NH2 <--> 521 ARG ( 120-) C - CZ 0.24 2.86 INTRA BL

143 PHE ( 146-) A - CE2 <--> 218 ARG ( 221-) A - NH2 0.23 2.87 INTRA BL

486 ARG ( 85-) C - NH1 <--> 519 LEU ( 118-) C - CD1 0.23 2.87 INTRA BL

62 PHE ( 65-) A - CE2 <--> 64 ARG ( 67-) A - NE 0.23 2.87 INTRA BL

432 ARG ( 31-) C - NH1 <--> 434 GLY ( 33-) C - C 0.23 2.87 INTRA BL

51 SER ( 54-) A - CB <--> 54 ASP ( 57-) A - CG 0.23 2.97 INTRA BL

143 PHE ( 146-) A - CE2 <--> 218 ARG ( 221-) A - NH1 0.23 2.87 INTRA BL

335 ASN ( 338-) A - ND2 <--> 400 LEU ( 403-) A - CD1 0.23 2.87 INTRA BL

28 ARG ( 31-) A - NE <--> 163 GLU ( 166-) A - CG 0.23 2.87 INTRA BL

252 ARG ( 255-) A - NH2 <--> 304 VAL ( 307-) A - CG1 0.23 2.87 INTRA BL

103 LYS ( 106-) A - CG <--> 133 ARG ( 136-) A - NH2 0.23 2.87 INTRA BL

193 GLN ( 196-) A - NE2 <--> 214 PHE ( 217-) A - CE1 0.23 2.87 INTRA BL

521 ARG ( 120-) C - NH2 <--> 529 LEU ( 128-) C - CD2 0.23 2.87 INTRA BL

218 ARG ( 221-) A - NH2 <--> 223 PHE ( 226-) A - CZ 0.23 2.87 INTRA BL

307 PHE ( 310-) A - CD2 <--> 321 PRO ( 324-) A - N 0.23 2.87 INTRA BL

246 ARG ( 249-) A - CA <--> 252 ARG ( 255-) A - NH2 0.23 2.87 INTRA BL

8 ASN ( 11-) A - ND2 <--> 11 VAL ( 14-) A - CG2 0.23 2.87 INTRA BL

489 ASN ( 88-) C - ND2 <--> 518 LYS ( 117-) C - CB 0.23 2.87 INTRA BL

554 CYS ( 153-) C - CB <--> 618 PHE ( 217-) C - CZ 0.23 2.97 INTRA BL

572 TRP ( 171-) C - CZ2 <--> 614 MET ( 213-) C - SD 0.23 3.17 INTRA BL

656 ARG ( 255-) C - NH2 <--> 733 ASP ( 332-) C - CB 0.23 2.87 INTRA BL

170 ARG ( 173-) A - C <--> 172 PRO ( 175-) A - CD 0.23 2.97 INTRA BL

54 ASP ( 57-) A - CG <--> 56 GLU ( 59-) A - CG 0.23 2.97 INTRA BL

547 PHE ( 146-) C - CG <--> 685 THR ( 284-) C - CG2 0.23 2.97 INTRA BL

447 PHE ( 46-) C - CZ <--> 583 ALA ( 182-) C - CB 0.22 2.98 INTRA BL

328 LYS ( 331-) A - C <--> 390 ASP ( 393-) A - CG 0.22 2.98 INTRA BL

439 VAL ( 38-) C - CG1 <--> 569 VAL ( 168-) C - N 0.22 2.88 INTRA BL

99 HIS ( 102-) A - CB <--> 153 LYS ( 156-) A - CE 0.22 2.98 INTRA BL

558 PHE ( 157-) C - CD1 <--> 570 TYR ( 169-) C - CE2 0.22 2.98 INTRA BL

161 ARG ( 164-) A - CG <--> 199 ILE ( 202-) A - CD1 0.22 2.98 INTRA BL

572 TRP ( 171-) C - CZ3 <--> 580 VAL ( 179-) C - CG2 0.22 2.98 INTRA BL

716 TYR ( 315-) C - CE2 <--> 718 TRP ( 317-) C - CG 0.22 2.98 INTRA BL

193 GLN ( 196-) A - NE2 <--> 212 THR ( 215-) A - CG2 0.22 2.88 INTRA BL

439 VAL ( 38-) C - CG1 <--> 568 VAL ( 167-) C - CA 0.22 2.98 INTRA BL

656 ARG ( 255-) C - NH2 <--> 705 PHE ( 304-) C - CD1 0.22 2.88 INTRA BL

293 PHE ( 296-) A - CZ <--> 400 LEU ( 403-) A - CD2 0.22 2.98 INTRA BL

678 LEU ( 277-) C - CD1 <--> 679 PRO ( 278-) C - N 0.21 2.79 INTRA BL

635 CYS ( 234-) C - SG <--> 636 ILE ( 235-) C - N 0.21 2.99 INTRA BL

35 VAL ( 38-) A - CG2 <--> 165 VAL ( 168-) A - N 0.21 2.89 INTRA BL

210 MET ( 213-) A - CE <--> 211 THR ( 214-) A - N 0.21 2.79 INTRA BL

511 HIS ( 110-) C - CG <--> 533 ARG ( 132-) C - CD 0.21 2.99 INTRA BL

727 LYS ( 326-) C - CD <--> 730 LYS ( 329-) C - CE 0.21 2.99 INTRA BL

542 MET ( 141-) C - CE <--> 552 HIS ( 151-) C - CG 0.21 2.99 INTRA BL

542 MET ( 141-) C - CE <--> 552 HIS ( 151-) C - CD2 0.21 2.99 INTRA BL

307 PHE ( 310-) A - CD2 <--> 321 PRO ( 324-) A - CA 0.21 2.99 INTRA BL

99 HIS ( 102-) A - CG <--> 153 LYS ( 156-) A - CE 0.21 2.99 INTRA BL

511 HIS ( 110-) C - CD2 <--> 533 ARG ( 132-) C - CD 0.21 2.99 INTRA BL

704 GLU ( 303-) C - CA <--> 707 THR ( 306-) C - CG2 0.21 2.99 INTRA BL

154 PHE ( 157-) A - CB <--> 210 MET ( 213-) A - CB 0.21 2.99 INTRA BL

328 LYS ( 331-) A - CE <--> 389 ILE ( 392-) A - CB 0.21 2.99 INTRA BL

185 LEU ( 188-) A - CD1 <--> 187 GLN ( 190-) A - N 0.21 2.89 INTRA BL

656 ARG ( 255-) C - NE <--> 705 PHE ( 304-) C - CE1 0.20 2.90 INTRA BL

716 TYR ( 315-) C - CD2 <--> 718 TRP ( 317-) C - N 0.20 2.90 INTRA BL

326 LYS ( 329-) A - CB <--> 386 VAL ( 389-) A - CB 0.20 3.00 INTRA BL

572 TRP ( 171-) C - CH2 <--> 614 MET ( 213-) C - CE 0.20 3.00 INTRA BL

107 HIS ( 110-) A - CD2 <--> 129 ARG ( 132-) A - NH1 0.20 2.90 INTRA BL

382 THR ( 385-) A - CG2 <--> 383 PHE ( 386-) A - N 0.20 2.80 INTRA BL

303 THR ( 306-) A - CG2 <--> 328 LYS ( 331-) A - NZ 0.20 2.90 INTRA BL

582 VAL ( 181-) C - CG1 <--> 583 ALA ( 182-) C - N 0.20 2.80 INTRA BL

161 ARG ( 164-) A - CB <--> 199 ILE ( 202-) A - CD1 0.20 3.00 INTRA BL

711 PHE ( 310-) C - CA <--> 725 PRO ( 324-) C - CD 0.20 3.00 INTRA BL

40 THR ( 43-) A - O <--> 169 THR ( 172-) A - N 0.20 2.50 INTRA BL

506 LYS ( 105-) C - NZ <--> 539 GLU ( 138-) C - CD 0.20 2.90 INTRA BL

701 ALA ( 300-) C - CB <--> 734 PRO ( 333-) C - CB 0.20 3.00 INTRA BL

646 PHE ( 245-) C - CE1 <--> 734 PRO ( 333-) C - CB 0.20 3.00 INTRA BL

307 PHE ( 310-) A - CA <--> 321 PRO ( 324-) A - CD 0.19 3.01 INTRA BL

580 VAL ( 179-) C - CG1 <--> 581 VAL ( 180-) C - N 0.19 2.81 INTRA BL

558 PHE ( 157-) C - CE1 <--> 570 TYR ( 169-) C - CD2 0.19 3.01 INTRA BL

56 GLU ( 59-) A - CB <--> 102 LYS ( 105-) A - NZ 0.19 2.91 INTRA BL

164 VAL ( 167-) A - CG1 <--> 165 VAL ( 168-) A - N 0.19 2.81 INTRA BL

563 TYR ( 162-) C - CD1 <--> 567 GLU ( 166-) C - CG 0.19 3.01 INTRA BL

792 LYS ( 391-) C - O <--> 793 ILE ( 392-) C - CA 0.19 2.21 INTRA BL

793 ILE ( 392-) C - CB <--> 795 ARG ( 394-) C - N 0.19 2.91 INTRA BL

543 HIS ( 142-) C - CD2 <--> 545 GLU ( 144-) C - CG 0.19 3.01 INTRA BL

558 PHE ( 157-) C - CE2 <--> 570 TYR ( 169-) C - CE2 0.19 3.01 INTRA BL

708 VAL ( 307-) C - CG1 <--> 709 ASN ( 308-) C - N 0.19 2.81 INTRA BL

443 LYS ( 42-) C - NZ <--> 571 GLU ( 170-) C - CB 0.19 2.91 INTRA BL

653 VAL ( 252-) C - CG1 <--> 654 PRO ( 253-) C - CD 0.19 3.01 INTRA BL

20 LEU ( 23-) A - CG <--> 90 LYS ( 93-) A - NZ 0.19 2.91 INTRA BL

735 LEU ( 334-) C - C <--> 801 PHE ( 400-) C - CZ 0.19 3.01 INTRA BL

574 ARG ( 173-) C - CG <--> 576 PRO ( 175-) C - CD 0.19 3.01 INTRA BL

704 GLU ( 303-) C - CG <--> 733 ASP ( 332-) C - C 0.19 3.01 INTRA BL

495 ILE ( 94-) C - CG2 <--> 496 TRP ( 95-) C - N 0.19 2.81 INTRA BL

386 VAL ( 389-) A - CG2 <--> 389 ILE ( 392-) A - CD1 0.19 3.01 INTRA BL

168 TRP ( 171-) A - CE2 <--> 210 MET ( 213-) A - CE 0.19 3.01 INTRA BL

241 SER ( 244-) A - CB <--> 330 PRO ( 333-) A - CG 0.19 3.01 INTRA BL

519 LEU ( 118-) C - CD2 <--> 531 THR ( 130-) C - CG2 0.19 3.01 INTRA BL

646 PHE ( 245-) C - N <--> 734 PRO ( 333-) C - CD 0.19 2.91 INTRA BL

242 PHE ( 245-) A - CE1 <--> 330 PRO ( 333-) A - CB 0.19 3.01 INTRA BL

547 PHE ( 146-) C - CE1 <--> 685 THR ( 284-) C - CG2 0.19 3.01 INTRA BL

532 MET ( 131-) C - CB <--> 534 LEU ( 133-) C - CD1 0.19 3.01 INTRA BL

685 THR ( 284-) C - CG2 <--> 686 ALA ( 285-) C - N 0.19 2.81 INTRA BL

61 VAL ( 64-) A - CG2 <--> 62 PHE ( 65-) A - N 0.19 2.81 INTRA BL

465 VAL ( 64-) C - CG2 <--> 466 PHE ( 65-) C - N 0.18 2.82 INTRA BL

648 LEU ( 247-) C - CD1 <--> 649 ASN ( 248-) C - N 0.18 2.82 INTRA BL

176 VAL ( 179-) A - CG2 <--> 177 VAL ( 180-) A - N 0.18 2.82 INTRA BL

91 ILE ( 94-) A - CD1 <--> 92 TRP ( 95-) A - N 0.18 2.82 INTRA BL

722 SER ( 321-) C - CB <--> 725 PRO ( 324-) C - CG 0.18 3.02 INTRA BL

33 GLU ( 36-) A - CG <--> 34 ARG ( 37-) A - N 0.18 2.82 INTRA BL

154 PHE ( 157-) A - CD1 <--> 155 GLY ( 158-) A - N 0.18 2.82 INTRA BL

318 SER ( 321-) A - CB <--> 321 PRO ( 324-) A - CG 0.18 3.02 INTRA BL

574 ARG ( 173-) C - C <--> 576 PRO ( 175-) C - CD 0.18 3.02 INTRA BL

249 VAL ( 252-) A - CG2 <--> 250 PRO ( 253-) A - CD 0.18 3.02 INTRA BL

154 PHE ( 157-) A - CE2 <--> 166 TYR ( 169-) A - CE2 0.18 3.02 INTRA BL

244 LEU ( 247-) A - CD1 <--> 245 ASN ( 248-) A - N 0.18 2.82 INTRA BL

711 PHE ( 310-) C - CD1 <--> 725 PRO ( 324-) C - CB 0.18 3.02 INTRA BL

791 SER ( 390-) C - C <--> 793 ILE ( 392-) C - CA 0.18 3.02 INTRA BL

258 THR ( 261-) A - CG2 <--> 259 THR ( 262-) A - N 0.18 2.82 INTRA BL

672 ILE ( 271-) C - CG2 <--> 673 SER ( 272-) C - N 0.18 2.82 INTRA BL

81 LEU ( 84-) A - CD2 <--> 83 LEU ( 86-) A - CG 0.18 3.02 INTRA BL

646 PHE ( 245-) C - CD1 <--> 734 PRO ( 333-) C - CD 0.18 3.02 INTRA BL

705 PHE ( 304-) C - CZ <--> 709 ASN ( 308-) C - ND2 0.18 2.92 INTRA BL

768 THR ( 367-) C - CG2 <--> 769 ILE ( 368-) C - N 0.18 2.82 INTRA BL

437 GLU ( 36-) C - CG <--> 438 ARG ( 37-) C - N 0.18 2.82 INTRA BL

56 GLU ( 59-) A - CB <--> 102 LYS ( 105-) A - CE 0.18 3.02 INTRA BL

573 THR ( 172-) C - C <--> 575 GLU ( 174-) C - N 0.18 2.72 INTRA BL

368 LYS ( 371-) A - CG <--> 369 GLU ( 372-) A - N 0.18 2.82 INTRA BL

628 VAL ( 227-) C - CG1 <--> 629 ILE ( 228-) C - N 0.18 2.82 INTRA BL

40 THR ( 43-) A - N <--> 169 THR ( 172-) A - N 0.18 2.67 INTRA BL

546 ASP ( 145-) C - CG <--> 550 ASP ( 149-) C - CB 0.18 3.02 INTRA BL

10 THR ( 13-) A - CG2 <--> 11 VAL ( 14-) A - N 0.18 2.82 INTRA BL

664 VAL ( 263-) C - CG1 <--> 665 LEU ( 264-) C - N 0.18 2.82 INTRA BL

414 THR ( 13-) C - CG2 <--> 415 VAL ( 14-) C - N 0.18 2.82 INTRA BL

574 ARG ( 173-) C - CA <--> 580 VAL ( 179-) C - CA 0.18 3.02 INTRA BL

509 VAL ( 108-) C - CG1 <--> 510 ALA ( 109-) C - N 0.18 2.82 INTRA BL

656 ARG ( 255-) C - NH2 <--> 733 ASP ( 332-) C - CG 0.18 2.92 INTRA BL

497 THR ( 96-) C - CG2 <--> 498 PRO ( 97-) C - N 0.18 2.82 INTRA BL

307 PHE ( 310-) A - CD2 <--> 321 PRO ( 324-) A - CB 0.18 3.02 INTRA BL

781 PRO ( 380-) C - C <--> 783 PRO ( 382-) C - CD 0.18 3.02 INTRA BL

736 ILE ( 335-) C - CG2 <--> 801 PHE ( 400-) C - CA 0.18 3.02 INTRA BL

432 ARG ( 31-) C - CD <--> 435 LEU ( 34-) C - CA 0.18 3.02 INTRA BL

222 TYR ( 225-) A - CZ <--> 226 GLN ( 229-) A - CD 0.18 3.02 INTRA BL

456 ASP ( 55-) C - CG <--> 457 HIS ( 56-) C - N 0.18 2.82 INTRA BL

478 PHE ( 77-) C - CD1 <--> 481 PRO ( 80-) C - CD 0.18 3.02 INTRA BL

415 VAL ( 14-) C - CG1 <--> 416 PHE ( 15-) C - N 0.18 2.82 INTRA BL

111 MET ( 114-) A - CG <--> 112 PRO ( 115-) A - CD 0.18 3.02 INTRA BL

35 VAL ( 38-) A - CG2 <--> 36 THR ( 39-) A - N 0.18 2.82 INTRA BL

16 LEU ( 19-) A - CD2 <--> 17 ASP ( 20-) A - N 0.18 2.82 INTRA BL

678 LEU ( 277-) C - CD1 <--> 679 PRO ( 278-) C - CD 0.18 3.02 INTRA BL

257 VAL ( 260-) A - CG1 <--> 258 THR ( 261-) A - N 0.18 2.82 INTRA BL

403 ILE ( 406-) A - CG2 <--> 404 PHE ( 407-) A - N 0.18 2.82 INTRA BL

232 ILE ( 235-) A - CG2 <--> 233 MET ( 236-) A - N 0.18 2.82 INTRA BL

115 LEU ( 118-) A - CD2 <--> 116 LEU ( 119-) A - N 0.17 2.83 INTRA BL

172 PRO ( 175-) A - CD <--> 176 VAL ( 179-) A - CG1 0.17 3.03 INTRA BL

453 PRO ( 52-) C - CD <--> 462 THR ( 61-) C - CB 0.17 3.03 INTRA BL

793 ILE ( 392-) C - N <--> 795 ARG ( 394-) C - N 0.17 2.68 INTRA BL

45 THR ( 48-) A - CG2 <--> 46 SER ( 49-) A - N 0.17 2.83 INTRA BL

268 ILE ( 271-) A - CG2 <--> 269 SER ( 272-) A - N 0.17 2.83 INTRA BL

253 THR ( 256-) A - CG2 <--> 254 VAL ( 257-) A - N 0.17 2.83 INTRA BL

297 ALA ( 300-) A - CB <--> 330 PRO ( 333-) A - CB 0.17 3.03 INTRA BL

735 LEU ( 334-) C - CG <--> 736 ILE ( 335-) C - N 0.17 2.83 INTRA BL

490 LEU ( 89-) C - CD1 <--> 491 MET ( 90-) C - N 0.17 2.83 INTRA BL

623 LYS ( 222-) C - CD <--> 625 GLY ( 224-) C - N 0.17 2.93 INTRA BL

395 ILE ( 398-) A - CG2 <--> 396 ALA ( 399-) A - N 0.17 2.83 INTRA BL

306 TYR ( 309-) A - CB <--> 319 VAL ( 322-) A - CB 0.17 3.03 INTRA BL

225 ILE ( 228-) A - CG2 <--> 226 GLN ( 229-) A - N 0.17 2.83 INTRA BL

289 VAL ( 292-) A - CG2 <--> 290 CYS ( 293-) A - N 0.17 2.83 INTRA BL

685 THR ( 284-) C - N <--> 688 ASP ( 287-) C - CG 0.17 2.93 INTRA BL

693 VAL ( 292-) C - CG2 <--> 694 CYS ( 293-) C - N 0.17 2.83 INTRA BL

86 LEU ( 89-) A - CD1 <--> 87 MET ( 90-) A - N 0.17 2.83 INTRA BL

74 PHE ( 77-) A - CD2 <--> 75 LYS ( 78-) A - N 0.17 2.83 INTRA BL

139 HIS ( 142-) A - NE2 <--> 141 GLU ( 144-) A - CG 0.17 2.93 INTRA BL

149 ALA ( 152-) A - CB <--> 213 HIS ( 216-) A - CE1 0.17 3.03 INTRA BL

711 PHE ( 310-) C - CB <--> 725 PRO ( 324-) C - CD 0.17 3.03 INTRA BL

170 ARG ( 173-) A - CG <--> 172 PRO ( 175-) A - CD 0.17 3.03 INTRA BL

222 TYR ( 225-) A - CE2 <--> 226 GLN ( 229-) A - NE2 0.17 2.93 INTRA BL

136 CYS ( 139-) A - CB <--> 148 HIS ( 151-) A - NE2 0.17 2.93 INTRA BL

139 HIS ( 142-) A - CE1 <--> 141 GLU ( 144-) A - CD 0.17 3.03 INTRA BL

704 GLU ( 303-) C - CG <--> 733 ASP ( 332-) C - N 0.17 2.93 INTRA BL

170 ARG ( 173-) A - CZ <--> 172 PRO ( 175-) A - CG 0.17 3.03 INTRA BL

553 ALA ( 152-) C - CB <--> 617 HIS ( 216-) C - CE1 0.17 3.03 INTRA BL

97 PHE ( 100-) A - CG <--> 98 PHE ( 101-) A - N 0.17 2.83 INTRA BL

107 HIS ( 110-) A - CG <--> 129 ARG ( 132-) A - NH1 0.17 2.93 INTRA BL

646 PHE ( 245-) C - CE1 <--> 801 PHE ( 400-) C - CE1 0.17 3.03 INTRA BL

678 LEU ( 277-) C - CD2 <--> 679 PRO ( 278-) C - CD 0.17 3.03 INTRA BL

325 LYS ( 328-) A - CB <--> 385 SER ( 388-) A - CB 0.17 3.03 INTRA BL

306 TYR ( 309-) A - CD2 <--> 318 SER ( 321-) A - CA 0.17 3.03 INTRA BL

297 ALA ( 300-) A - CA <--> 330 PRO ( 333-) A - CB 0.17 3.03 INTRA BL

234 THR ( 237-) A - CG2 <--> 235 VAL ( 238-) A - N 0.17 2.83 INTRA BL

716 TYR ( 315-) C - CE2 <--> 718 TRP ( 317-) C - CE3 0.17 3.03 INTRA BL

331 LEU ( 334-) A - CD1 <--> 332 ILE ( 335-) A - N 0.17 2.83 INTRA BL

497 THR ( 96-) C - CG2 <--> 498 PRO ( 97-) C - CD 0.17 3.03 INTRA BL

305 ASN ( 308-) A - ND2 <--> 307 PHE ( 310-) A - CB 0.16 2.94 INTRA BL

419 ILE ( 18-) C - CG1 <--> 420 LEU ( 19-) C - N 0.16 2.84 INTRA BL

168 TRP ( 171-) A - CE3 <--> 176 VAL ( 179-) A - CG2 0.16 3.04 INTRA BL

598 THR ( 197-) C - CG2 <--> 599 VAL ( 198-) C - N 0.16 2.84 INTRA BL

478 PHE ( 77-) C - CD1 <--> 480 GLY ( 79-) C - N 0.15 2.95 INTRA BL

23 TYR ( 26-) A - CD2 <--> 71 ARG ( 74-) A - CD 0.15 3.05 INTRA BL

572 TRP ( 171-) C - NE1 <--> 614 MET ( 213-) C - CG 0.15 2.95 INTRA BL

793 ILE ( 392-) C - CD1 <--> 795 ARG ( 394-) C - CG 0.15 3.05 INTRA BL

304 VAL ( 307-) A - CB <--> 305 ASN ( 308-) A - N 0.15 2.55 INTRA BL

686 ALA ( 285-) C - CB <--> 816 TYR ( 415-) C - CE1 0.15 3.05 INTRA BL

547 PHE ( 146-) C - CD2 <--> 685 THR ( 284-) C - CG2 0.15 3.05 INTRA BL

533 ARG ( 132-) C - C <--> 534 LEU ( 133-) C - CD1 0.15 2.95 INTRA BL

331 LEU ( 334-) A - CD1 <--> 332 ILE ( 335-) A - C 0.15 2.95 INTRA BL

574 ARG ( 173-) C - N <--> 575 GLU ( 174-) C - N 0.15 2.45 INTRA BL

107 HIS ( 110-) A - CE1 <--> 129 ARG ( 132-) A - NH1 0.15 2.95 INTRA BL

439 VAL ( 38-) C - CG1 <--> 440 THR ( 39-) C - N 0.15 2.85 INTRA BL

277 VAL ( 280-) A - CG1 <--> 278 ALA ( 281-) A - N 0.15 2.85 INTRA BL

558 PHE ( 157-) C - CD1 <--> 559 GLY ( 158-) C - N 0.15 2.85 INTRA BL

732 LYS ( 331-) C - CB <--> 733 ASP ( 332-) C - N 0.15 2.55 INTRA BL

168 TRP ( 171-) A - O <--> 171 GLU ( 174-) A - CA 0.15 2.65 INTRA BL

712 THR ( 311-) C - N <--> 722 SER ( 321-) C - CB 0.15 2.95 INTRA BL

467 PHE ( 66-) C - CD2 <--> 532 MET ( 131-) C - SD 0.15 3.25 INTRA BL

69 ASP ( 72-) A - CG <--> 72 LEU ( 75-) A - CD2 0.15 3.05 INTRA BL

199 ILE ( 202-) A - CD1 <--> 206 GLU ( 209-) A - CD 0.15 3.05 INTRA BL

And so on for a total of 1126 lines.

# 61 # Note: Some notes regarding these bumps

The bumps have been binned in 5 categories ranging from 'should deal with'

till 'must fix'. Additionally, the integrated sum of all bumps, the squared

sum of all bumps, and these latter two values normalized by the number of

contacts are listed too for comparison purposes between, for example, small

and large proteins.

Total bump value: 159.736

Total bump value per residue: 1.378

Total number of bumps: 1126

Total squared bump value: 32.669

Total number of bumps in the mildest bin: 880

Total number of bumps in the second bin: 246

Total number of bumps in the middle bin: 0

Total number of bumps in the fourth bin: 0

Total number of bumps in the worst bin: 0

# 62 # Note: Inside/Outside residue distribution normal

The distribution of residue types over the inside and the outside of the

protein is normal.

inside/outside RMS Z-score : 1.124

Content of the SOUP. See the writeup for an explanation.

Molecule Range Type Set name

1 1 ( 4) 413 ( 416)A Protein checkset

2 414 ( 13) 817 ( 416)C Protein checkset

# 63 # Note: Inside/Outside RMS Z-score plot

The Inside/Outside distribution normality RMS Z-score over a 15 residue

window is plotted as function of the residue number. High areas in the plot

(above 1.5) indicate unusual inside/outside patterns.

In the TeX file, a plot has been inserted here

Chain identifier: A

# 64 # Note: Inside/Outside RMS Z-score plot

In the TeX file, a plot has been inserted here

Chain identifier: C

# 65 # Warning: Abnormal packing environment for some residues

The residues listed in the table below have an unusual packing environment.

The packing environment of the residues is compared with the average packing

environment for all residues of the same type in good PDB files. A low

packing score can indicate one of several things: Poor packing, misthreading

of the sequence through the density, crystal contacts, contacts with a

co-factor, or the residue is part of the active site. It is not uncommon to

see a few of these, but in any case this requires further inspection of the

residue.

749 TYR ( 348-) C - -8.86

345 TYR ( 348-) A - -8.62

338 TYR ( 341-) A - -8.59

482 MET ( 81-) C - -7.60

742 TYR ( 341-) C - -7.26

78 MET ( 81-) A - -6.99

787 PHE ( 386-) C - -6.92

779 LYS ( 378-) C - -6.92

309 LYS ( 312-) A - -6.77

184 ARG ( 187-) A - -6.68

760 LEU ( 359-) C - -6.64

109 MET ( 112-) A - -6.63

713 LYS ( 312-) C - -6.61

755 ARG ( 354-) C - -6.59

75 LYS ( 78-) A - -6.57

772 LYS ( 371-) C - -6.50

561 TYR ( 160-) C - -6.33

721 LYS ( 320-) C - -6.26

513 MET ( 112-) C - -6.26

375 LYS ( 378-) A - -6.25

479 LYS ( 78-) C - -6.22

381 LYS ( 384-) A - -6.15

157 TYR ( 160-) A - -6.13

716 TYR ( 315-) C - -6.13

588 ARG ( 187-) C - -6.07

170 ARG ( 173-) A - -6.06

359 ILE ( 362-) A - -5.94

647 TRP ( 246-) C - -5.89

312 TYR ( 315-) A - -5.85

765 LYS ( 364-) C - -5.79

310 ARG ( 313-) A - -5.71

243 TRP ( 246-) A - -5.66

438 ARG ( 37-) C - -5.66

714 ARG ( 313-) C - -5.63

34 ARG ( 37-) A - -5.62

351 ARG ( 354-) A - -5.60

769 ILE ( 368-) C - -5.50

774 VAL ( 373-) C - -5.45

784 LYS ( 383-) C - -5.21

314 TRP ( 317-) A - -5.19

348 ASN ( 351-) A - -5.18

349 LEU ( 352-) A - -5.15

2 GLN ( 5-) A - -5.14

718 TRP ( 317-) C - -5.12

432 ARG ( 31-) C - -5.11

26 ARG ( 29-) A - -5.10

28 ARG ( 31-) A - -5.06

191 LEU ( 194-) A - -5.03

630 GLN ( 229-) C - -5.03

370 VAL ( 373-) A - -5.02

333 LYS ( 336-) A - -5.02

322 GLU ( 325-) A - -5.00

# 66 # Warning: Abnormal packing environment for sequential residues

A stretch of at least three sequential residues with a questionable packing

environment was found. This could indicate that these residues are part

of a strange loop. It might also be an indication of misthreading in the

density. However, it can also indicate that one or more residues in this

stretch have other problems such as, for example, missing atoms, very

weird angles or bond lengths, etc.

The table below lists the first and last residue in each stretch found,

as well as the average residue score of the series.

109 MET ( 112-) A - 111 --- MET 114- (A ) - -5.14

348 ASN ( 351-) A - 351 --- ARG 354- (A ) - -5.07

364 THR ( 367-) A - 366 --- GLU 369- (A ) - -4.42

753 LEU ( 352-) C - 757 --- ASP 356- (C ) - -4.94

777 GLU ( 376-) C - 779 --- LYS 378- (C ) - -5.19

# 67 # Note: Structural average packing environment OK

The structural average packing score is within normal ranges.

Average for range 1 - 817 : -1.314

# 68 # Note: Quality value plot

The quality value smoothed over a 10 residue window is plotted as function

of the residue number. Low areas in the plot (below -2.0) indicate unusual

packing.

In the TeX file, a plot has been inserted here

Chain identifier: A

# 69 # Note: Quality value plot

The quality value smoothed over a 10 residue window is plotted as function

of the residue number. Low areas in the plot (below -2.0) indicate unusual

packing.

In the TeX file, a plot has been inserted here

Chain identifier: C

# 70 # Warning: Low packing Z-score for some residues

The residues listed in the table below have an unusual packing

environment according to the 2nd generation packing check. The score

listed in the table is a packing normality Z-score: positive means

better than average, negative means worse than average. Only residues

scoring less than -2.50 are listed here. These are the unusual

residues in the structure, so it will be interesting to take a

special look at them.

76 GLY ( 79-) A - -2.71

33 GLU ( 36-) A - -2.54

437 GLU ( 36-) C - -2.54

# 71 # Note: No series of residues with abnormal new packing environment

There are no stretches of four or more residues each having a packing

Z-score worse than -1.75.

Content of the SOUP. See the writeup for an explanation.

Molecule Range Type Set name

1 1 ( 4) 413 ( 416)A Protein checkset

2 414 ( 13) 817 ( 416)C Protein checkset

# 72 # Note: Second generation quality Z-score plot

The second generation quality Z-score smoothed over a 10 residue window

is plotted as function of the residue number. Low areas in the plot (below

-1.3) indicate unusual packing.

In the TeX file, a plot has been inserted here

Chain identifier: A

# 73 # Note: Second generation quality Z-score plot

In the TeX file, a plot has been inserted here

Chain identifier: C

# 74 # Error: His, Asn, Gln side chain flips

Listed here are Histidine, Asparagine or Glutamine residues for

which the orientation determined from hydrogen bonding analysis are

different from the assignment given in the input. Either they could

form energetically more favourable hydrogen bonds if the terminal

group was rotated by 180 degrees, or there is no assignment in the

input file (atom type 'A') but an assignment could be made. Be aware,

though, that if the topology could not be determined for one or more

ligands, then this option will make errors.

25 ASN ( 28-) A -

148 HIS ( 151-) A -

193 GLN ( 196-) A -

429 ASN ( 28-) C -

457 HIS ( 56-) C -

469 GLN ( 68-) C -

597 GLN ( 196-) C -

676 ASN ( 275-) C -

# 75 # Note: Histidine type assignments

For all complete HIS residues in the structure a tentative assignment to

HIS-D (protonated on ND1), HIS-E (protonated on NE2), or HIS-H (protonated

on both ND1 and NE2, positively charged) is made based on the hydrogen bond

network. A second assignment is made based on which of the Engh and Huber

[REF] histidine geometries fits best to the structure.

In the table below all normal histidine residues are listed. The assignment

based on the geometry of the residue is listed first, together with the RMS

Z-score for the fit to the Engh and Huber parameters. For all residues where

the H-bond assignment is different, the assignment is listed in the last

columns, together with its RMS Z-score to the Engh and Huber parameters.

As always, the RMS Z-scores should be close to 1.0 if the residues were

restrained to the Engh and Huber parameters during refinement, and if

enough (high resolution) data is available.

Please note that because the differences between the geometries of the

different types are small it is possible that the geometric assignment given

here does not correspond to the type used in refinement. This is especially

true if the RMS Z-scores are much higher than 1.0.

If the two assignments differ, or the `geometry' RMS Z-score is high, it is

advisable to verify the hydrogen bond assignment, check the HIS type used

during the refinement and possibly adjust it.

53 HIS ( 56-) A - HIS-D 0.36 HIS-E 1.47

99 HIS ( 102-) A - HIS-D 0.34 HIS-E 1.43

107 HIS ( 110-) A - HIS-D 0.32 HIS-E 1.43

139 HIS ( 142-) A - HIS-D 0.32 HIS-E 1.43

148 HIS ( 151-) A - HIS-D 0.28 HIS-E 1.40

213 HIS ( 216-) A - HIS-D 0.37 HIS-E 1.44

215 HIS ( 218-) A - HIS-D 0.38 HIS-E 1.44

457 HIS ( 56-) C - HIS-D 0.35

503 HIS ( 102-) C - HIS-D 0.38 HIS-E 1.46

511 HIS ( 110-) C - HIS-D 0.35 HIS-E 1.49

543 HIS ( 142-) C - HIS-D 0.34 HIS-E 1.46

552 HIS ( 151-) C - HIS-D 0.36 HIS-E 1.44

617 HIS ( 216-) C - HIS-D 0.31 HIS-E 1.39

619 HIS ( 218-) C - HIS-D 0.34 HIS-E 1.45

# 76 # Warning: Buried unsatisfied hydrogen bond donors

The buried hydrogen bond donors listed in the table below have a hydrogen

atom that is not involved in a hydrogen bond in the optimized hydrogen bond

network.

Hydrogen bond donors that are buried inside the protein normally use all of

their hydrogens to form hydrogen bonds within the protein. If there are any

non hydrogen bonded buried hydrogen bond donors in the structure they will

be listed here. In very good structures the number of listed atoms will tend

to zero.

Waters are not listed by this option.

11 VAL ( 14-) A - N

40 THR ( 43-) A - OG1

54 ASP ( 57-) A - N

67 TRP ( 70-) A - NE1

71 ARG ( 74-) A - NH1

75 LYS ( 78-) A - N

79 THR ( 82-) A - N

86 LEU ( 89-) A - N

98 PHE ( 101-) A - N

116 LEU ( 119-) A - N

123 THR ( 126-) A - N

127 THR ( 130-) A - OG1

141 GLU ( 144-) A - N

142 ASP ( 145-) A - N

146 ASP ( 149-) A - N

168 TRP ( 171-) A - N

168 TRP ( 171-) A - NE1

169 THR ( 172-) A - N

177 VAL ( 180-) A - N

180 GLU ( 183-) A - N

188 TYR ( 191-) A - N

218 ARG ( 221-) A - NH2

246 ARG ( 249-) A - N

247 GLU ( 250-) A - N

252 ARG ( 255-) A - NH1

252 ARG ( 255-) A - NH2

284 ASP ( 287-) A - N

314 TRP ( 317-) A - N

319 VAL ( 322-) A - N

322 GLU ( 325-) A - N

326 LYS ( 329-) A - N

328 LYS ( 331-) A - N

332 ILE ( 335-) A - N

341 THR ( 344-) A - N

386 VAL ( 389-) A - N

415 VAL ( 14-) C - N

416 PHE ( 15-) C - N

417 THR ( 16-) C - N

432 ARG ( 31-) C - NE

440 THR ( 39-) C - OG1

457 HIS ( 56-) C - N

458 ASP ( 57-) C - N

462 THR ( 61-) C - OG1

471 TRP ( 70-) C - NE1

475 ARG ( 74-) C - NH1

480 GLY ( 79-) C - N

502 PHE ( 101-) C - N

545 GLU ( 144-) C - N

570 TYR ( 169-) C - N

573 THR ( 172-) C - OG1

574 ARG ( 173-) C - N

577 ALA ( 176-) C - N

581 VAL ( 180-) C - N

587 SER ( 186-) C - OG

592 TYR ( 191-) C - N

597 GLN ( 196-) C - NE2

609 GLY ( 208-) C - N

612 VAL ( 211-) C - N

625 GLY ( 224-) C - N

656 ARG ( 255-) C - NE

656 ARG ( 255-) C - NH2

683 TYR ( 282-) C - N

685 THR ( 284-) C - N

718 TRP ( 317-) C - N

722 SER ( 321-) C - OG

723 VAL ( 322-) C - N

732 LYS ( 331-) C - N

736 ILE ( 335-) C - N

769 ILE ( 368-) C - N

789 SER ( 388-) C - OG

793 ILE ( 392-) C - N

794 ASP ( 393-) C - N

795 ARG ( 394-) C - N

798 ARG ( 397-) C - NE

# 77 # Warning: Buried unsatisfied hydrogen bond acceptors

The buried side-chain hydrogen bond acceptors listed in the table below are

not involved in a hydrogen bond in the optimized hydrogen bond network.

Side-chain hydrogen bond acceptors buried inside the protein normally form

hydrogen bonds within the protein. If there are any not hydrogen bonded in

the optimized hydrogen bond network they will be listed here.

Waters are not listed by this option.

52 ASP ( 55-) A - OD1

52 ASP ( 55-) A - OD2

56 GLU ( 59-) A - OE1

146 ASP ( 149-) A - OD2

148 HIS ( 151-) A - ND1

163 GLU ( 166-) A - OE1

163 GLU ( 166-) A - OE2

300 GLU ( 303-) A - OE1

384 ASN ( 387-) A - OD1

405 ASN ( 408-) A - OD1

460 GLU ( 59-) C - OE1

489 ASN ( 88-) C - OD1

610 GLU ( 209-) C - OE1

733 ASP ( 332-) C - OD2

794 ASP ( 393-) C - OD1

# 78 # Note: Some notes regarding these donors and acceptors

The donors and acceptors have been counted, also as function of their

accessibility. The buried donors and acceptors have been binned in five

categories ranging from not forming any hydrogen bond till forming a poor

till perfect hydrogen bond. Obviously, the buried donors and acceptors

with no or just a poor hydrogen bond should be a topic of concern. As every

protein contains more acceptors than donors, unsatisfied donors are more in

need of attention than unsatisfied acceptors.

Total number of donors: 1200

of which buried: 479

Total number of acceptors: 1233

of which buried: 425

Total number of donor+acceptors: 165

(e.g. the Ser Ogamma that can donate and accept)

of which buried: 21

Buried donors: 479

without H-bond: 69

essentially without H-bond: 1

with only a very poor H-bond: 1

with a poor H-bond: 10

with a H-bond: 398

Buried acceptors: 425

without H-bond: 80

essentially without H-bond: 0

with only a very poor H-bond: 0

with a poor H-bond: 7

with a H-bond: 338

# 79 # Warning: No crystallisation information

No, or very inadequate, crystallisation information was observed upon

reading the PDB file header records. This information should be available

in the form of a series of REMARK 280 lines. Without this information a

few things, such as checking ions in the structure, cannot be performed

optimally.

# 80 # Note: No ions (of a type we can validate) in structure

Since there are no ions in the structure of a type we can validate, this

check will not be executed.

Since there are no waters, the water check has been skipped.

# 81 # Note: Content of the PDB file as interpreted by WHAT CHECK

Content of the PDB file as interpreted by WHAT CHECK.

WHAT CHECK has read your PDB file, and stored it internally in what is called

'the soup'. The content of this soup is listed here. An extensive explanation

of all frequently used WHAT CHECK output formats can be found at

swift.cmbi.ru.nl. Look under output formats. A course on reading this

'Molecules' table is part of the WHAT CHECK website.

1 1 ( 4) 413 ( 416) A Protein checkset

2 414 ( 13) 817 ( 416) C Protein checkset

# 82 # Note: Summary report

This is an overall summary of the quality of the structure as compared with

current reliable structures. Numbers in brackets are the average and standard

deviation observed for a large number of files determined with a similar

resolution.

The second table mostly gives an impression of how well the model conforms

to common refinement restraint values. These numbers are less than 1.0 if the

spread in data is too little, and larger than 1.0 when the spread is too

large. The former does not need to be a problem, the latter always is bad.

Structure Z-scores, positive is better than average:

Resolution read from PDB file : -1.000

1st generation packing quality : -2.036

2nd generation packing quality : -3.210 (poor)

Ramachandran plot appearance : -0.184

chi-1/chi-2 rotamer normality : -2.241

Backbone conformation : 0.054

Inside/Outside distribution : 1.124

RMS Z-scores, should be close to 1.0:

Bond lengths : 0.949

Bond angles : 1.350

Omega angle restraints : 0.879

Side chain planarity : 0.395 (tight)

Improper dihedral distribution : 1.290

# 83 # Note: Introduction to refinement recommendations

First, be aware that the recommendations for crystallographers listed below

are produced by a computer program that was written by a guy who got his

PhD in NMR...

We have tried to convert the messages written in this report into a small

set of things you can do with your refinement software to get a better

structure. The things you should do first are listed first. And in some

cases you should first fix that problem, then refine a bit further, and

then run WHAT CHECK again before looking at other problems. If, for example,

WHAT CHECK has found a problem with the SCALE and CRYST cards, then you must

first fix that problem, refine the structure a bit further, and run WHAT

CHECK again because errors in the SCALE and or CRYST card can lead to many

problems elsewhere in the validation process.

It is also important to keep in mind that WHAT CHECK is software and that it

occasionally totally misunderstands what is the cause of a problem. But, if

WHAT CHECK lists a problem there normally is a problem albeit that it not

always is the actual problem that gets listed.

# 84 # Note: No crippling problems detected

Some problems can be so crippling that they negatively influence the

validity of other validation steps. If such a problem is detected, it must

be solved and some further refinemnet must be done before you can continue

working with a new WHAT CHECK report. In this file such problems were not

detected. You can therefore try to fix as many problems in one go as you

want.

# 85 # Note: No resolution information detected

WHAT CHECK needs to know the resolution of your data to provide advice

for the refinement process. This resolution information is needed because

a Z-score that is very good at 1.0 Angstrom resolution might actually be

a sign of over-refinement at 3.5 Angstrom, etcetera. So, take a look at

the formats of REMARK 2 and/or REMARK 3 and put the resolution in ether

of those 2. An example of a REMARK 2 card is:

REMARK 2 RESOLUTION. 4.50 ANGSTROMS.

==============

WHAT IF

G.Vriend,

WHAT IF: a molecular modelling and drug design program,

J. Mol. Graph. 8, 52--56 (1990).

WHAT_CHECK (verification routines from WHAT IF)

R.W.W.Hooft, G.Vriend, C.Sander and E.E.Abola,

Errors in protein structures

Nature 381, 272 (1996).

(see also http://swift.cmbi.ru.nl/gv/whatcheck for a course and extra

information)

Bond lengths and angles, protein residues

R.Engh and R.Huber,

Accurate bond and angle parameters for X-ray protein structure

refinement,

Acta Crystallogr. A47, 392--400 (1991).

Bond lengths and angles, DNA/RNA

G.Parkinson, J.Voitechovsky, L.Clowney, A.T.Bruenger and H.Berman,

New parameters for the refinement of nucleic acid-containing structures

Acta Crystallogr. D52, 57--64 (1996).

DSSP

W.Kabsch and C.Sander,

Dictionary of protein secondary structure: pattern

recognition of hydrogen bond and geometrical features

Biopolymers 22, 2577--2637 (1983).

Hydrogen bond networks

R.W.W.Hooft, C.Sander and G.Vriend,

Positioning hydrogen atoms by optimizing hydrogen bond networks in

protein structures

PROTEINS, 26, 363--376 (1996).

Matthews' Coefficient

B.W.Matthews

Solvent content of Protein Crystals

J. Mol. Biol. 33, 491--497 (1968).

Protein side chain planarity

R.W.W. Hooft, C. Sander and G. Vriend,

Verification of protein structures: side-chain planarity

J. Appl. Cryst. 29, 714--716 (1996).

Puckering parameters

D.Cremer and J.A.Pople,

A general definition of ring puckering coordinates

J. Am. Chem. Soc. 97, 1354--1358 (1975).

Quality Control

G.Vriend and C.Sander,

Quality control of protein models: directional atomic

contact analysis,

J. Appl. Cryst. 26, 47--60 (1993).

Ramachandran plot

G.N.Ramachandran, C.Ramakrishnan and V.Sasisekharan,

Stereochemistry of Polypeptide Chain Conformations

J. Mol. Biol. 7, 95--99 (1963).

R.W.W. Hooft, C.Sander and G.Vriend,

Objectively judging the quality of a protein structure from a

Ramachandran plot

CABIOS (1997), 13, 425--430.

Symmetry Checks

R.W.W.Hooft, C.Sander and G.Vriend,

Reconstruction of symmetry related molecules from protein

data bank (PDB) files

J. Appl. Cryst. 27, 1006--1009 (1994).

Tau angle

W.G.Touw and G.Vriend

On the complexity of Engh and Huber refinement restraints: the angle

tau as example.

Acta Crystallogr D 66, 1341--1350 (2010).

Ion Checks

I.D.Brown and K.K.Wu,

Empirical Parameters for Calculating Cation-Oxygen Bond Valences

Acta Cryst. B32, 1957--1959 (1975).

M.Nayal and E.Di Cera,

Valence Screening of Water in Protein Crystals Reveals Potential Na+

Binding Sites

J.Mol.Biol. 256 228--234 (1996).

P.Mueller, S.Koepke and G.M.Sheldrick,

Is the bond-valence method able to identify metal atoms in protein

structures?

Acta Cryst. D 59 32--37 (2003).

Checking checks

K.Wilson, C.Sander, R.W.W.Hooft, G.Vriend, et al.

Who checks the checkers

J.Mol.Biol. (1998) 276,417-436.

/home/vriend/whatif/dbdata/pdbout2html

After running WHAT IF's WHAT CHECK option many things have happened to

the data structure that might not be optimal for many other options.

FULCHK therefore is a so-called terminal option, i.e. after running

the validation option, WHAT IF will restart without any coordinates

in the soup; so the molecule you just checked got deleted together

with anything else you might have had in the SOUP.

Option not found, try:%NO

For obvious reasons $ commands are not allowed in WWW scripts.

WHAT IF detected a $ in a WWW script. The command will be

listed below. If this command contains something that is

potentially harmful to your environment, please mail

G Vriend (Vriend@cmbi.kun.nl) which $ command was

detected, and from where you got this script.

Option:$/home/vriend/whatif/dbdata/pdbout2html

ERROR. Trying to close non-opened log file

==============

WHAT IF

G.Vriend,

WHAT IF: a molecular modelling and drug design program,

J. Mol. Graph. 8, 52--56 (1990).

WHAT_CHECK (verification routines from WHAT IF)

R.W.W.Hooft, G.Vriend, C.Sander and E.E.Abola,

Errors in protein structures

Nature 381, 272 (1996).

(see also http://swift.cmbi.ru.nl/gv/whatcheck for a course and extra

information)

Bond lengths and angles, protein residues

R.Engh and R.Huber,

Accurate bond and angle parameters for X-ray protein structure

refinement,

Acta Crystallogr. A47, 392--400 (1991).

Bond lengths and angles, DNA/RNA

G.Parkinson, J.Voitechovsky, L.Clowney, A.T.Bruenger and H.Berman,

New parameters for the refinement of nucleic acid-containing structures

Acta Crystallogr. D52, 57--64 (1996).

DSSP

W.Kabsch and C.Sander,

Dictionary of protein secondary structure: pattern

recognition of hydrogen bond and geometrical features

Biopolymers 22, 2577--2637 (1983).

Hydrogen bond networks

R.W.W.Hooft, C.Sander and G.Vriend,

Positioning hydrogen atoms by optimizing hydrogen bond networks in

protein structures

PROTEINS, 26, 363--376 (1996).

Matthews' Coefficient

B.W.Matthews

Solvent content of Protein Crystals

J. Mol. Biol. 33, 491--497 (1968).

Protein side chain planarity

R.W.W. Hooft, C. Sander and G. Vriend,

Verification of protein structures: side-chain planarity

J. Appl. Cryst. 29, 714--716 (1996).

Puckering parameters

D.Cremer and J.A.Pople,

A general definition of ring puckering coordinates

J. Am. Chem. Soc. 97, 1354--1358 (1975).

Quality Control

G.Vriend and C.Sander,

Quality control of protein models: directional atomic

contact analysis,

J. Appl. Cryst. 26, 47--60 (1993).

Ramachandran plot

G.N.Ramachandran, C.Ramakrishnan and V.Sasisekharan,

Stereochemistry of Polypeptide Chain Conformations

J. Mol. Biol. 7, 95--99 (1963).

R.W.W. Hooft, C.Sander and G.Vriend,

Objectively judging the quality of a protein structure from a

Ramachandran plot

CABIOS (1997), 13, 425--430.

Symmetry Checks

R.W.W.Hooft, C.Sander and G.Vriend,

Reconstruction of symmetry related molecules from protein

data bank (PDB) files

J. Appl. Cryst. 27, 1006--1009 (1994).

Tau angle

W.G.Touw and G.Vriend

On the complexity of Engh and Huber refinement restraints: the angle

tau as example.

Acta Crystallogr D 66, 1341--1350 (2010).

Ion Checks

I.D.Brown and K.K.Wu,

Empirical Parameters for Calculating Cation-Oxygen Bond Valences

Acta Cryst. B32, 1957--1959 (1975).

M.Nayal and E.Di Cera,

Valence Screening of Water in Protein Crystals Reveals Potential Na+

Binding Sites

J.Mol.Biol. 256 228--234 (1996).

P.Mueller, S.Koepke and G.M.Sheldrick,

Is the bond-valence method able to identify metal atoms in protein

structures?

Acta Cryst. D 59 32--37 (2003).

Checking checks

K.Wilson, C.Sander, R.W.W.Hooft, G.Vriend, et al.

Who checks the checkers

J.Mol.Biol. (1998) 276,417-436.
